# Solvent- and Catalyst-Free Synthesis of *gem*-Difluorinated and Polyfluoroarylated Compounds with Nucleophilic or Electrophilic Fluorine-Containing Reaction Partners, Respectively

**DOI:** 10.3390/molecules29030697

**Published:** 2024-02-02

**Authors:** Lingheng Li, Jinshan Li

**Affiliations:** 1 Department of Photography, Tianjin University of Technology, Tianjin 300384, China; 2 School of Chemistry and Chemical Engineering, Hainan University, Haikou 570228, China

**Keywords:** solvent-free, catalyst-free, gem-difluorinated compounds, polyfluoroarylated compounds

## Abstract

A novel, efficient and environmentally friendly solvent-free and catalyst-free approach for the synthesis of structurally diverse *gem*-difluorinated and polyfluoroarylated derivatives with readily available nucleophilic and electrophilic fluorine-containing reaction partners, difluoroenoxysilane and pentafluorobenzaldehyde, is described. This neat protocol is induced by the direct hydrogen-bond interactions between fluorinated and non-fluorinated reactants without the use of heavy metal catalysts or volatile organic solvents and with no need for column chromatographic separation for most cases.

## 1. Introduction

The last decade has witnessed growing interest in developing new solvent- and catalyst-free organic transformations among researchers both in academia and industry [1,2,3]. This is not only because of the increased demand for sustainable and environmentally benign synthetic approaches in green synthesis, but also because solvent and catalyst-free synthesis could tremendously accelerate the reaction rates, minimize the side reactions caused by the solvents and avoid the use of expensive, metallic and corrosive catalysts. Furthermore, when reactions are conducted under solvent-free conditions, solvation phenomena will not need to be considered, which may provide an opportunity for chemists to realize the reactions that could not be achieved in conventional solvents before. Therefore, developing novel reactions without requiring the usage of solvents and catalysts is an important subject in green chemistry [4,5,6].

**Figure 1:**
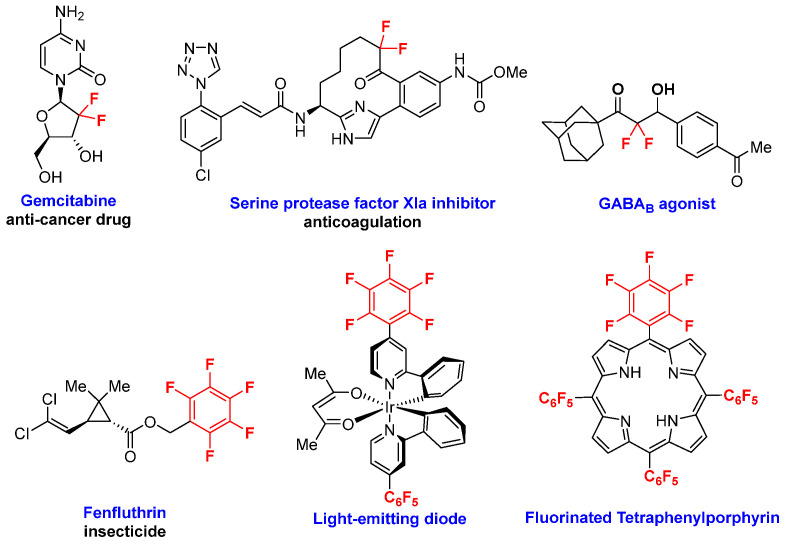
Representative functional molecules containing *gem*-difluoromethylene and perfluorinated arene scaffolds.

The widespread applications of fluorine-containing organic molecules and their derivatives in pharmaceuticals, agrochemicals, polymers, dyes, and optical functional materials have raised demand greatly in recent years [7,8]. Up to now, a series of synthetic methods for accessing new fluorine-containing compounds have been developed [9,10,11]. Among the existing organofluorine compounds, *gem*-difluoromethylene and perfluorinated arene-containing molecules have long been recognized as fascinating targets for organic synthesis due to their special structures and important properties (Figure 1) [12,13,14].

**Scheme 1:**
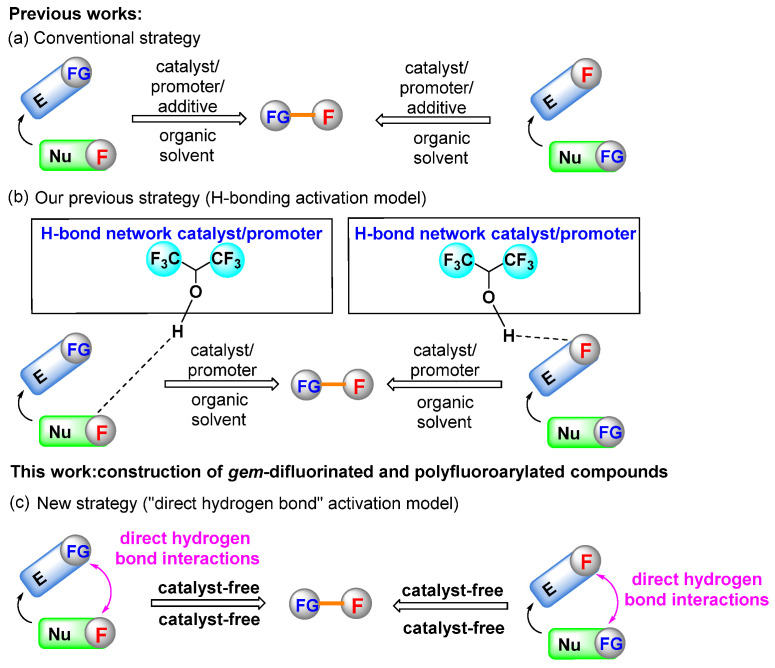
Synthetic strategies for the construction of organofluorine compounds.

Accordingly, two general approaches have been developed for the construction of such compounds [12,15]. The first one is the nucleophilic difluoroalkylation and perfluoroarylation of electrophilic acceptors, and the second strategy is based on the addition of different nucleophiles to difluoromethylene- and perfluorinated arene-containing reaction partners. Due to the low reactivities of most fluorine-containing reactants, catalysts, promoters or additives are essential for enabling the reactions to proceed smoothly (Scheme 1a) [16,17,18,19]. Although significant progress has been made, these methodologies usually show some drawbacks such as requiring expensive metal Lewis acid catalysts, strong acid catalysts, volatile organic solvents, relatively high temperatures and longer reaction periods. To further activate fluorine-containing reactants under mild conditions, a new activation model for nucleophilic difluoroenoxysilane and electrophilic difluoroacetaldehyde ethyl hemiacetal has been developed by our group, using 1,1,1,3,3,3-hexafluoro-2-propanol (HFIP) as the organocatalyst or promoter in various cases due to its high H-bond donating ability, mild acidity and strong cation stabilization (Scheme 1b) [20,21]. In those processes, fluorine-containing reactants were activated via strong hydrogen bonding and the competing side reactions were significantly suppressed. In addition, it should be pointed out that although quite a number of neat reactions have been developed to generate structurally diverse molecules over the past decades, reports on the efficient construction of fluorine-containing organic molecules without solvent or catalyst are fairly limited [22,23,24,25]. Thus, considering the formation of hydrogen bonds between the reactants and HFIP, we were interested in determining whether the non-fluorinated reactants could be activated by fluorinated reactants directly through the intermolecular hydrogen-bond interactions without the participation of additional catalysts (Scheme 1c). In this context, we report a novel solvent- and catalyst-free method for the efficient synthesis of *gem*-difluorinated and polyfluoroarylated compounds with nucleophilic and electrophilic fluorine-containing reaction partners. To the best of our knowledge, this is the first report on the direct reaction with nucleophilic difluoroenoxysilane and electrophilic pentafluorobenzaldehyde in neat conditions without a solvent and catalyst.

## 2. Results and Discussion

**Figure 2:**
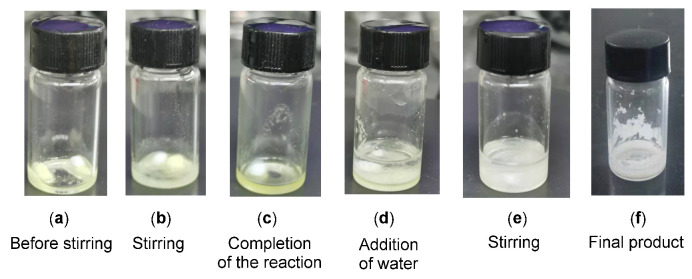
The main reaction process for the synthesis of compound **3a**.

To find out the feasibility of the direct synthesis of *gem*-difluorinated compounds using nucleophilic difluoroenoxysilanes under solvent- and catalyst-free conditions, the reaction of glyoxal monohydrate **1a** with difluoroenoxysilane **2a** was chosen as a model reaction. To our delight, the reaction under neat conditions using 2.0 equiv. of **2a** at room temperature (Table 1, entry 1) led to the direct formation of *gem*-difluorinated 2-hydroxy-1,4-dicarbonyl adduct **3a** in 91% yield, smoothly, after only 4 h. The reaction time was greatly reduced in comparison with our previous experiment results, using HFIP as the catalyst and DCM as solvent [26]. Then, a reduced amount of difluoroenoxysilane **2a** (1.0 equiv, entry 2) was investigated to obtain a higher reaction utilization. The desired product, **3a**, was obtained in 89% yield. A reaction at a higher temperature (60 °C) by reducing the reaction duration was not helpful due to the partial decomposition of the both reactants (entry 3). These studies have shown that considerable rate acceleration is formed under neat conditions, compared to the reported results in organic solvents with different catalysts. Further, the reactions in organic solvents and water (entries 4–9) delivered the *gem*-difluorinated products in rather low yields. On the basis of these investigations, reactions under neat conditions using 1.0 equiv. of difluoroenoxysilane were chosen as optimized conditions for direct nucleophilic addition reactions at room temperature. More importantly, this solvent-free procedure with minimal usage of difluoroenoxysilanes makes the separation and purification of the final products even easier using only simple filtration with an H_2_O work-up (Figure 2 shows the main reaction process; see the Supplementary Materials for details). Finally, for comparison, several Lewis acid and Brønsted acid catalysts such as Sc(OTf)_3_, PhB(OH)_2_ and PTSA were tested in DCM with 10 mol % amount and the desired product was obtained in 13–42% yield, which showed the superiority of the developed neat reactions in the absence of catalysts.

**Scheme 2:**
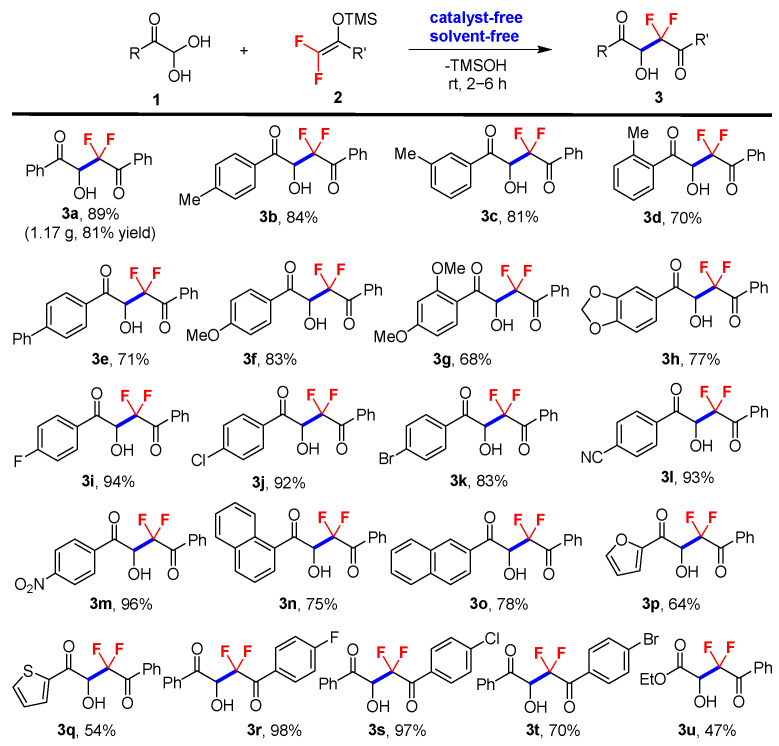
Standard reaction conditions: glyoxal monohydrates **1** (1.0 mmol) and difluoroenoxysilanes **2** (1.0 mmol) at room temperature. The yields are isolated yields.

The scope of the solvent- and catalyst-free difluoroalkylation reaction was then explored with a range of glyoxal monohydrates **1**, including aromatic (**1a**–**1o**), heteroaromatic (**1p**–**1q**), aliphatic derivatives (**1u**), and difluoroenoxysilane derivatives **2**, as outlined in Scheme 2. The results indicate that the neat reaction is highly efficient (2–6 h of reaction time) for all series of reactants, proceeding at room temperature to afford *gem*-difluorinated 2-hydroxy-1,4-dicarbonyl products **3** in moderate to good yields and with high purity, without the need for chromatographic purification in most cases. It was found that electron-withdrawing groups which substituted glyoxal monohydrates (**1i**–**1j** and **1l**–**1m**) afforded the desired products in higher yield than the substrates with electron-donating groups (**1b**–**1h**). And this neat reaction was found to be sensitive to the steric effect. *Ortho*-substituted phenyl glyoxal monohydrate gave a much lower yield (**3d**, 70% yield) compared with the *para*- and *meta*-substituted substrates (**3b**, 84%; **3c**, 81%). Finally, a gram-scale synthesis of **3a** was conducted under the neat conditions without the need for chromatographic purification to further illustrate the practicability of this solvent- and catalyst-free reaction (5.0 mmol scale, 1.17 g, 81% yield). It should be noted that substrates **2** with electron-donating groups (Me, MeO) were also tested under the neat condition; only trace products were detected, with most difluoroenoxysilanes recovered.

**Scheme 3:**
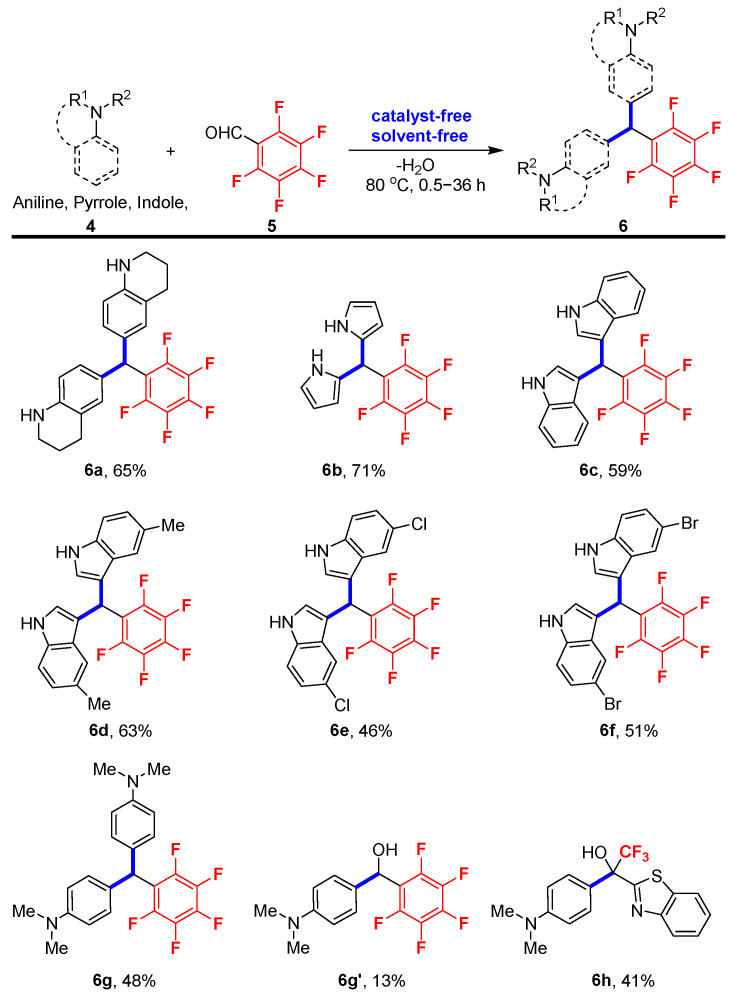
Standard reaction conditions: aniline, pyrrole or indole **4** (1.0 mmol) and pentafluorobenzaldehyde **5** (0.5 mmol) at 80 °C. The yields are isolated yields.

Having developed an efficient procedure for the preparation of *gem*-difluorinated 2-hydroxy-1,4-dicarbonyl compounds, we next turned our attention to investigating the synthesis of polyfluoroarylated compounds with pentafluorobenzaldehyde through the nucleophilic attack pathway. As depicted in Scheme 3, pentafluorobenzene-derived bis(tetrahydroquinolinyl)methane **6a**, bis(pyrrolyl)methanes **6b** and bis(indolyl)methanes **6c**–**6f** were obtained in moderate yields within 0.5–6 h. Meanwhile, for *N*,*N*-dimethylaniline **4g** without free NH moiety in the molecular skeleton, the reaction rate was greatly slowed down. Both the bis(aryl)methane product **6g** (48% yield) and the direct hydroxyarylation product **6g’** (13% yield) were produced despite the reaction time extending to even six times longer than before (36 h). It should be noted that benzothiazole-bearing trifluoromethyl ketone hydrate was also a viable substrate for this neat reaction, although only 41% yield was achieved for product **6h**. And it should be noted that a relatively higher temperature (80 °C) was required for the reaction of pentafluorobenzaldehyde with these different reactants in order to improve the reaction yields (see the Supplementary Materials for complete details of reaction optimization).

**Scheme 4:**
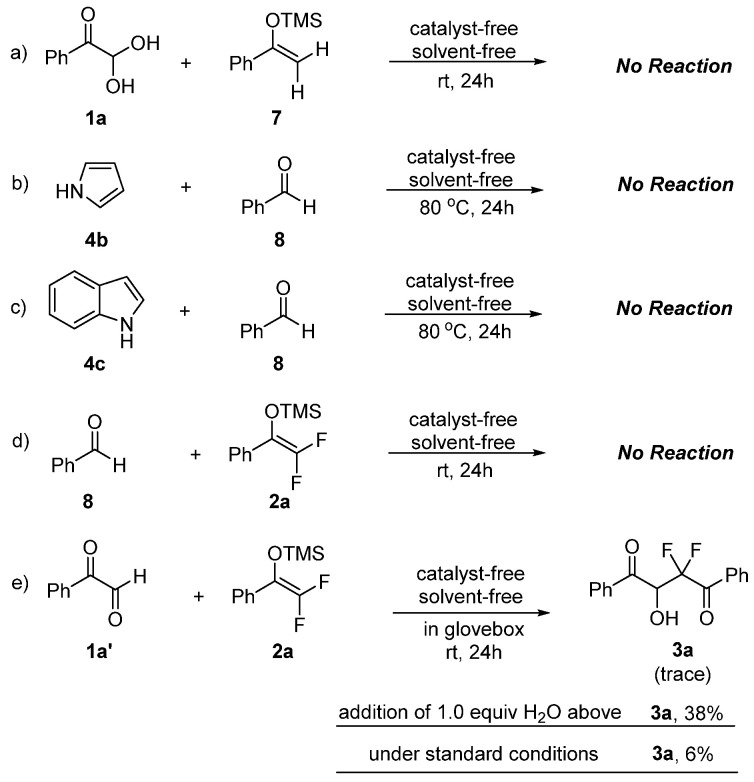
Control experiments.

Several control experiments were carried out to better understand the mechanisms of solvent- and catalyst-free synthesis of *gem*-difluorinated and polyfluoroarylated molecules (Scheme 4). First, the role of the fluorine atom with the reaction partners was investigated with non-fluorinated analogues **7** and **8**, and no reactions occurred after 24 h (Scheme 4a–c). These results proved the indispensable role of the fluorine atom in reactants to smoothly promote this neat reaction, and the installation of fluorine atoms in reactants would have a dramatic impact on reactivity. Next, the reaction with benzaldehyde **8** still could not proceed under the same condition (Scheme 4d). A similar result was obtained with anhydrous phenylglyoxal **1a′**, indicating that the hydrate form of reactants plays a key role in promoting solvent- and catalyst-free synthesis of *gem*-difluorinated compounds with nucleophilic difluoroenoxysilanes (Scheme 4e). Interestingly, it was found that the reaction could proceed smoothly to afford **3a** with the addition of 1.0 equiv. H_2_O, albeit in a lower yield (38%). The reaction conducted under standard conditions without the addition of H_2_O gave **3a** in fairly low yield (6%). Taken together, these preliminary results clearly revealed the important role of the hydrate of glyoxal to promote the neat reaction.

**Scheme 5:**
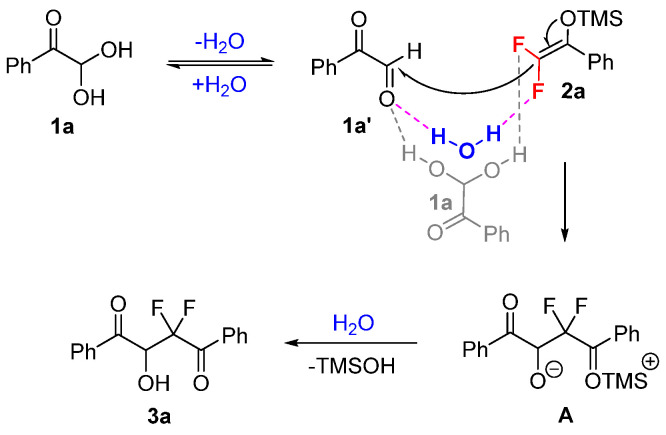
Plausible mechanism.

Based on the current control experiments and previous studies [27,28,29], a possible mechanism for the solvent- and catalyst-free synthesis of *gem*-difluorinated 2-hydroxy-1,4-dicarbonyl products **3** is illustrated in Scheme 5. The glyoxal monohydrates are in equilibrium with their aldehyde form [30], which may drive the occurrence of the neat reaction. Arylglyoxals and difluoroenoxysilanes could be activated through the simultaneous hydrogen-bonding interaction and C–F···H–O interaction between the substrates [31,32]. The water produced from glyoxal monohydrates may help to activate the reaction partners. Then, the additional intermediate **A** could be formed through an intramolecular-like nucleophilic attack of difluoroenoxysilane **2a** on arylglyoxal **1a′**. Finally, the protonation of the generated intermediate **A** would provide the desired *gem*-difluorinated product **3a** smoothly. The synthesis of polyfluoroarylated compounds with pentafluorobenzaldehyde may be promoted through the C–F···H–N interaction between fluorinated and non-fluorinated reactants in a similar pathway.

## 3. Materials and Methods

### 3.1. Materials

All reagents were obtained from commercial sources and used directly without further purification unless otherwise noted. ^1^H, ^13^C and ^19^F were recorded on a Bruker AV 400 MHz instrument at 400 MHz (^1^H NMR) and 100 MHz (^13^C NMR), as well as 376 MHz (^19^F NMR). Chemical shifts were reported in ppm downfield from internal Me_4_Si and external CCl_3_F, respectively. 

### 3.2. General Procedure for the Synthesis of gem-Difluorinated 2-Hydroxy-1,4-dicarbonyl Products

To a 5.0 mL vial was added glyoxal monohydrates **1** (1.0 mmol) and difluoroenoxysilanes **2** (1.0 mmol, 1.0 equiv.). The resulting mixture was stirred at room temperature until the completion of the reaction (monitored by TLC, approximately 2–6 h). Then, water (1.0 mL) was added to the liquefied mixture, followed by vigorous stirring for about 0.5 hour until precipitate was generated. The precipitate was directly filtered, washed with saturated sodium bicarbonate (3 × 2.0 mL) and dried under vacuum to afford the desired pure products **3**.

### 3.3. General Procedure for the Synthesis of Polyfluoroarylated Compounds

To a 5.0 mL oven-dried Schlenk flask was added aniline, pyrrole or indole **4** (1.0 mmol) and pentafluorobenzaldehyde **5** (0.5 mmol) under an argon atmosphere. The resulting mixture was stirred at 80 °C until the completion of the reaction (monitored by TLC, approximately 0.5–36 h). Then, the reaction mixture was separated by column chromatography (petroleum ether/ethyl acetate) to produce the pure polyfluoroarylated products.

## 4. Conclusions

In summary, we have successfully developed a facile and environmentally friendly method for the synthesis of a series of *gem*-difluorinated and polyfluoroarylated compounds using nucleophilic difluoroenoxysilane and electrophilic pentafluorobenzaldehyde under neat conditions. Readily available reactants, a broad substrate scope, solvent- and catalyst-free conditions and a simple work-up procedure without column chromatographic separation are the prominent features of this green and concise protocol. 

## Figures and Tables

**Table 1:** Optimization of the reaction conditions *^a^*.

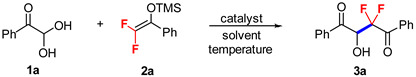			
**Entry**	**Solvent**	**Time (h)**	**Yield (%) *^b^***
1	−	4	91
2 *^c^*	−	4	89
3 *^d^*	−	2	64
4 *^e^*	toluene	36	9
5 *^e^*	DCM	36	6
6 *^e^*	THF	36	trace
7 *^e^*	MeOH	36	23
8 *^e^*	EtOH	36	16
9 *^e^*	H_2_O	36	19
10 *^f^*	DCM	36	42
11 *^g^*	DCM	36	28
12 *^h^*	DCM	36	13

*^a^* Reaction conditions: glyoxal monohydrate **1a** (1.0 mmol), difluoroenoxysilane **2a** (2.0 mmol, 2.0 equiv) at room temperature unless otherwise noted; *^b^* isolated yield; *^c^* 1.0 equiv. **2a** was used; *^d^* reaction was performed at 60 °C; *^e^* 1.0 mL solvent was used; *^f^* 0.1 mmol Sc(OTf)_3_ was used as catalyst in 10.0 mL DCM; *^g^* 0.1 mmol PhB(OH)_2_ was used as catalyst in 10.0 mL DCM; *^h^* 0.1 mmol PTSA was used as catalyst in 10.0 mL DCM.

## Data Availability

Data are contained within the article and Supplementary Materials.

## Supplementary Materials

The following supporting information can be downloaded at: https://www.mdpi.com/article/10.3390/molecules29030697/s1, S2. General information, S2–S9. General procedure for the synthesis of gem-difluorinated 2-hydroxy-1,4-dicarbonyl products, S9. Optimization of the reaction conditions for the synthesis of polyfluoroarylated compounds, S10–S14. General procedure for the synthesis of polyfluoroarylated compounds, S14–S59. NMR spectra of the related compounds, [33,34,35,36].
