# Supplementary material for: Solvent- and Catalyst-Free Synthesis of gem-Difluorinated and Polyfluoroarylated Compounds with Nucleophilic or Electrophilic Fluorine-Containing Reaction Partners, Respectively
Source: Molecules. 2024 Feb 2;29(3):697. doi: 10.3390/molecules29030697 (PMC10856203; doi:10.3390/molecules29030697)

Supporting Information (SI)

Solvent- and catalyst-free synthesis of gem-difluorinated and polyfluoroarylated compounds with nucleophilic or electrophilic fluorine-containing reaction partners respectively

Lingheng Li [1] and Jinshan Li [2,*]

[1]*Department of Photography, Tianjin University of Technology, Tianjin, 300384, China*

[2] *School of Chemistry and Chemical Engineering, Hainan University, Haikou, 570228, China*

**E-mail:* lijinshan@hainanu.edu.cn

Contents

General information…………………………..………………..…………………S2

General procedure for the synthesis of *gem*-difluorinated 2-hydroxy-1,4-dicarbonyl products……..S2-S9

Optimization of the reaction conditions for the synthesis of polyfluoroarylated compounds……...S9

General procedure for the synthesis of polyfluoroarylated compounds…………………………..…………...S10-S14

Reference…………………………………………………………………………...S14

NMR spectra of the related compounds……………………………………......S15-S59

General information

^{1}H, ^{13}C and ^{19}F were recorded on Bruker AV 400 MHz instrument at 400 MHz (^{1}H NMR), 100 MHz (^{13}C NMR), as well as 376 MHz (^{19}F NMR). Chemical shifts were reported in ppm down field from internal Me_4Si and external CCl_3F, respectively. Data for ^{1}H were reported as follows: chemical shift (ppm), multiplicity (s = singlet, d = doublet, t = triplet, q = quartet, dd = doublet of doublets, m = multiplet, br = broad singlet), coupling constants (Hz), and integration. Data for ^{13}C NMR were reported as ppm. High-resolution mass spectra analyses were performed on a Waters SYNAPT G2-Si Q-TOF mass spectrometer. Melting points were determined using a X-4 digital micro melting point apparatus. Thin-layer chromatography (TLC) was performed, and visualization of the compounds was accomplished with UV light (254 nm).

Materials: Unless otherwise indicated, all reactions were carried out in air. All solvents were distilled from appropriate drying agents prior to use. All purchased reagents were used without further purification. Analytical thin layer chromatography was performed on 0.20 mm Qingdao Haiyang silica gel plates. Glyoxal monohydrates were prepared according to literature procedures.[1] Difluoroenoxysilanes[2] were prepared according to the reported procedures.

General procedure for the synthesis of *gem*-difluorinated 2-hydroxy-1,4-dicarbonyl products

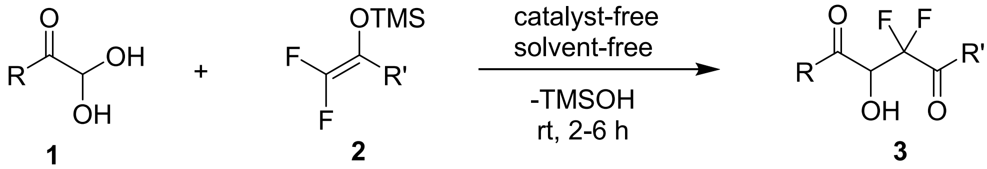

To a 5.0 mL vial was added glyoxal monohydrates **1** (1.0 mmol) and difluoroenoxysilanes **2** (1.0 mmol, 1.0 equiv). The resulting mixture was stirred at room temperature until the completion of the reaction (monitored by TLC, approximately 2–6 hours). Then water (1.0 mL) was added to the liquefied mixture and followed by vigorously stirring for about 0.5 hour until precipitate was generated. The precipitate was directly filtered, washed with saturated sodium bicarbonate (3 x 2.0 mL), and dried under vacuum to afford the desired products **3**.

A detailed operation is shown in the following figure:

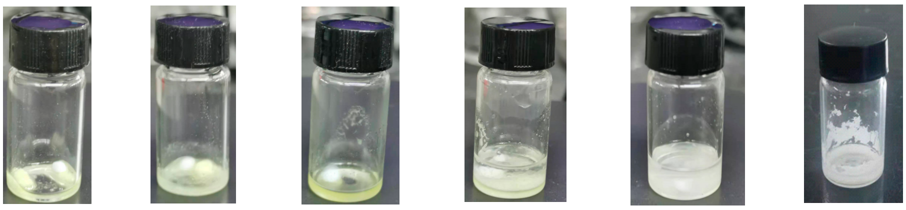

(a) (b) (c) (d) (e) (f)

(a) After the addition of glyoxal monohydrates and difluoroenoxysilanes;
(b) During the stirring process;
(c) The reaction is completed;
(d) Water is added to the reaction mixture;
(e) The reaction mixture is vigorously stirred at room temperature to generate the precipitate;
(f) Filtration and drying to obtain the final product.

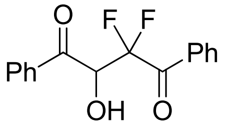

3a

2,2-difluoro-3-hydroxy-1,4-diphenylbutane-1,4-dione (3a): white solid, 258.2 mg, 89% yield. M.p.: 62–64 ºC; **^{1}H NMR** (400 MHz, $CDCl_3$) *δ* 8.05 (t, *J* = 8.0 Hz, 4H), 7.65 (dt, *J* = 14.7, 7.4 Hz, 2H), 7.51 (dt, *J* = 18.3, 7.8 Hz, 4H), 5.73 (ddd, *J* = 17.8, 7.5, 4.0 Hz, 1H), 4.26 (d, *J* = 7.6 Hz, 1H); **^{19}F NMR** (376 MHz, $CDCl_3$) *δ* −100.63 (d, *J* = 279.8 Hz, 1F), −113.10 (dd, *J* = 278.2, 17.9 Hz, 1F); **^{13}C{^{1}H} NMR** (100 MHz, $CDCl_3$) *δ* 195.1, 189.3 (C−F, dd, $^2J_{C-F}$ = 30.0, 27.0 Hz), 134.7, 134.4, 132.6 (C−F, d, $^3J_{C-F}$ = 1.6 Hz), 130.1 (C−F, dd, $^3J_{C-F}$ = 4.1, 2.7 Hz), 129.5 (C−F, d, $^3J_{C-F}$ = 1.5 Hz), 128.7, 128.6, 115.9 (C−F, dd, $^1J_{C-F}$ = 266.1, 261.7 Hz), 72.3 (C−F, t, $^2J_{C-F}$ = 27.0 Hz). **HRMS** (ESI) m/z: $[M + Na]^+$ Calcd for $C_{16}H_{12}F_2O_3Na$ 313.0652; Found 313.0658.

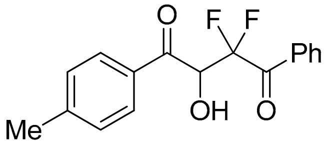

3b

2,2-difluoro-3-hydroxy-1-phenyl-4-(*p*-tolyl)butane-1,4-dione (3b): white solid, 255.3 mg, 84% yield. M.p.: 70–71 ºC; **^{1}H NMR** (400 MHz, $CDCl_3$) δ 7.83 (dd, *J* = 45.9, 7.6 Hz, 4H), 7.46 (t, *J* = 7.4 Hz, 1H), 7.31 (t, *J* = 7.8 Hz, 2H), 7.16 (d, *J* = 8.0 Hz, 2H), 5.53 (ddd, *J* = 18.3, 7.4, 3.7 Hz, 1H), 4.12 (d, *J* = 7.6 Hz, 1H), 2.27 (s, 3H); **^{19}F NMR** (376 MHz, $CDCl_3$) δ −100.57 (d, *J* = 276.4 Hz, 1F), −113.71 (dd, *J* = 276.7, 18.8 Hz, 1F); **^{13}C{^{1}H} NMR** (100 MHz, $CDCl_3$) δ 194.4, 189.4 (C−F, dd, $^2J_{C-F}$ = 29.9, 26.7 Hz), 146.1, 134.4, 132.7, 131.9, 130.1 (C−F, dd, $^3J_{C-F}$ = 4.1, 2.5 Hz), 129.6, 129.5, 128.6, 115.9 (C−F, dd, $^1J_{C-F}$ = 266.1, 261.2 Hz), 72.1 (C−F, t, $^2J_{C-F}$ = 27.1 Hz), 21.8; **HRMS** (ESI) m/z: $[M + Na]^+$ Calcd for $C_{17}H_{14}F_2O_3Na$ 327.0809; Found 327.0816.

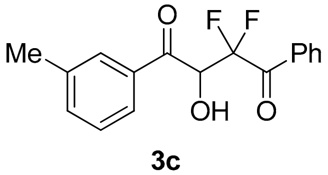

3c

2,2-difluoro-3-hydroxy-1-phenyl-4-(*m*-tolyl)butane-1,4-dione (3c): white solid, 246.4 mg, 81% yield. M.p.: 74–75 ºC; **^{1}H NMR** (400 MHz, $CDCl_3$) δ 8.05 (d, *J* = 7.6 Hz, 2H), 7.82 (d, *J* = 10.1 Hz, 2H), 7.63 (t, *J* = 7.4 Hz, 1H), 7.49 (t, *J* = 7.8 Hz, 3H), 7.41 (t, *J* = 7.6 Hz, 1H), 5.71 (ddd, *J* = 17.8, 7.6, 4.1 Hz, 1H), 4.25 (d, *J* = 7.6 Hz, 1H), 2.44 (s, 3H); **^{19}F NMR** (376 MHz, $CDCl_3$) δ −100.65 (d, *J* = 277.6 Hz, 1F), −113.17 (dd, *J* = 277.5, 17.5 Hz, 1F); **^{13}C{^{1}H} NMR** (100 MHz, $CDCl_3$) δ 195.2, 189.3 (C−F, dd, $^2J_{C-F}$ = 30.0, 26.9 Hz), 138.7, 135.6, 134.5, 134.4, 132.7 (C−F, d, $^3J_{C-F}$ = 1.9 Hz), 130.1 (C−F, dd, $^3J_{C-F}$ = 4.1, 2.7 Hz), 129.8 (C−F, d, $^3J_{C-F}$ = 1.0 Hz), 128.6, 128.6, 126.8 (C−F, d, $^3J_{C-F}$ = 1.6 Hz), 115.9 (C−F, dd, $^1J_{C-F}$ = 266.1, 261.7 Hz), 72.3 (C−F, t, $^2J_{C-F}$ = 26.9 Hz), 21.3; **HRMS** (ESI) m/z: $[M + Na]^+$ Calcd for $C_{17}H_{14}F_2O_3Na$ 327.0809; Found 327.0817.

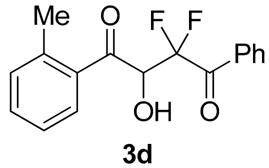

3d

2,2-difluoro-3-hydroxy-1-phenyl-4-(*o*-tolyl)butane-1,4-dione (3d): white solid, 212.8 mg, 70% yield. M.p.: 59–61 ºC; **^{1}H NMR** (400 MHz, $CDCl_3$) δ 7.85 (d, J = 7.7 Hz, 2H), 7.46 (dd, J = 16.7, 7.7 Hz, 2H), 7.30 (t, J = 7.8 Hz, 3H), 7.13 (dd, J = 19.2, 11.0 Hz, 2H), 5.44 (dt, J = 16.3, 6.1 Hz, 1H), 4.15 (d, J = 6.7 Hz, 1H), 2.39 (s, 3H); **^{19}F NMR** (376 MHz, $CDCl_3$) δ −101.09 (dd, J = 279.7, 5.3 Hz, 1F), −110.60 (dd, J = 279.4, 16.5 Hz, 1F); **^{13}C{^{1}H} NMR** (100 MHz, $CDCl_3$) δ 197.4 , 189.1 (C−F, dd, $^2J_{C-F}$ = 30.0, 26.9 Hz), 139.6, 134.6, 134.4, 132.9, 132.6 (C−F, t, $^3J_{C-F}$ = 1.6 Hz), 130.0 (C−F, t, $^3J_{C-F}$ = 3.3 Hz), 129.7, 128.6, 125.6, 116.1 (C−F, dd, $^1J_{C-F}$ = 264.0, 263.1 Hz), 73.6 (C−F, t, $^2J_{C-F}$ = 26.3 Hz), 20.8; **HRMS** (ESI) m/z: $[M + Na]^+$ Calcd for $C_{17}H_{14}F_2O_3Na$ 327.0809; Found 327.0815.

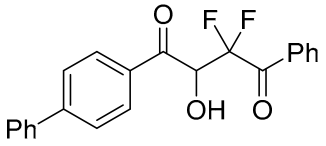

3e

4-([1,1'-biphenyl]-4-yl)-2,2-difluoro-3-hydroxy-1-phenylbutane-1,4-dione (3e): pale yellow solid, 260.1 mg, 71% yield. M.p.: 79–81 ºC; **^{1}H NMR** (400 MHz, $CDCl_3$) δ 8.09 (dd, J = 16.7, 7.8 Hz, 4H), 7.75 (d, J = 8.5 Hz, 2H), 7.68 – 7.59 (m, 3H), 7.53 – 7.45 (m, 4H), 7.45 – 7.39 (m, 1H), 5.75 (ddd, J = 17.9, 7.5, 3.9 Hz, 1H), 4.29 (d, J = 7.6 Hz, 1H); **^{19}F NMR** (376 MHz, $CDCl_3$) δ −100.56 (d, J = 276.7 Hz, 1F), −113.21 (dd, J = 277.5, 17.7 Hz, 1F); **^{13}C{^{1}H} NMR** (100 MHz, $CDCl_3$) δ 194.5, 189.4 (C−F, dd, $^2J_{C-F}$ = 29.9, 26.9 Hz), 147.5, 139.4, 134.4, 133.0, 132.7 (C−F, d, $^3J_{C-F}$, J = 1.7 Hz), 130.2 (C−F, d, $^3J_{C-F}$, J = 2.9 Hz), 130.1, 129.0, 128.6 (C−F, d, $^3J_{C-F}$ = 3.0 Hz), 127.3 (C−F, d, $^3J_{C-F}$ = 3.6 Hz), 116.0 (C−F, dd, $^1J_{C-F}$ = 266.3, 261.6 Hz), 72.3 (C−F, dd, $^2J_{C-F}$ = 27.5, 26.5 Hz); **HRMS** (ESI) m/z: $[M + Na]^+$ Calcd for $C_{22}H_{16}F_2O_3Na$ 389.0965; Found 389.0970.

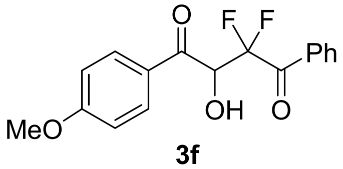

3f

2,2-difluoro-3-hydroxy-4-(4-methoxyphenyl)-1-phenylbutane-1,4-dione (3f): white solid, 265.8 mg, 83% yield. M.p.: 84–86 ºC; **^{1}H NMR** (400 MHz, $CDCl_3$) δ 8.05 (t, J = 8.1 Hz, 4H), 7.67 – 7.58 (m, 1H), 7.48 (t, J = 7.8 Hz, 2H), 7.04 – 6.94 (m, 2H), 5.67 (ddd, J = 18.7, 7.6, 3.5 Hz, 1H), 4.32 (d, J = 7.7 Hz, 1H), 3.89 (s, 3H); **^{19}F NMR** (376 MHz, $CDCl_3$) δ −100.59 (d, J = 274.6 Hz, 1F), −114.41 (dd, J = 274.7, 19.1 Hz, 1F); **^{13}C{^{1}H} NMR** (100 MHz, $CDCl_3$) δ 192.8, 189.5 (C−F, dd, $^2J_{C-F}$ = 30.1, 26.4 Hz), 164.9, 134.3, 132.8, 132.0 (C−F, d, $^2J_{C-F}$ = 2.1 Hz), 130.1 (C−F, dd, $^3J_{C-F}$ = 4.3, 2.5 Hz), 128.6, 127.2, 116.0 (C−F, dd, $^1J_{C-F}$ = 266.4, 260.8 Hz), 114.1, 71.85 (C−F, dd, $^2J_{C-F}$ = 28.2, 26.3 Hz), 55.6; **HRMS** (ESI) m/z: $[M + Na]^+$ Calcd for $C_{17}H_{14}F_2O_4Na$ 343.0758; Found 343.0765.

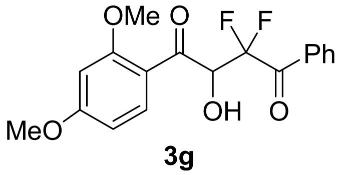

3g

4-(2,4-dimethoxyphenyl)-2,2-difluoro-3-hydroxy-1-phenylbutane-1,4-dione (3g): white solid, 238.2 mg, 68% yield. M.p.: 107–108 ºC; **^{1}H NMR** (400 MHz, $CDCl_3$) δ 8.05 (d, J = 7.5 Hz, 2H),

7.87 (d, J = 8.8 Hz, 1H), 7.60 (d, J = 7.4 Hz, 1H), 7.47 (t, J = 7.8 Hz, 2H), 6.58 (dd, J = 8.8, 2.3 Hz, 1H), 6.45 (d, J = 2.2 Hz, 1H), 6.03 (ddd, J = 17.8, 7.4, 5.1 Hz, 1H), 4.37 (d, J = 7.5 Hz, 1H), 3.88 (d, J = 7.4 Hz, 6H); **^{19}F NMR** (376 MHz, $CDCl_3$) δ −103.96 − −104.70 (m, 1F), −114.17 (dd, J = 275.5, 18.0 Hz, 1F); **^{13}C{^{1}H} NMR** (100 MHz, $CDCl_3$) δ 193.7, 189.2 (C−F, dd, $^{2}J_{C-F}$ = 29.6, 27.5 Hz), 166.0, 161.7, 134.1, 133.6, 132.8, 130.0 (C−F, dd, $^{3}J_{C-F}$ = 3.4 Hz), 128.5, 118.2, 116.4 (C−F, dd, $^{1}J_{C-F}$ = 264.1, 262.1 Hz), 106.0, 98.4, 74.9 (C−F, t, $^{2}J_{C-F}$ = 25.8, 24.7 Hz), 55.6, 55.5; **HRMS** (ESI) m/z: $[M + Na]^+$ Calcd for $C_{18}H_{16}F_2O_5Na$ 373.0863; Found 373.0868.

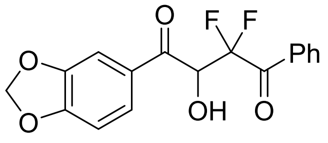

3h

4-(benzo[*d*][1,3]dioxol-5-yl)-2,2-difluoro-3-hydroxy-1-phenylbutane-1,4-dione (3h): pale yellow solid, 257.3 mg, 77% yield. M.p.: 125–127 °C; **^{1}H NMR** (400 MHz, $CDCl_3$) δ 8.06 (d, J = 7.7 Hz, 2H), 7.70 – 7.58 (m, 2H), 7.55 – 7.42 (m, 3H), 6.92 (d, J = 8.2 Hz, 1H), 6.09 (s, 2H), 5.63 (ddd, J = 18.3, 7.7, 3.6 Hz, 1H), 4.28 (d, J = 7.8 Hz, 1H); **^{19}F NMR** (376 MHz, $CDCl_3$) δ −100.61 (d, J = 275.8 Hz, 1F), −114.14 (dd, J = 275.5, 18.4 Hz, 1F); **^{13}C{^{1}H} NMR** (100 MHz, $CDCl_3$) δ 192.6, 189.4 (C−F, dd, $^{2}J_{C-F}$ = 30.1, 26.5 Hz), 153.4, 148.4, 134.4, 132.8 (C−F, d, $^{3}J_{C-F}$ = 2.0 Hz), 130.1 (C−F, dd, $^{3}J_{C-F}$ = 4.4, 2.4 Hz), 128.9, 128.6, 126.8 (C−F, d, $^{3}J_{C-F}$ = 2.9 Hz), 115.9 (C−F, dd, $^{1}J_{C-F}$ = 266.4, 261.1 Hz), 108.8, 108.1, 102.2, 72.0 (C−F, dd, $^{2}J_{C-F}$ = 27.8, 26.3 Hz); **HRMS** (ESI) m/z: $[M + Na]^+$ Calcd for $C_{17}H_{12}F_2O_5Na$ 357.0550; Found 357.0556.

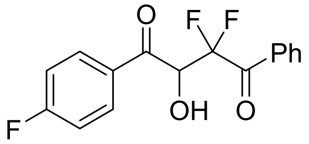

3i

2,2-difluoro-4-(4-fluorophenyl)-3-hydroxy-1-phenylbutane-1,4-dione (3i): white solid, 289.6 mg, 94% yield. M.p.: 87–88 °C; **^{1}H NMR** (400 MHz, $CDCl_3$) δ 8.17 – 7.99 (m, 4H), 7.64 (t, J = 7.4 Hz, 1H), 7.49 (t, J = 7.8 Hz, 2H), 7.21 (t, J = 8.6 Hz, 2H), 5.68 (ddd, J = 17.5, 7.6, 4.0 Hz, 1H), 4.24 (d, J = 7.7 Hz, 1H); **^{19}F NMR** (376 MHz, $CDCl_3$) δ −100.79 (d, J = 277.4 Hz, 1F), −101.55 − −101.62 (m, 1F), −112.99 (dd, J = 279.1, 17.3 Hz, 1F); **^{13}C{^{1}H} NMR** (100 MHz, $CDCl_3$) δ 193.5, 189.3 (C−F, dd, $^{2}J_{C-F}$ = 29.7, 26.8 Hz), 166.7 (C−F, d, $^{1}J_{C-F}$ = 258.0 Hz), 134.5, 132.6, 132.3 (C−F, d, $^{3}J_{C-F}$ = 9.6 Hz), 130.8 (C−F, d, $^{3}J_{C-F}$ = 2.1 Hz), 130.1 (C−F, dd, $^{3}J_{C-F}$ = 3.9, 2.7 Hz), 128.7, 116.1 (C−F, d, $^{2}J_{C-F}$ = 22.2 Hz), 115.8 (C−F, dd, $^{1}J_{C-F}$ = 266.2, 261.4 Hz), 72.3 (C−F, t, $^{2}J_{C-F}$ = 26.9 Hz); **HRMS** (ESI) m/z: $[M + Na]^+$ Calcd for $C_{16}H_{12}F_3O_3Na$ 331.0571; Found 331.0563.

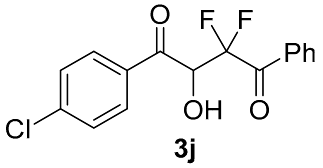

3j

4-(4-chlorophenyl)-2,2-difluoro-3-hydroxy-1-phenylbutane-1,4-dione (3j): white solid, 298.4 mg, 92% yield. M.p.: 94–95 °C; **^{1}H NMR** (400 MHz, $CDCl_3$) δ 8.01 (dd, J = 28.0, 7.8 Hz, 4H), 7.73 – 7.59 (m, 1H), 7.59 – 7.42 (m, 4H), 5.67 (ddd, J = 17.2, 7.6, 4.1 Hz, 1H). 4.20 (d, J = 7.7 Hz,

1H); **^{19}F NMR** (376 MHz, $CDCl_3$) δ −100.81 (d, J = 279.8 Hz, 1F), −112.55 (dd, J = 279.6, 17.1 Hz, 1F); **^{13}C{^{1}H} NMR** (100 MHz, $CDCl_3$) δ 194.0, 189.2 (C−F, dd, $^2J_{C-F}$ = 29.7, 26.9 Hz), 141.4, 134.5, 132.7, 132.5 (C−F, t, $^3J_{C-F}$ = 1.7 Hz), 130.8 (C−F, d, $^3J_{C-F}$ = 1.1 Hz), 130.1 (C−F, dd, $^3J_{C-F}$ = 4.0, 2.8 Hz), 129.2, 128.7, 115.8 (C−F, dd, $^1J_{C-F}$ = 266.2, 261.8 Hz), 72.5 (C−F, t, $^2J_{C-F}$ = 26.9 Hz); **HRMS** (ESI) m/z: $[M + Na]^+$ Calcd for $C_{16}H_{12}ClF_2O_3Na$ 347.0262; Found 347.0267.

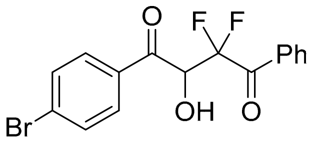

3k

4-(4-bromophenyl)-2,2-difluoro-3-hydroxy-1-phenylbutane-1,4-dione (3k): yellow solid, 306.4 mg, 83% yield. M.p.: 71–73 °C; **^{1}H NMR** (400 MHz, $CDCl_3$) δ 8.05 (d, J = 7.6 Hz, 2H), 7.89 (d, J = 8.3 Hz, 2H), 7.65 (dd, J = 17.9, 8.0 Hz, 3H), 7.49 (t, J = 7.8 Hz, 2H), 5.66 (ddd, J = 17.1, 7.5, 4.2 Hz, 1H), 4.22 (d, J = 7.6 Hz, 1H); **^{19}F NMR** (376 MHz, $CDCl_3$) δ −100.84 (d, J = 279.7 Hz, 1F), −112.43 (dd, J = 279.7, 17.0 Hz, 1F); **^{13}C{^{1}H} NMR** (100 MHz, $CDCl_3$) δ 194.3, 189.2 (C−F, dd, $^2J_{C-F}$ = 29.6, 27.1 Hz), 134.5, 133.2, 132.5 (C−F, t, $^3J_{C-F}$ = 2.0 Hz), 132.2, 130.8 (C−F, t, $^3J_{C-F}$ = 1.1 Hz), 130.2, 130.1 (C−F, dd, $^3J_{C-F}$ = 4.0, 2.8 Hz), 128.7, 115.8 (C−F, dd, $^1J_{C-F}$ = 266.2, 261.9 Hz), 72.5 (C−F, t, $^2J_{C-F}$ = 26.9 Hz); **HRMS** (ESI) m/z: $[M + Na]^+$ Calcd for $C_{16}H_{11}BrF_2O_3Na$ 390.9757; Found 390.9764.

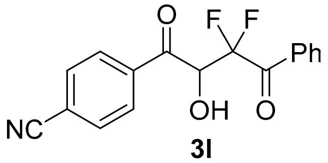

3l

4-(3,3-difluoro-2-hydroxy-4-oxo-4-phenylbutanoyl)benzonitrile (3l): white solid, 293.2 mg, 93% yield. M.p.: 104–105 °C; **^{1}H NMR** (400 MHz, $CDCl_3$) δ 8.08 (dd, J = 28.3, 7.9 Hz, 4H), 7.83 (d, J = 8.4 Hz, 2H), 7.66 (t, J = 7.4 Hz, 1H), 7.50 (t, J = 7.8 Hz, 2H), 5.67 (dd, J = 15.4, 4.4 Hz, 1H), 4.16 (s, 1H); **^{19}F NMR** (376 MHz, $CDCl_3$) δ −101.37 (dd, J = 285.1, 3.9 Hz), -110.31 (dd, J = 283.9, 15.5 Hz); **^{13}C{^{1}H} NMR** (100 MHz, $CDCl_3$) δ 194.5 (C−F, d, $^3J_{C-F}$ = 1.6 Hz), 189.1 (C−F, dd, $^2J_{C-F}$ = 29.2, 27.8 Hz), 137.6, 134.8, 132.5, 132.2 (C−F, t, $^3J_{C-F}$ = 2.0 Hz), 130.1 (C−F, t, $^3J_{C-F}$ = 3.4 Hz), 129.7, 128.7, 117.6 (C−F, d, $^3J_{C-F}$ = 10.7 Hz), 115.8 (C−F, dd, $^1J_{C-F}$ = 265.4, 263.1 Hz), 73.1 (C−F, t, $^2J_{C-F}$ = 26.8 Hz); **HRMS** (ESI) m/z: $[M + Na]^+$ Calcd for $C_{17}H_{11}F_2NO_3Na$ 338.0605; Found 338.0611.

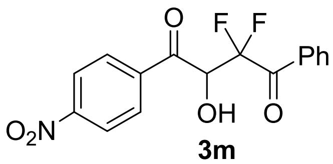

3m

2,2-difluoro-3-hydroxy-4-(4-nitrophenyl)-1-phenylbutane-1,4-dione (3m): yellow solid, 321.8 mg, 96% yield. M.p.: 95–96 °C; **^{1}H NMR** (400 MHz, $CDCl_3$) δ 8.37 (d, J = 8.7 Hz, 2H), 8.19 (d, J = 8.6 Hz, 2H), 8.07 (d, J = 14.1 Hz, 2H), 7.66 (t, J = 7.4 Hz, 1H), 7.50 (t, J = 7.8 Hz, 2H), 5.69 (dd, J = 15.0, 4.3 Hz, 1H), 4.16 (s, 1H); **^{19}F NMR** (376 MHz, $CDCl_3$) δ −100.98 − − 101.75 (m, 1F), −109.81 (dd, J = 284.5, 15.2 Hz, 1F); **^{13}C{^{1}H} NMR** (100 MHz, $CDCl_3$) δ 194.4 (C−F, d, $^2J_{C-F}$ = 1.7 Hz), 189.0 (C−F, t, $^2J_{C-F}$ = 28.4 Hz), 150.9, 139.1, 134.8, 133.7, 132.17 (C−F, d, $^2J_{C-F}$

= 2.2 Hz), 130.4, 130.1 (C−F, t, ${}^{3}J_{C-F}$ = 3.3 Hz), 128.8, 128.5, 123.9, 115.8 (C−F, dd, ${}^{1}J_{C-F}$ = 265.0, 263.6 Hz), 73.3 (C−F, t, ${}^{2}J_{C-F}$ = 26.7 Hz); **HRMS** (ESI) m/z: $[M + Na]^+$ Calcd for $C_{16}H_{11}F_2NO_5Na$ 357.0527; Found 357.0533.

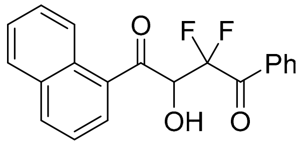

3n

2,2-difluoro-3-hydroxy-4-(naphthalen-1-yl)-1-phenylbutane-1,4-dione (3n): pale yellow solid, 255.2 mg, 75% yield. M.p.: 63–64 ºC; **^{1}H NMR** (400 MHz, $CDCl_3$) δ 8.62 (d, J = 8.5 Hz, 1H), 8.08 (d, J = 8.3 Hz, 1H), 8.00 (d, J = 8.4 Hz, 2H), 7.92 (t, J = 8.6 Hz, 2H), 7.68 (ddd, J = 8.5, 6.9, 1.4 Hz, 1H), 7.63 – 7.57 (m, 2H), 7.57 – 7.52 (m, 1H), 7.45 (t, J = 7.8 Hz, 2H), 5.78 (dt, J = 15.9, 6.2 Hz, 1H), 4.46 (d, J = 6.7 Hz, 1H); **^{19}F NMR** (376 MHz, $CDCl_3$) δ −100.77 (dd, J = 280.0, 5.2 Hz, 1F), −109.90 (dd, J = 280.7, 16.0 Hz, 1F); **^{13}C{^{1}H} NMR** (100 MHz, $CDCl_3$) δ 197.1, 189.1 (C−F, t, ${}^{2}J_{C-F}$ = 28.5 Hz), 134.5, 134.4, 133.9, 132.5 (C−F, t, ${}^{3}J_{C-F}$ = 2.0 Hz), 132.5, 130.3, 130.0 (C−F, t, ${}^{3}J_{C-F}$ = 3.4 Hz), 129.7 (C−F, d, ${}^{3}J_{C-F}$ = 1.9 Hz), 128.7, 128.6, 128.5, 127.0, 125.4, 124.0, 116.2 (C−F, t, ${}^{1}J_{C-F}$ = 263.9 Hz), 73.9 (C−F, t, ${}^{2}J_{C-F}$ = 26.2 Hz); **HRMS** (ESI) m/z: $[M + Na]^+$ Calcd for calcd for $C_{20}H_{14}F_2O_3Na$ 363.0809; Found 363.0815.

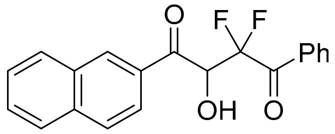

3o

2,2-difluoro-3-hydroxy-4-(naphthalen-2-yl)-1-phenylbutane-1,4-dione (3o): pale yellow solid, 265.4 mg, 78% yield. M.p.: 92–93 ºC; **^{1}H NMR** (400 MHz, $CDCl_3$) δ 8.55 (s, 1H), 8.13 – 7.84 (m, 6H), 7.61 (dd, J = 17.2, 8.9 Hz, 3H), 7.48 (t, J = 7.8 Hz, 2H), 5.89 (ddd, J = 17.8, 7.6, 4.0 Hz, 1H), 4.35 (d, J = 7.7 Hz, 1H); **^{19}F NMR** (376 MHz, $CDCl_3$) δ −100.53 (d, J = 277.7 Hz, 1F), −113.09 (dd, J = 277.8, 17.8 Hz, 1F); **^{13}C{^{1}H} NMR** (100 MHz, $CDCl_3$) δ 194.9, 189.5 (C−F, dd, ${}^{2}J_{C-F}$ = 30.0, 26.7 Hz), 136.3, 134.4, 132.7 (C−F, d, ${}^{3}J_{C-F}$ = 1.7 Hz), 132.2, 132.1, 132.1, 131.7, 130.1 (C−F, dd, ${}^{3}J_{C-F}$ = 4.1, 2.6 Hz), 129.9, 129.4, 128.7, 128.6, 127.8, 127.2, 124.2, 116.0 (C−F, dd, ${}^{1}J_{C-F}$ = 266.2, 261.6 Hz), 72.4 (C−F, t, ${}^{2}J_{C-F}$ = 27.0 Hz); **HRMS** (ESI) m/z: $[M + Na]^+$ Calcd for $C_{20}H_{14}F_2O_3Na$ 363.0809; Found 363.0814.

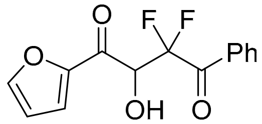

3p

2,2-difluoro-4-(furan-2-yl)-3-hydroxy-1-phenylbutane-1,4-dione (3p): white solid, 179.2 mg, 64% yield. M.p.: 83–85 ºC; **^{1}H NMR** (400 MHz, $CDCl_3$) δ 8.08 (d, J = 7.6 Hz, 2H), 7.82 – 7.69 (m, 1H), 7.65 (t, J = 7.4 Hz, 1H), 7.50 (dd, J = 9.1, 6.1 Hz, 3H), 6.66 (dd, J = 3.6, 1.6 Hz, 1H), 5.51 (ddd, J = 18.3, 7.9, 3.9 Hz, 1H), 4.07 (d, J = 8.1 Hz, 1H); **^{19}F NMR** (376 MHz, $CDCl_3$) δ −102.44 (d, J = 279.3 Hz, 1F), −114.62 (dd, J = 278.3, 18.2 Hz, 1F); **^{13}C{^{1}H} NMR** (100 MHz, $CDCl_3$) δ 188.9 (C−F, dd, ${}^{2}J_{C-F}$ = 29.8, 26.6 Hz), 182.1, 150.4, 148.6, 134.5, 132.5 (C−F, t, ${}^{3}J_{C-F}$ = 1.6 Hz), 130.1 (C−F, dd, ${}^{3}J_{C-F}$ = 4.0, 2.8 Hz), 128.7, 121.5 (C−F, d, ${}^{3}J_{C-F}$ = 1.0 Hz), 115.8 (C−F,

dd, $^{1}J_{C-F}$ = 266.0, 261.3 Hz), 113.1, 72.5 (C−F, dd, $^{2}J_{C-F}$ = 28.8, 25.8 Hz); **HRMS** (ESI) m/z: [M + Na]$^+$ Calcd for $C_{14}H_{10}F_2O_4Na$ 303.0445; Found 303.0449.

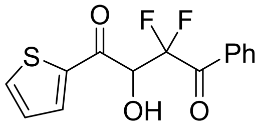

3q

2,2-difluoro-3-hydroxy-1-phenyl-4-(thiophen-2-yl)butane-1,4-dione (3q): pale yellow solid, 159.7 mg, 54% yield. M.p.: 66–68 ºC; **^{1}H NMR** (400 MHz, $CDCl_3$) δ 8.07 (d, *J* = 7.6 Hz, 2H), 7.99 – 7.91 (m, 1H), 7.84 (dd, *J* = 4.9, 0.9 Hz, 1H), 7.64 (t, *J* = 7.4 Hz, 1H), 7.50 (t, *J* = 7.8 Hz, 2H), 7.23 (dd, *J* = 4.8, 4.1 Hz, 1H), 5.52 (ddd, *J* = 18.3, 7.8, 3.9 Hz, 1H), 4.14 (d, *J* = 7.9 Hz, 1H); **^{19}F NMR** (376 MHz, $CDCl_3$) δ −101.36 (d, *J* = 277.3 Hz, 1F), −114.39 (dd, *J* = 277.2, 19.0 Hz, 1F); **^{13}C{^{1}H} NMR** (100 MHz, $CDCl_3$) δ 189.3 (C−F, dd, $^{2}J_{C-F}$ = 30.1, 26.5 Hz), 186.6, 140.5, 136.7, 135.5 (C−F, d, $^{3}J_{C-F}$ = 3.1 Hz), 134.5, 132.6 (C−F, d, $^{3}J_{C-F}$ = 1.7 Hz), 130.1 (C−F, dd, $^{3}J_{C-F}$ = 4.4, 2.4 Hz), 128.7, 115.6 (C−F, dd, $^{1}J_{C-F}$ = 266.6, 260.8 Hz), 73.3 (C−F, dd, $^{2}J_{C-F}$ = 28.8, 26.2 Hz); **HRMS** (ESI) m/z: [M + Na]$^+$ Calcd for $C_{14}H_{10}F_2O_3SNa$ 319.0216; Found 319.0224.

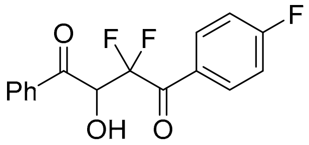

3r

2,2-difluoro-1-(4-fluorophenyl)-3-hydroxy-4-phenylbutane-1,4-dione (3r): pale yellow solid, 302.1 mg, 98% yield. M.p.: 56–57 ºC; **^{1}H NMR** (400 MHz, $CDCl_3$) δ 8.11 (dd, *J* = 8.3, 5.7 Hz, 2H), 8.03 (d, *J* = 8.0 Hz, 2H), 7.68 (t, *J* = 7.4 Hz, 1H), 7.54 (t, *J* = 7.8 Hz, 2H), 7.16 (t, *J* = 8.6 Hz, 2H), 5.70 (dd, *J* = 17.4, 4.6 Hz, 1H), 4.25 (d, *J* = 7.1 Hz, 1H); **^{19}F NMR** (376 MHz, $CDCl_3$) δ –100.35 – –101.10 (m, 1F), –101.71 – –101.78 (m, 1F), –112.69 (dd, *J* = 277.7, 17.5 Hz, 1F); **^{13}C{^{1}H} NMR** (100 MHz, $CDCl_3$) δ 195.0, 187.9 (C−F, dd, $^{2}J_{C-F}$ = 29.9, 27.2 Hz), 166.5 (C−F, d, $^{1}J_{C-F}$ = 258.0 Hz), 134.8, 134.4, 133.2 (C−F, ddd, $^{3}J_{C-F}$, *J* = 9.5, 4.6, 2.8 Hz), 129.5 (C−F, d, $^{3}J_{C-F}$ = 1.3 Hz), 128.8, 116.0 (C−F, d, $^{2}J_{C-F}$ = 22.0 Hz), 116.0 (C−F, dd, $^{1}J_{C-F}$ = 265.9, 260.0 Hz), 72.3 (C−F, t, $^{2}J_{C-F}$ = 27.0 Hz); **HRMS** (ESI) m/z: [M + Na]$^+$ Calcd for $C_{16}H_{11}F_3O_3Na$ 331.0558; Found 331.0565.

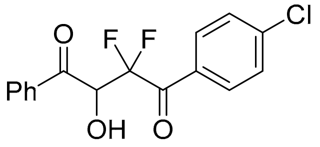

3s

1-(4-chlorophenyl)-2,2-difluoro-3-hydroxy-4-phenylbutane-1,4-dione (3s): pale yellow solid, 314.8 mg, 97% yield. M.p.: 62–64 ºC; **^{1}H NMR** (400 MHz, $CDCl_3$) δ 8.01 (dd, *J* = 12.7, 8.3 Hz, 4H), 7.67 (t, *J* = 7.4 Hz, 1H), 7.53 (t, *J* = 7.8 Hz, 2H), 7.45 (d, *J* = 8.7 Hz, 2H), 5.70 (ddd, *J* = 17.7, 6.7, 4.1 Hz, 1H) 4.29 (d, *J* = 7.4 Hz, 1H); **^{19}F NMR** (376 MHz, $CDCl_3$) δ –100.81 (d, *J* = 276.4 Hz, 1F), –113.07 (dd, *J* = 277.9, 18.1 Hz, 1F); **^{13}C{^{1}H} NMR** (100 MHz, $CDCl_3$) δ 194.9, 188.3 (C−F, dd, $^{2}J_{C-F}$ = 30.1, 27.1 Hz), 141.2, 134.8, 134.3, 131.5 (C−F, dd, $^{3}J_{C-F}$ = 4.5, 2.5 Hz), 130.9 (C−F, d, $^{3}J_{C-F}$ = 1.5 Hz), 129.4 (C−F, d, $^{3}J_{C-F}$ = 1.3 Hz), 129.0, 128.8, 115.8 (C−F, dd, $^{1}J_{C-F}$ = 265.8, 261.8 Hz), 72.2 (C−F, t, $^{2}J_{C-F}$ = 26.9 Hz); **HRMS** (ESI) m/z: [M + Na]$^+$ Calcd for

$C_{16}H_{11}ClF_2O_3Na$ 347.0262; Found 347.0269.

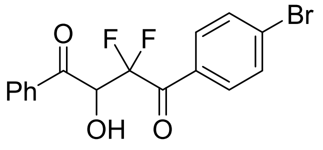

3t

1-(4-bromophenyl)-2,2-difluoro-3-hydroxy-4-phenylbutane-1,4-dione (3t): pale yellow solid, 258.3 mg, 70% yield. M.p.: 93–94 ºC; **^{1}H NMR** (400 MHz, $CDCl_3$) δ 8.02 (dd, J = 11.9, 8.0 Hz, 4H), 7.72 – 7.60 (m, 3H), 7.53 (t, J = 7.7 Hz, 2H), 5.72 (ddd, J = 17.9, 7.3, 3.8 Hz, 1H), 4.24 (d, J = 7.6 Hz, 1H); **^{19}F NMR** (376 MHz, $CDCl_3$) δ –100.65 (d, J = 277.1 Hz, 1F), –113.31 (dd, J = 277.6, 17.5 Hz, 1F); **^{13}C{^{1}H} NMR** (100 MHz, $CDCl_3$) δ 195.1, 189.5 (C−F, dd, $^2J_{C-F}$ = 29.6, 26.9 Hz), 149.3, 134.7, 134.4, 133.7, 133.5, 132.6 (C−F, d, $^3J_{C-F}$ = 1.9 Hz), 129.5 (C−F, d, $^3J_{C-F}$ = 1.3 Hz), 128.8 (C−F, dd, $^3J_{C-F}$ = 3.9, 2.8 Hz), 128.8, 126.7 (C−F, d, $^3J_{C-F}$ = 2.3 Hz), 115.9 (C−F, dd, $^1J_{C-F}$ = 266.0, 261.5 Hz), 72.3 (C−F, t, $^2J_{C-F}$ = 27.0 Hz); **HRMS** (ESI) m/z: $[M + Na]^+$ Calcd for $C_{16}H_{11}BrF_2O_3Na$ 390.1054; Found 390.1060.

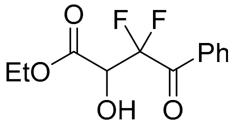

3u

ethyl 3,3-difluoro-2-hydroxy-4-oxo-4-phenylbutanoate (3u): The crude products were purified by column chromatography with petroleum ether/ethyl acetate (15/1, v/v) as eluent to give compound **3y** (pale yellow oil, 121.3 mg, 47% yield). **^{1}H NMR** (400 MHz, $CDCl_3$) δ 8.08 (d, J = 7.7 Hz, 2H), 7.65 (t, J = 7.4 Hz, 1H), 7.51 (t, J = 7.8 Hz, 2H), 4.76 (t, J = 9.7 Hz, 1H), 4.55 – 4.17 (m, 2H), 3.42 (d, J = 4.2 Hz, 1H), 1.29 (t, J = 7.1 Hz, 3H); **^{19}F NMR** (376 MHz, $CDCl_3$) δ –106.89 (dd, J = 287.9, 8.8 Hz, 1F), –109.08 (dd, J = 287.5, 12.5 Hz, 1F); **^{13}C{^{1}H} NMR** (100 MHz, $CDCl_3$) δ 188.4 (C−F, t, $^2J_{C-F}$ = 28.6 Hz), 168.8 (C−F, t, $^3J_{C-F}$ = 3.0 Hz), 134.6, 132.2 (C−F, t, $^3J_{C-F}$ = 2.2 Hz), 130.0 (C−F, t, $^3J_{C-F}$ = 3.3 Hz), 128.8, 115.5 (C−F, t, $^1J_{C-F}$ = 262.8 Hz), 71.0 (C−F, t, $^2J_{C-F}$ = 26.8 Hz), 63.2, 13.9; **HRMS** (ESI) m/z: $[M + Na]^+$ Calcd for $C_{12}H_{12}F_2O_4Na$ 281.0601; Found 281.0608.

Optimization of the reaction conditions for the synthesis of polyfluoroarylated compounds

Table S1. Optimization of the reaction conditions [a].

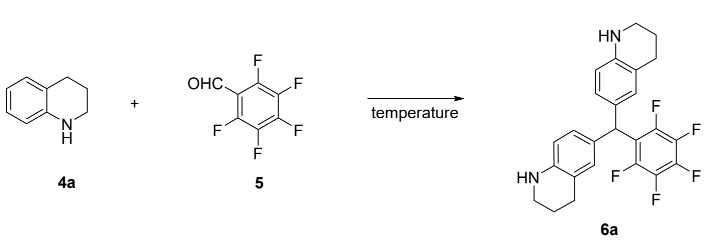

entry	temp (ºC)	time (h)	yield (%)[b]
1	25	6	53
2	40	6	54
3	60	6	61
4	80	6	65
5	100	6	43

[a] Reaction conditions: aniline **4a** (1.0 mmol), pentafluorobenzaldehyde **5** (0.5 mmol, 2.0 equiv) at

indicated temperature unless otherwise noted. [b] Isolated yield.

General procedure for the synthesis of polyfluoroarylated compounds

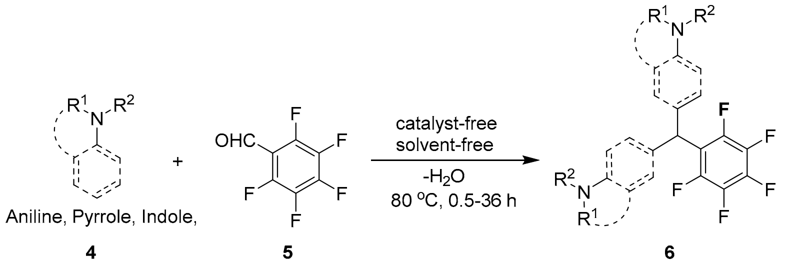

To a 5.0 mL oven-dried Schlenk flask was added aniline, pyrrole or indole **4** (1.0 mmol) and pentafluoro-benzaldehyde **5** (0.5 mmol) under argon atmosphere. The resulting mixture was stirred at 80 °C until the completion of the reaction (monitored by TLC, approximately 0.5–36 hours). Then, the reaction mixture was separated by column chromatography (petroleum ether/ethyl acetate) to give the pure polyfluoroarylated products.

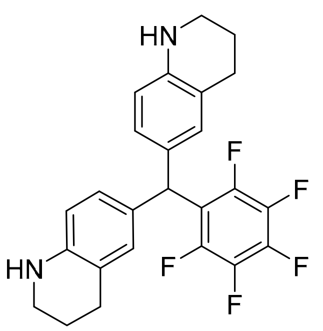

6a

6,6'-((perfluorophenyl)methylene)bis(1,2,3,4-tetrahydroquinoline) (6a): The crude products were purified by column chromatography with petroleum ether/ethyl acetate (10/1, v/v) as eluent to give compound **6a (**white solid, 144.6 mg, 65% yield). M.p.: 93–95 °C; **^{1}H NMR** (400 MHz, $CDCl_3$) δ 6.76 (d, J = 9.5 Hz, 4H), 6.56 – 6.28 (m, 2H), 5.57 (s, 1H), 3.80 (s, 2H), 3.38 – 3.14 (m, 4H), 2.69 (t, J = 6.4 Hz, 4H), 2.02 – 1.77 (m, 4H); **^{19}F NMR** (376 MHz, $CDCl_3$) δ –139.98 (dd, J = 24.3, 7.2 Hz, 2F), –157.72 (t, J = 21.6 Hz, 1F), –162.43 (td, J = 23.4, 8.8 Hz, 2F); **^{13}C{^{1}H} NMR** (100 MHz, $CDCl_3$) δ 146.4 – 146.2 (m), 143.9 – 143.7 (m), 143.5, 136.6 – 136.3 (m), 136.7 – 136.2 (m), 129.6, 128.8, 126.9, 121.3, 119.1 – 118.7 (m), 114.0, 44.4, 41.9, 27.0, 22.1; **HRMS** (ESI) m/z: $[M - H]^-$ Calcd for $C_{25}H_{20}F_5N_2$ 443.1541; Found 443.1537.

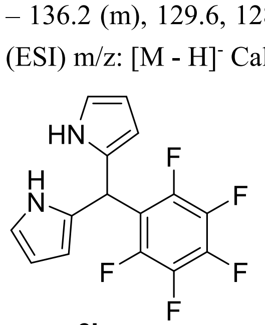

6b

2,2'-((perfluorophenyl)methylene)bis(1*H*-pyrrole) (6b): The crude products were purified by column chromatography with petroleum ether/ethyl acetate (20/1, v/v) as eluent to give compound **6b (**light yellow solid, 110.8 mg, 71% yield). M.p.: 131–132 °C; **^{1}H NMR** (400 MHz, $CDCl_3$) δ 8.2 (s, 2H), 6.74 (d, 2H), 6.15 (t, 2H), 6.00 (d, 2H), 5.92 (s, 1H); **^{19}F NMR** (376 MHz, $CDCl_3$) δ

-140.50 – -142.56 (m, 2F), -154.70 – -156.76 (m, 2F), -160.24 – -162.08 (m, 2F); **^{13}C{^{1}H} NMR** (100 MHz, $CDCl_3$) δ 146.2 – 146.0 (m), 143.7 – 143.6 (m), 139.2 – 139.0 (m), 136.7 – 136.2 (m), 128.1, 118.1, 115.6 – 115.3 (m), 108.7, 107.7, 33.1; **HRMS** (ESI) m/z: $[M - H]^-$ Calcd for $C_{15}H_8F_5N_2$ 311.0602; Found 311.0594.

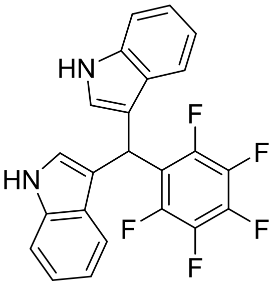

6c

3,3'-((perfluorophenyl)methylene)bis(1*H*-indole) (6c): The crude products were purified by column chromatography with petroleum ether/ethyl acetate (10/1, v/v) as eluent to give compound **6d** (white solid, 121.5 mg, 59% yield). M.p.: 126–128 °C; **^{1}H NMR** (400 MHz, $CDCl_3$) δ 8.06 (s, 2H), 7.41 (dd, *J* = 10.5, 8.3 Hz, 4H), 7.21 (t, *J* = 7.6 Hz, 2H), 7.08 (t, *J* = 7.5 Hz, 2H), 6.98 (s, 2H), 6.33 (s, 1H); **^{19}F NMR** (376 MHz, $CDCl_3$) δ –141.32 (d, *J* = 28.9 Hz, 2F), –157.14 (t, *J* = 21.4 Hz, 1F), –162.04 (td, *J* = 23.2, 8.7 Hz, 2F); **^{13}C{^{1}H} NMR** (100 MHz, $CDCl_3$) δ 136.3, 126.5, 123.5 – 123.2 (m), 122.3, 119.7, 118.9, 115.0, 111.3, 29.1; **HRMS** (ESI) m/z: $[M - H]^-$ Calcd for $C_{23}H_{12}F_5N_2$ 411.0915; Found 411.0910.

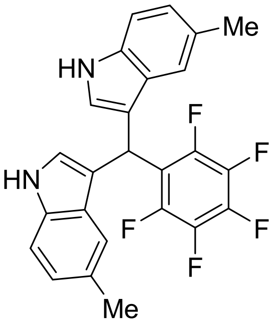

6d

3,3'-((perfluorophenyl)methylene)bis(5-methyl-1*H*-indole) (6d): The crude products were purified by column chromatography with petroleum ether/ethyl acetate (10/1, v/v) as eluent to give compound **6d** (white solid, 138.3 mg, 63% yield). M.p.: 130–131 °C; **^{1}H NMR** (400 MHz, Acetone-*d6*) δ 10.09 (s, 2H), 7.31 (d, *J* = 8.3 Hz, 2H), 7.18 (s, 2H), 7.06 (s, 2H), 6.95 (d, *J* = 8.3 Hz, 2H), 6.30 (s, 1H), 2.33 (s, 6H); **^{19}F NMR** (376 MHz, Acetone-*d6*) δ -142.76 – -144.05 (m, 2F), -160.28 (ddd, *J* = 68.8, 35.7, 22.1 Hz, 2F), -164.31 – -165.40 (m, 2F); **^{13}C{^{1}H} NMR** (100 MHz, Acetone-*d6*) δ 147.3, 144.9, 140.0 – 139.4 (m), 136.0, 128.6, 127.8, 124.7, 124.0, 118.8, 114.6, 112.2, 21.6, 21.5; **HRMS** (ESI) m/z: $[M - H]^-$ Calcd for $C_{25}H_{16}F_5N_2$ 439.1239; Found 439.1232.

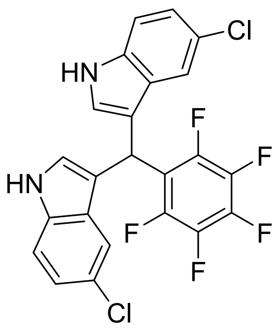

6e

3,3'-((perfluorophenyl)methylene)bis(5-chloro-1*H*-indole) (6e): The crude products were purified by column chromatography with petroleum ether/ethyl acetate (10/1, v/v) as eluent to give compound **6d** (white solid, 110.6 mg, 46% yield). M.p.: 133–134 ºC; **^{1}H NMR** (400 MHz, Acetone-*d6*) δ 10.45 (s, 2H), 7.46 (d, *J* = 8.6 Hz, 2H), 7.37 (d, *J* = 2.0 Hz, 2H), 7.21 (s, 2H), 7.11 (dd, *J* = 8.6, 2.0 Hz, 2H), 6.36 (s, 1H); **^{19}F NMR** (376 MHz, Acetone-*d6*) δ -143.01 (dd, *J* = 21.7, 7.1 Hz, 2F), -159.47 (t, *J* = 20.6 Hz, 1F), -164.22 (td, *J* = 21.9, 7.6 Hz, 2F); **^{13}C{^{1}H} NMR** (100 MHz, Acetone-*d6*) δ 147.5 – 147.1, 142.3 – 141.9, 137.6 – 137.2, 136.3, 128.6, 126.7, 125.2, 122.6, 118.8, 114.7, 113.9; **HRMS** (ESI) m/z: [M - H]$^-$ Calcd for $C_{23}H_{10}F_5Cl_2N_2$ 479.0147; Found 479.0154.

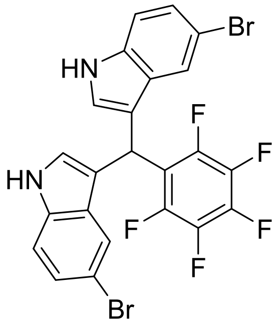

6f

3,3'-((perfluorophenyl)methylene)bis(5-bromo-1*H*-indole) (6f): The crude products were purified by column chromatography with petroleum ether/ethyl acetate (10/1, v/v) as eluent to give compound **6d** (white solid, 145.3 mg, 51% yield). M.p.: 136–138 ºC; **^{1}H NMR** (400 MHz, Acetone-*d6*) δ 10.46 (s, 1H), 7.54 (d, *J* = 1.7 Hz, 2H), 7.42 (d, *J* = 8.6 Hz, 2H), 7.24 (dd, *J* = 8.6, 1.9 Hz, 2H), 7.19 (s, 2H), 6.37 (s, 1H); **^{19}F NMR** (376 MHz, Acetone-*d6*) δ -143.01 (dd, *J* = 21.4, 6.3 Hz, 2F), -159.44 (t, *J* = 20.6 Hz, 1F), -164.21 (td, *J* = 21.7, 7.5 Hz, 2F); **^{13}C{^{1}H} NMR** (100 MHz, Acetone-*d6*) δ 145.1 – 144.6 (m), 139.8 (ddt, *J* = 13.4, 7.9, 3.7 Hz), 136.5, 129.3, 126.5, 125.2, 121.9, 114.6, 114.4, 112.8; **HRMS** (ESI) m/z: [M - H]$^-$ Calcd for $C_{23}H_{10}F_5Br_2N_2$ 566.9136; Found 566.9138.

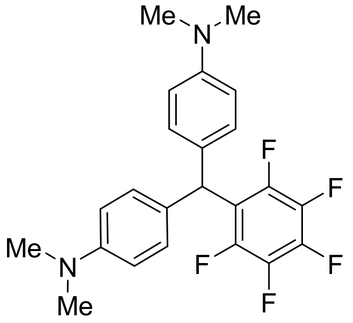

6g

4,4'-((perfluorophenyl)methylene)bis(*N*,*N*-dimethylaniline) (6g): The crude products were purified by column chromatography with petroleum ether/ethyl acetate (20/1, v/v) as eluent to give compound **6g** (pale green solid, 100.9 mg, 48% yield). M.p.: 120–122 ºC; **^{1}H NMR** (400 MHz, $CDCl_3$) δ 7.06 (d, *J* = 8.6 Hz, 4H), 6.67 (d, *J* = 8.8 Hz, 4H), 5.71 (s, 1H), 2.93 (s, 12H); **^{19}F NMR** (376 MHz, $CDCl_3$) δ –140.09 (dd, *J* = 23.8, 8.7 Hz, 2F), –157.48 (t, *J* = 21.4 Hz, 1F), –162.33 (td, *J* = 23.5, 8.9 Hz, 2F); **^{13}C{^{1}H} NMR** (100 MHz, $CDCl_3$) δ 149.4, 146.5 – 146.1 (m), 144.0 – 143.5 (m), 141.1 – 140.8 (m), 139.0 – 138.3 (m), 136.7 – 136.1 (m), 129.2, 128.3, 119.0 – 118.4 (m), 112.4, 44.1, 40.5; **HRMS** (ESI) m/z: $[M + Na]^+$ Calcd for $C_{23}H_{21}F_5N_2Na$ 443.1517; Found 443.1523.

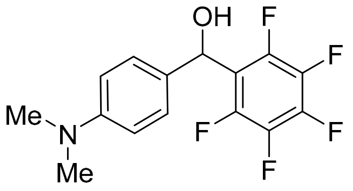

6g'

(4-(dimethylamino)phenyl)(perfluorophenyl)methanol (6g′): The crude products were purified by column chromatography with petroleum ether/ethyl acetate (10/1, v/v) as eluent to give compound **6g′** (white solid, 20.6 mg, 13% yield). M.p.: 87–88 ºC; **^{1}H NMR** (400 MHz, $CDCl_3$) δ 7.24 (d, *J* = 8.8 Hz, 2H), 6.73 – 6.66 (m, 2H), 6.14 (d, *J* = 7.1 Hz, 1H), 2.95 (s, 6H), 2.55 (d, *J* = 7.6 Hz, 1H); **^{19}F NMR** (376 MHz, $CDCl_3$) δ –143.21 (dd, *J* = 23.5, 9.1 Hz, 2F), –155.58 (t, *J* = 21.5 Hz, 2F), –161.83 (td, J = 23.5, 9.0 Hz, 2F); **^{13}C{^{1}H} NMR** (100 MHz, $CDCl_3$) δ 150.5, 145.9 – 145.6 (m), 143.6 – 142.9 (m), 139.6 – 138.3 (m), 136.6 – 135.9 (m), 128.0, 126.7, 117.5 – 117.2 (m), 112.3, 68.0, 40.4; **HRMS** (ESI) m/z: $[M + H]^+$ Calcd for $C_{15}H_{13}F_5NO$ 318.0912; Found 318.0904.

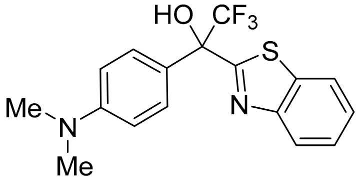

6h

1-(benzo[*d*]thiazol-2-yl)-1-(4-(dimethylamino)phenyl)-2,2,2-trifluoroethan-1-ol (6h): The crude products were purified by column chromatography with petroleum ether/ethyl acetate (15/1, v/v) as eluent to give compound **6h** (white solid, 72.3 mg, 41% yield). M.p.: 102–104 ºC; **^{1}H NMR** (400 MHz, $CDCl_3$) δ 8.08 (d, *J* = 8.2 Hz, 2H), 7.86 (d, *J* = 8.0 Hz, 1H), 7.64 (d, *J* = 8.8 Hz,

2H), 7.54−7.50 (m, 1H), 7.45−7.41 (m, 1H), 6.73 (d, J = 9.0 Hz, 2H), 5.12 (s, 1H), 2.97 (s, 6H); **^{19}F NMR** (376 MHz, $CDCl_3$) δ −75.96 (s, 3F); **$^{13}C\{^1H\}$ NMR** (100 MHz, $CDCl_3$) δ 169.4, 151.3, 150.8, 136.3, 127.4 (C−F, d, $^4J_{C-F}$ = 1.3 Hz), 126.5, 125.9, 124.2 (C−F, d, $^1J_{C-F}$ = 284.9 Hz), 123.7, 121.7, 111.8, 78.4 (C−F, d, $^2J_{C-F}$ = 29.9 Hz), 40.2; **HRMS** (ESI) m/z: $[M + H]^+$ Calcd for $C_{17}H_{16}F_3N_2OS$ 353.0930; Found 353.0938.

Reference

1. (a) P. Singh and G. Panda, *RSC Adv.*, 2014, **4**, 31892; (b) D. L. V. Jagt, L.-P. B. Han and C. H. Lehman, *J. Org. Chem.*, 1972, **37**, 4100; (c) A. Tafelska-Kaczmarek, A. Prewysz-Kwinto, K. Skowerski, K. Pietrasiak, A. Kozakiewicz and M. Zaidlewicz, *Tetrahedron: Asymmetry*, 2010, **21**, 2244.

2. H. Amii, T. Kobayashi, Y. Hatamoto and K. Uneyama, Chem. Commun., 1999, 1323.

NMR spectra of the related compounds

^{1}H NMR (400 MHz, $CDCl_3$) of **3a**

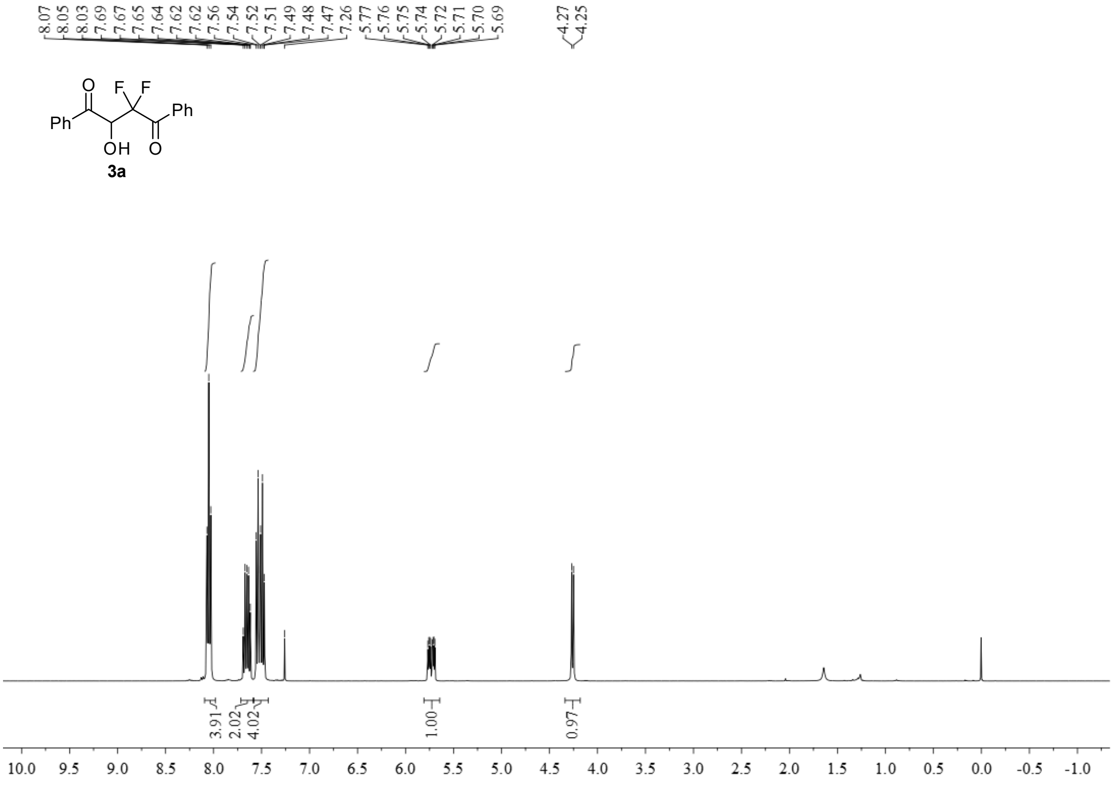

^{19}F NMR (376 MHz, $CDCl_3$) of **3a**

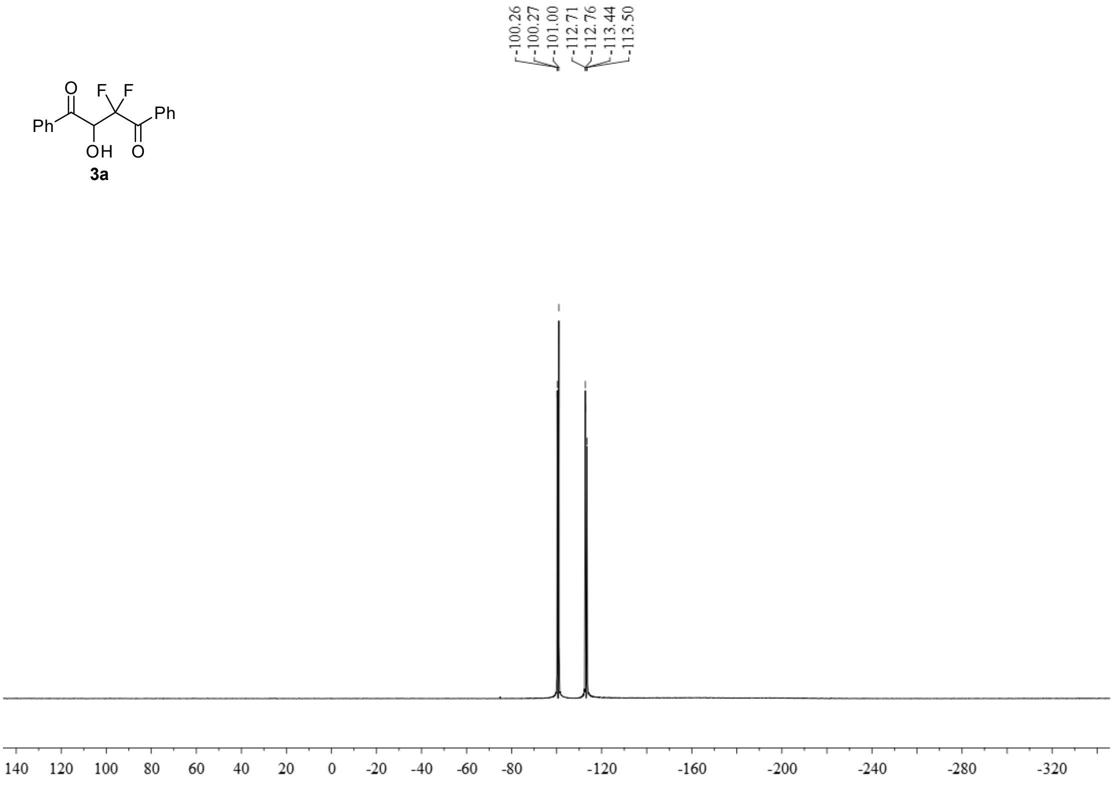

^{13}C NMR (100 MHz, $CDCl_3$) of **3a**

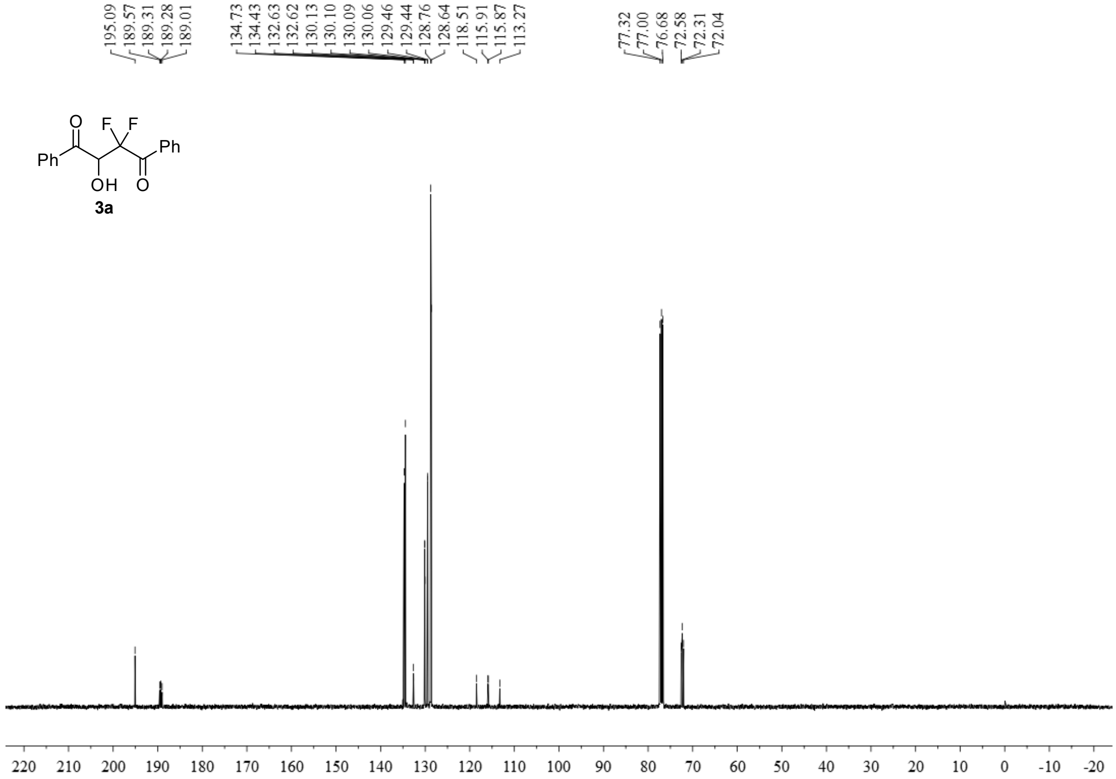

^{1}H NMR (400 MHz, $CDCl_3$) of **3b**

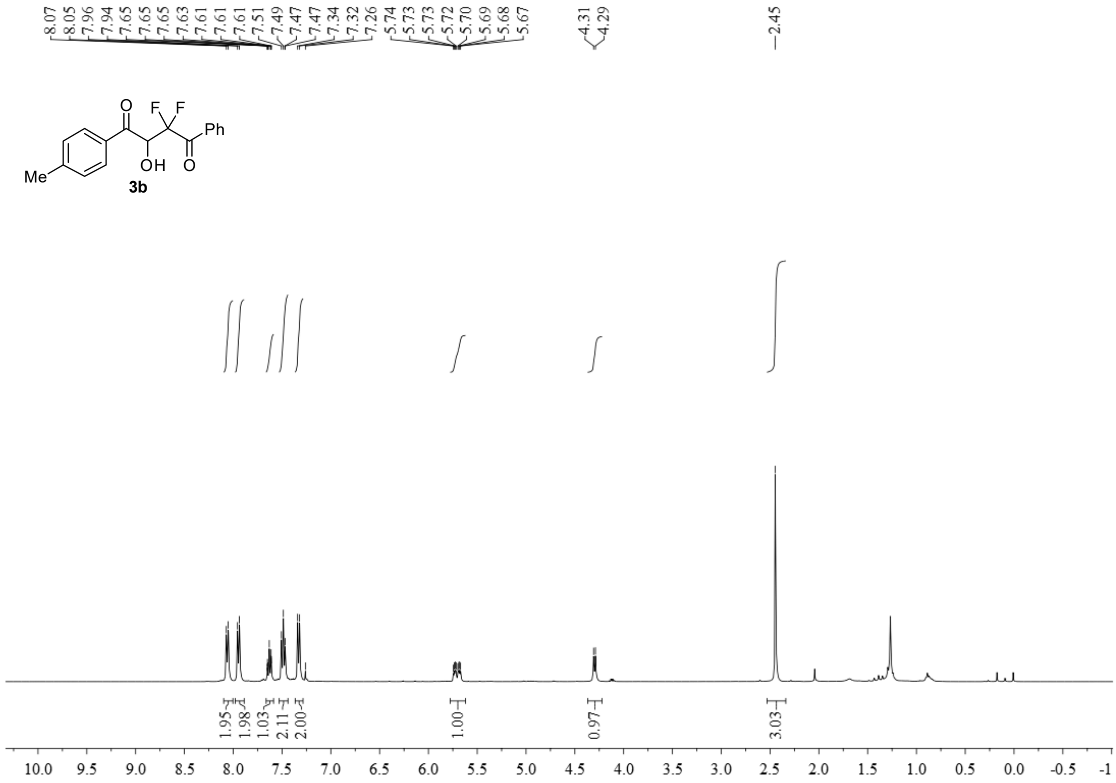

^{19}F NMR (376 MHz, $CDCl_3$) of **3b**

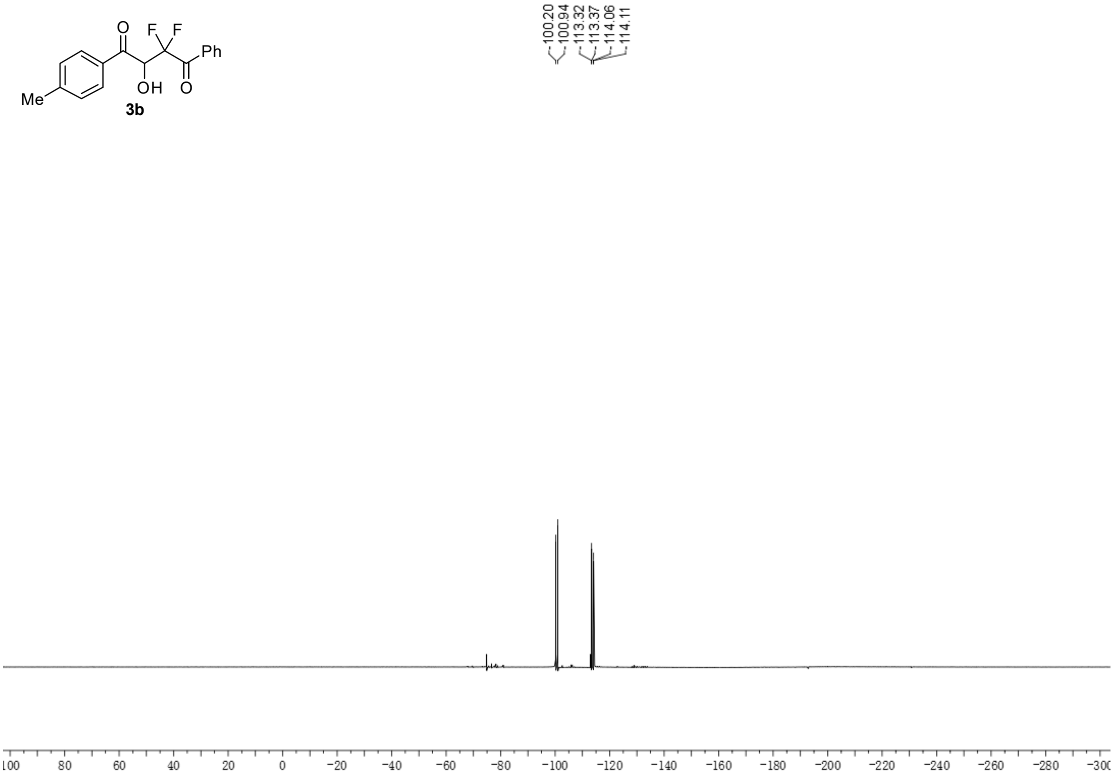

^{13}C NMR (100 MHz, $CDCl_3$) of **3b**

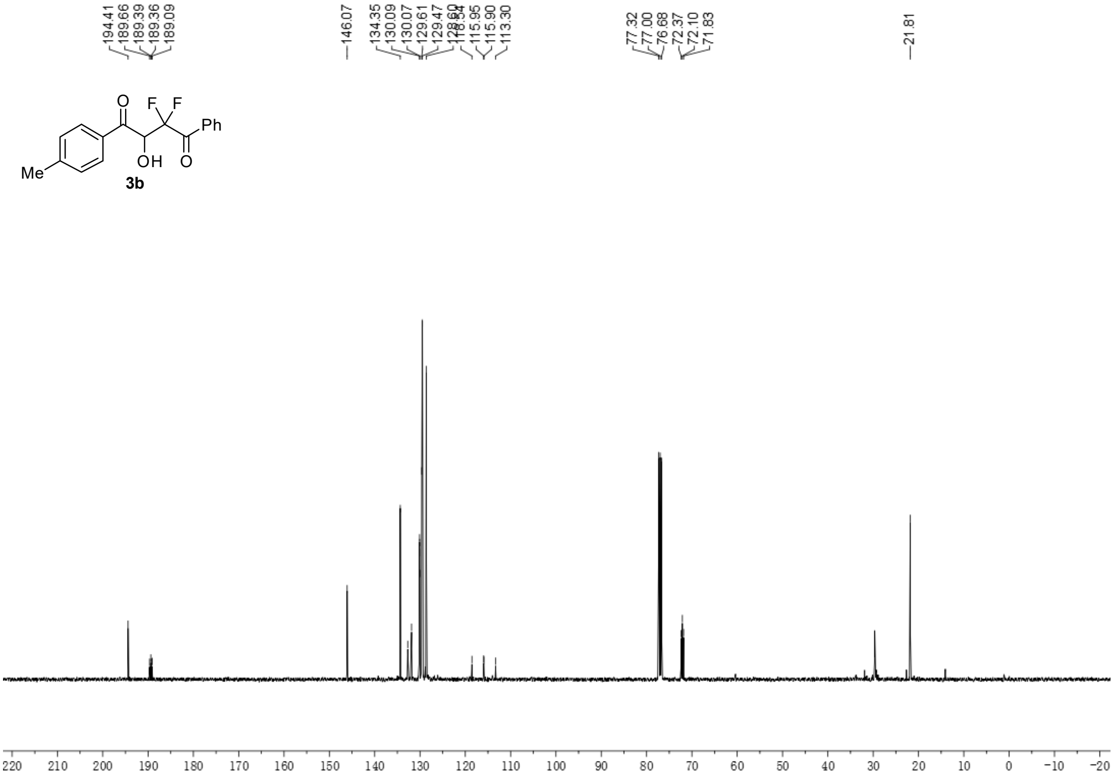

^{1}H NMR (400 MHz, $CDCl_3$) of **3c**

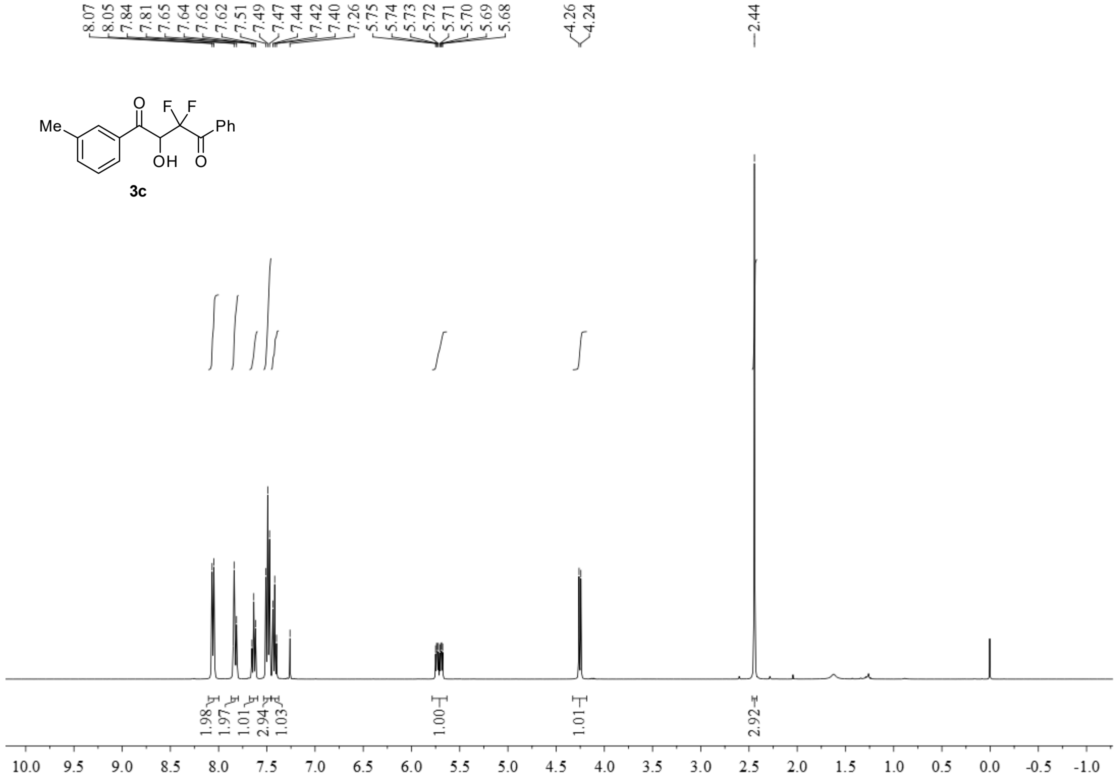

^{19}F NMR (376 MHz, $CDCl_3$) of **3c**

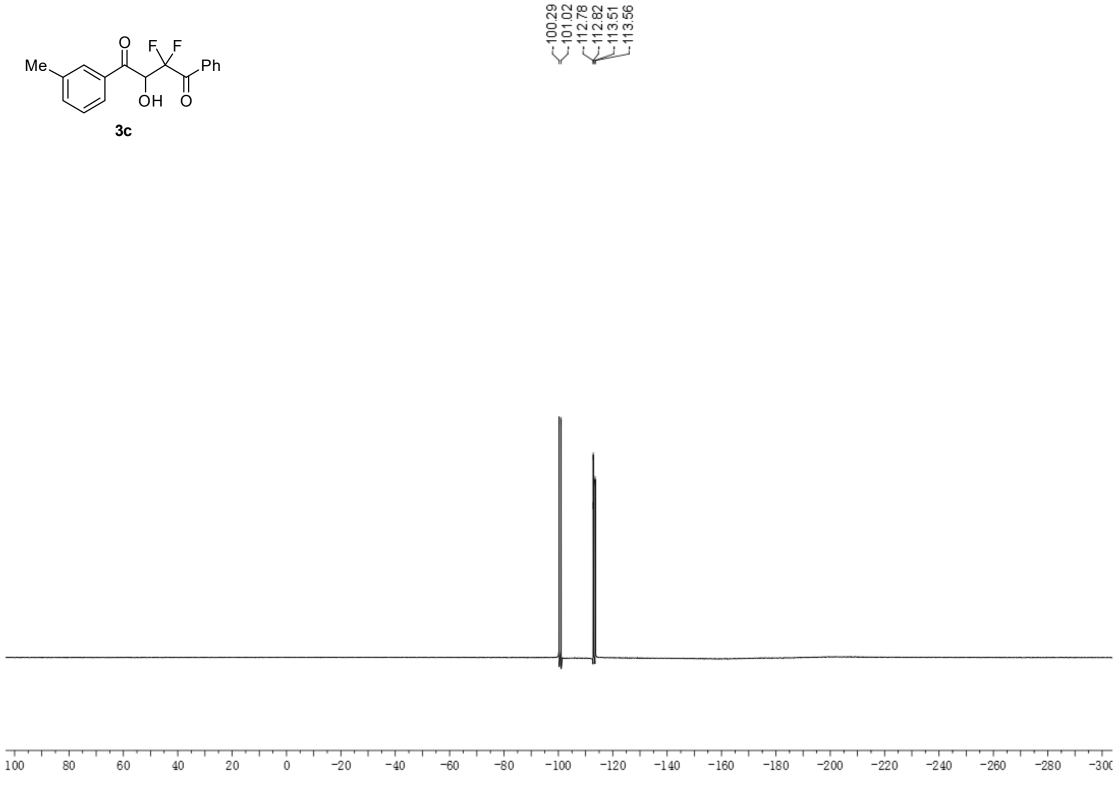

^{13}C NMR (100 MHz, $CDCl_3$) of **3c**

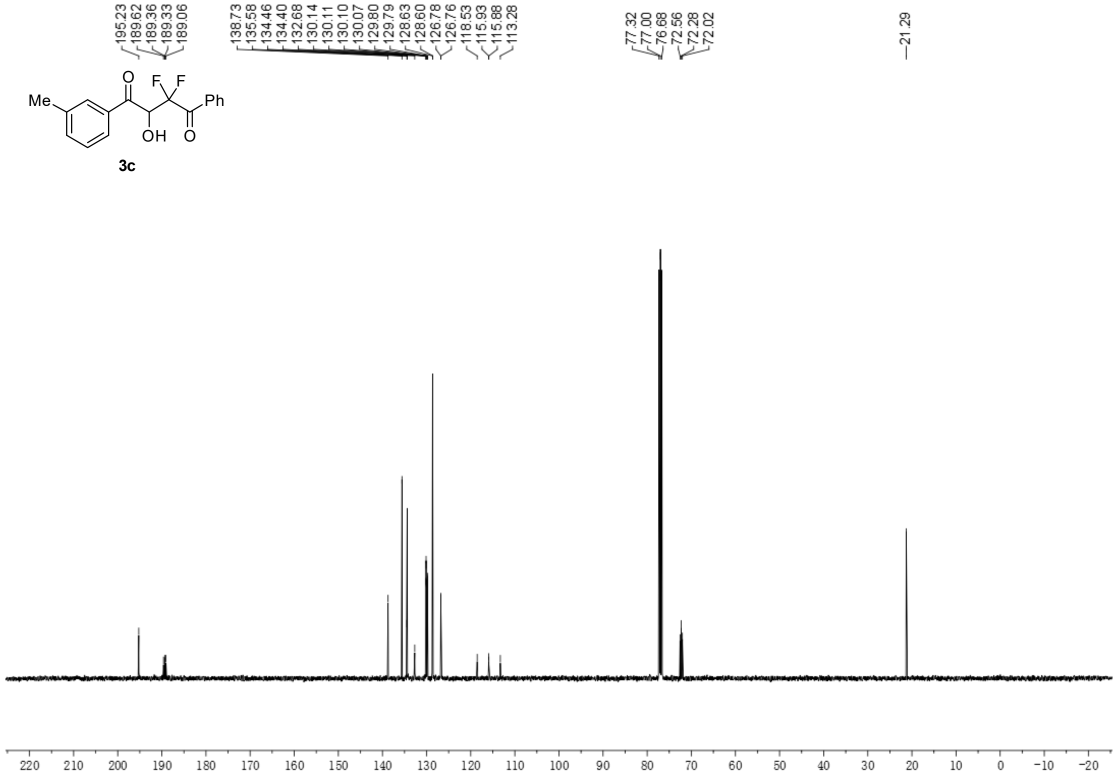

^{1}H NMR (400 MHz, $CDCl_3$) of **3d**

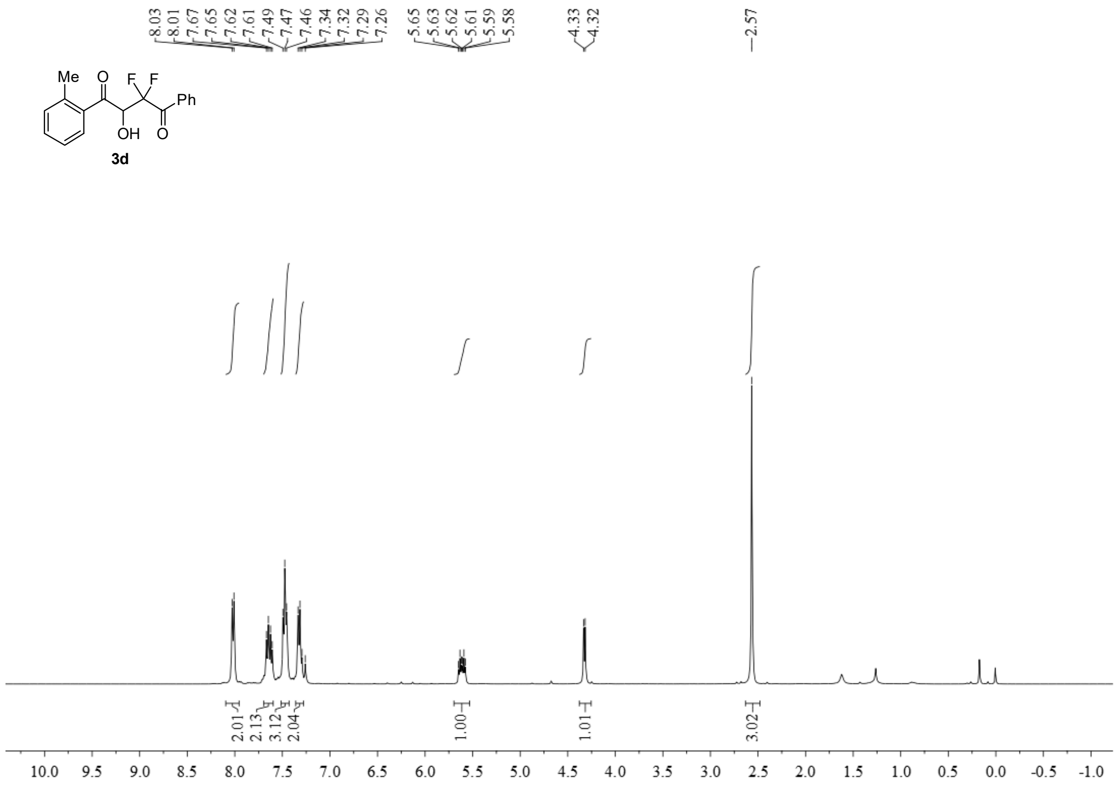

^{19}F NMR (376 MHz, $CDCl_3$) of **3d**

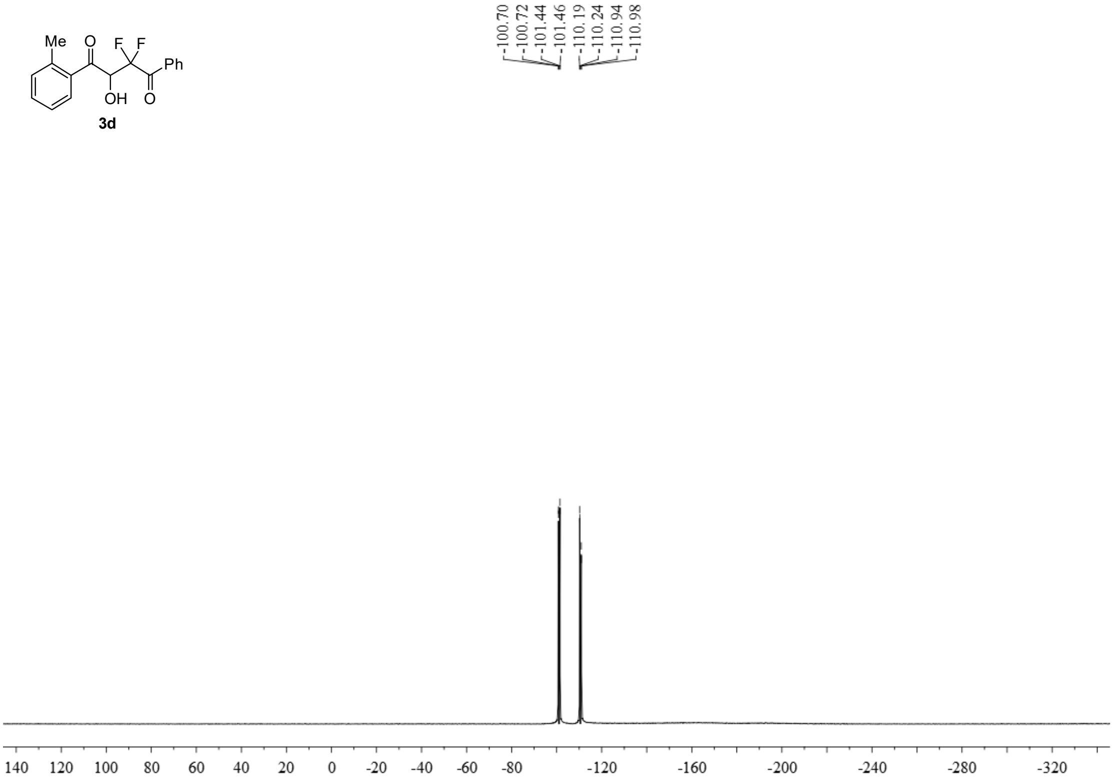

^{13}C NMR (100 MHz, $CDCl_3$) of **3d**

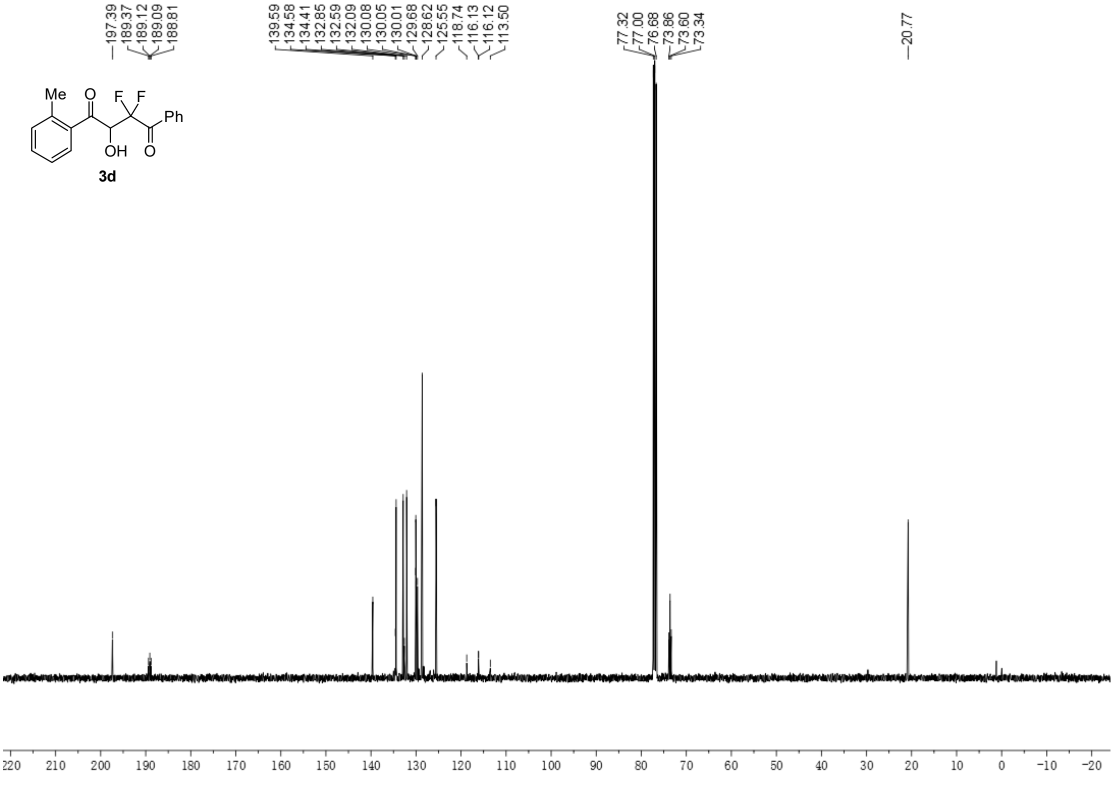

^{1}H NMR (400 MHz, $CDCl_3$) of **3e**

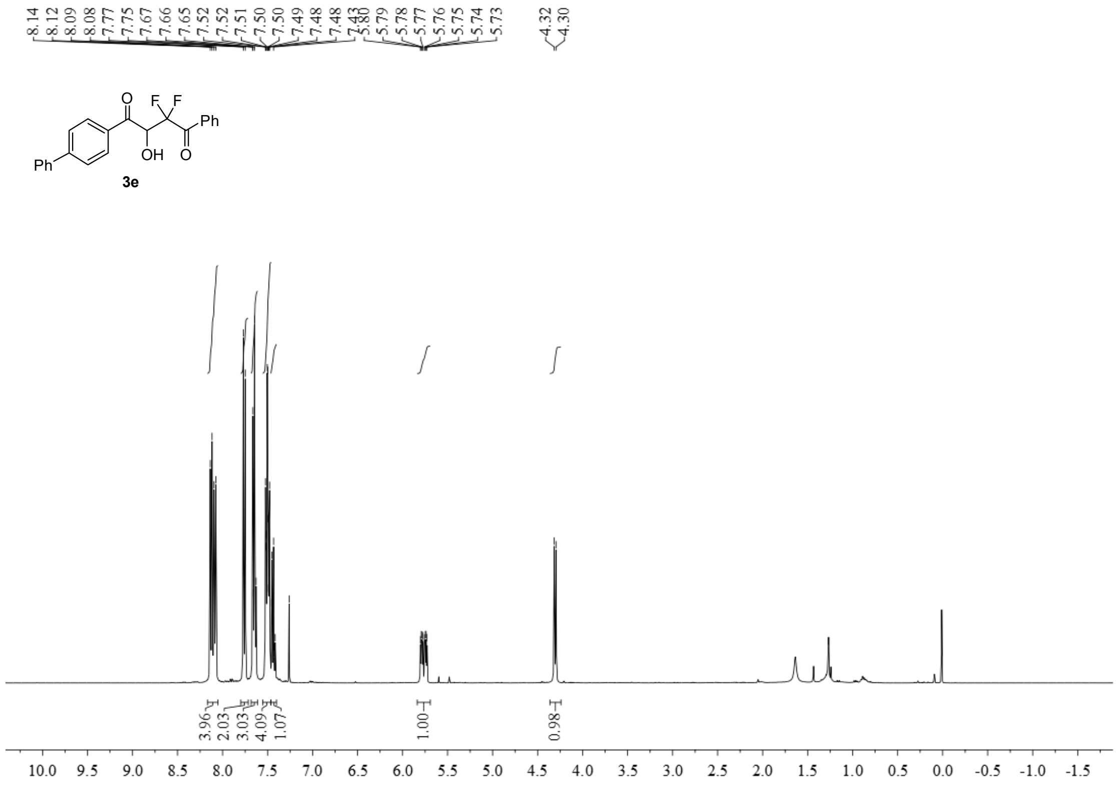

^{19}F NMR (376 MHz, $CDCl_3$) of **3e**

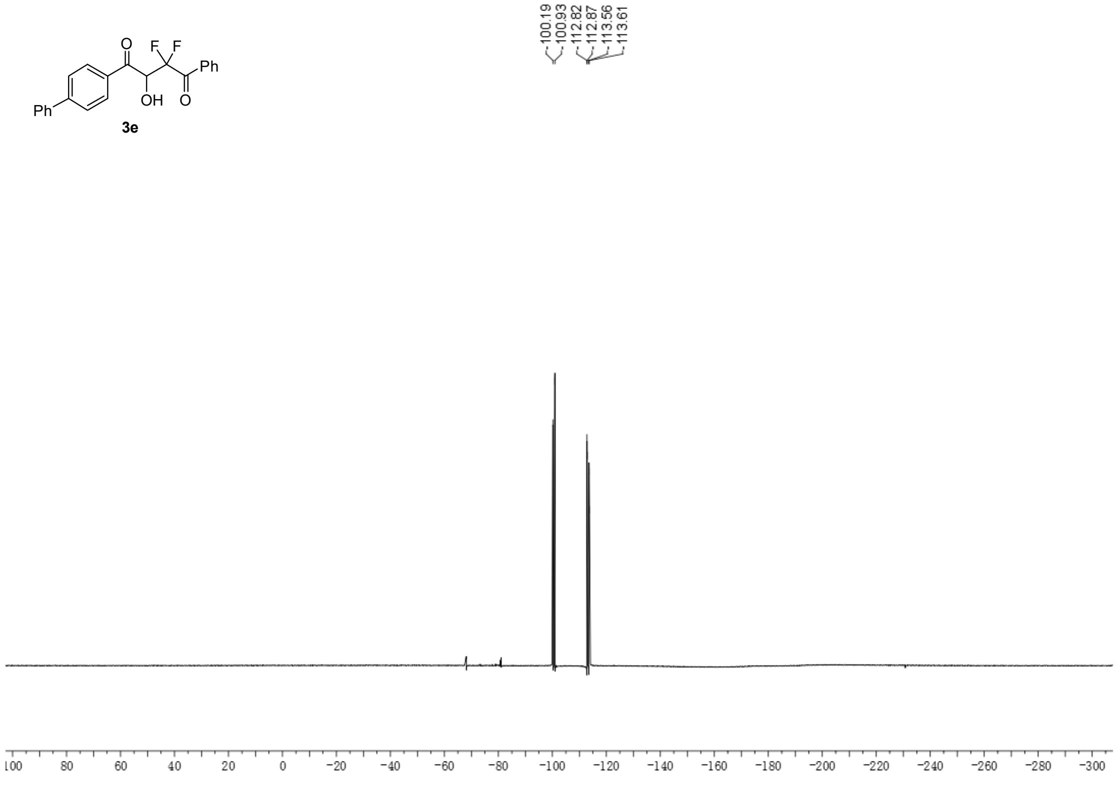

^{13}C NMR (100 MHz, $CDCl_3$) of **3e**

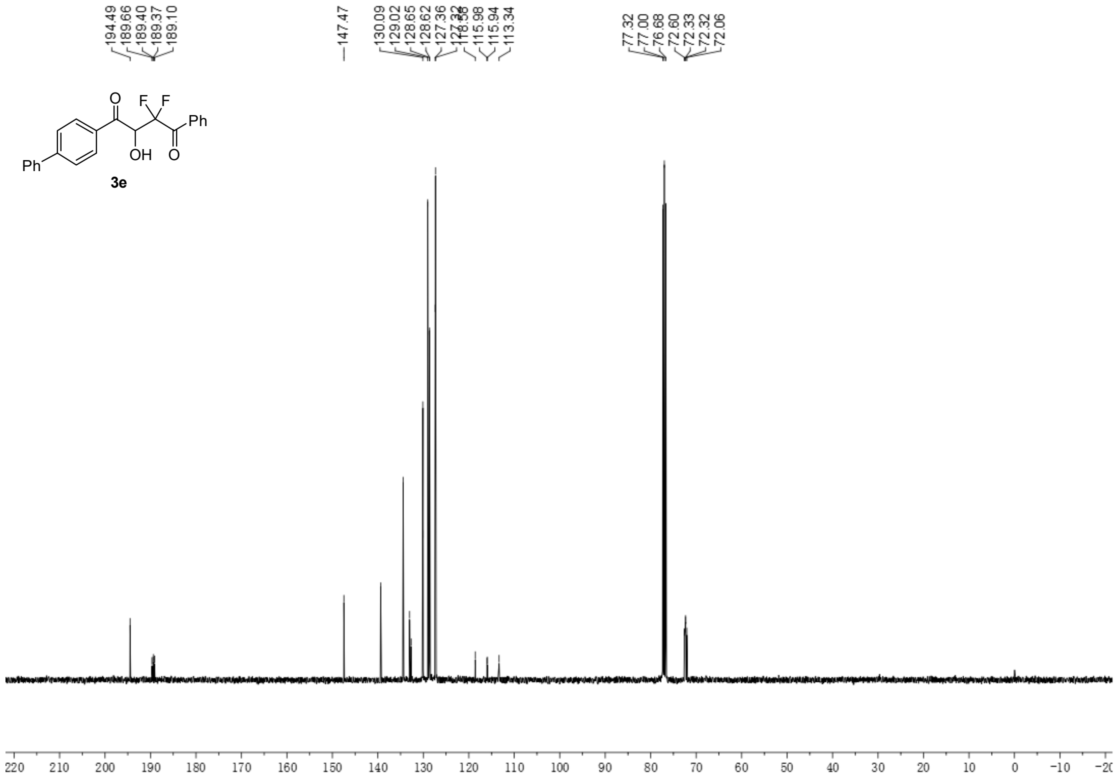

^{1}H NMR (400 MHz, $CDCl_3$) of **3f**

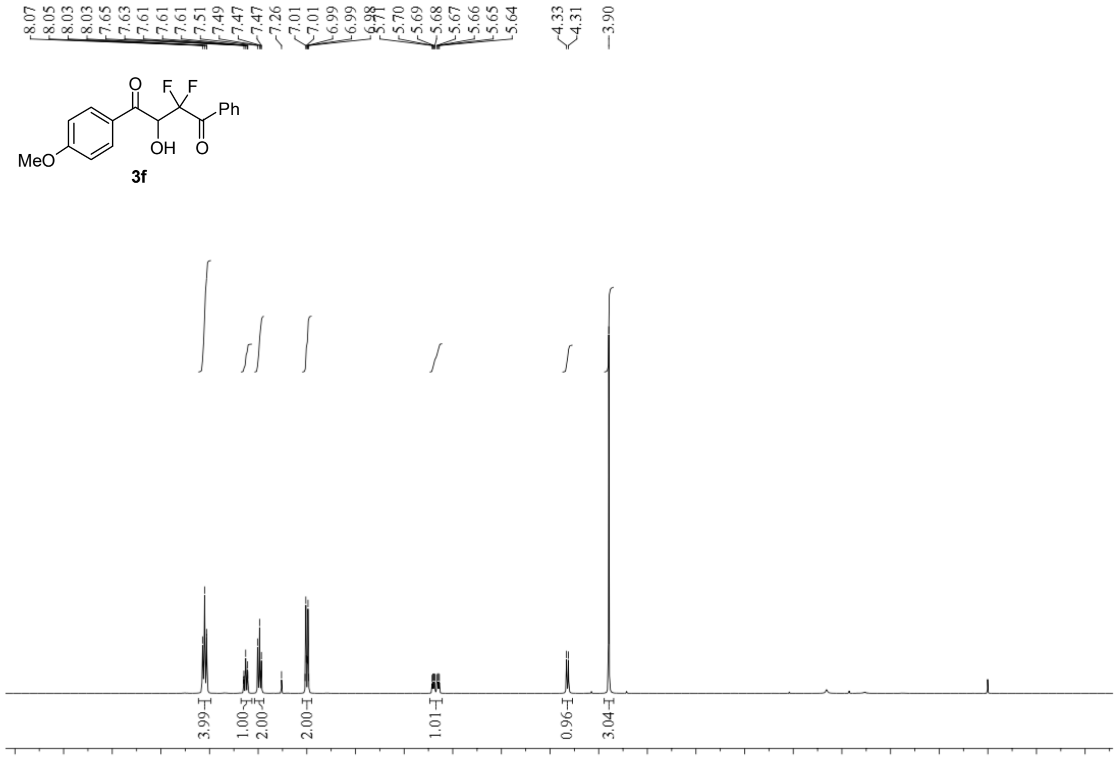

^{19}F NMR (376 MHz, $CDCl_3$) of **3f**

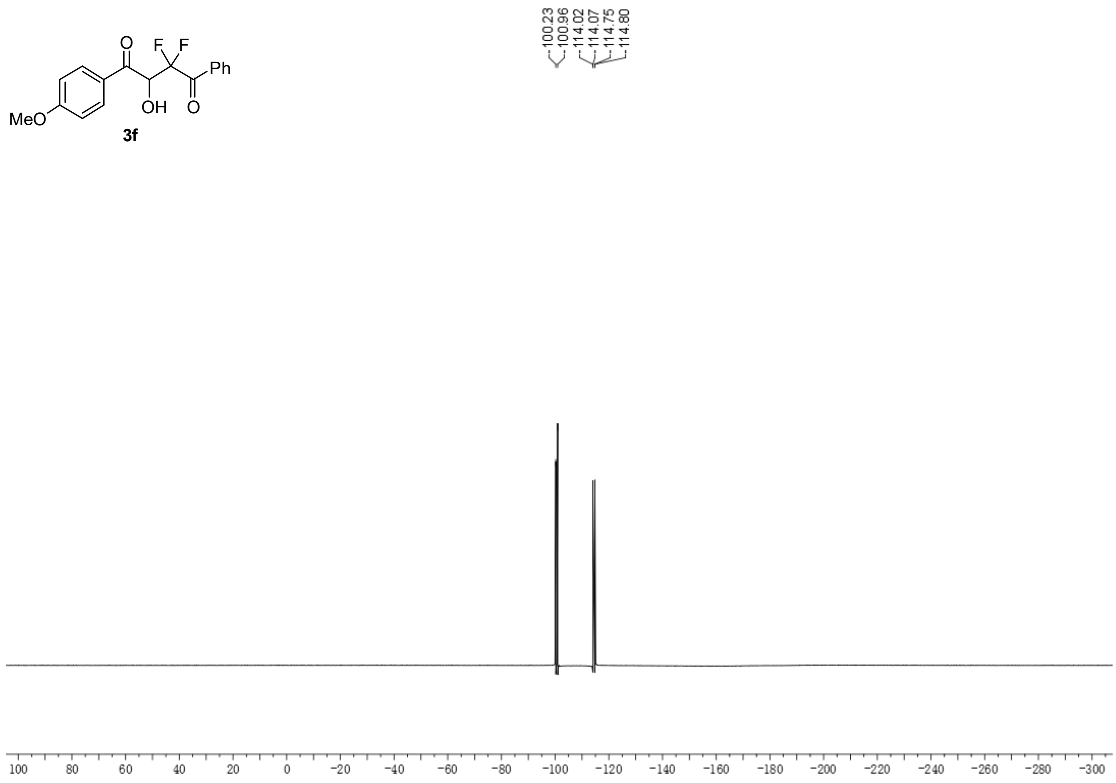

^{13}C NMR (100 MHz, $CDCl_3$) of **3f**

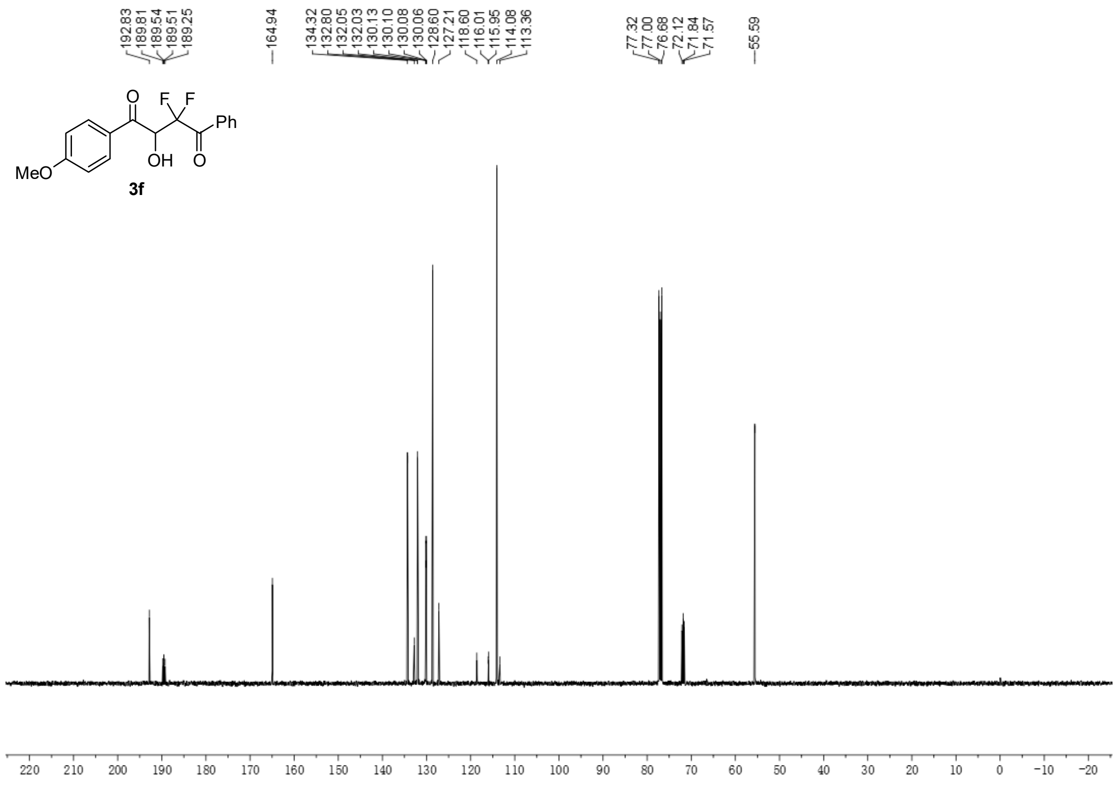

^{1}H NMR (400 MHz, $CDCl_3$) of **3g**

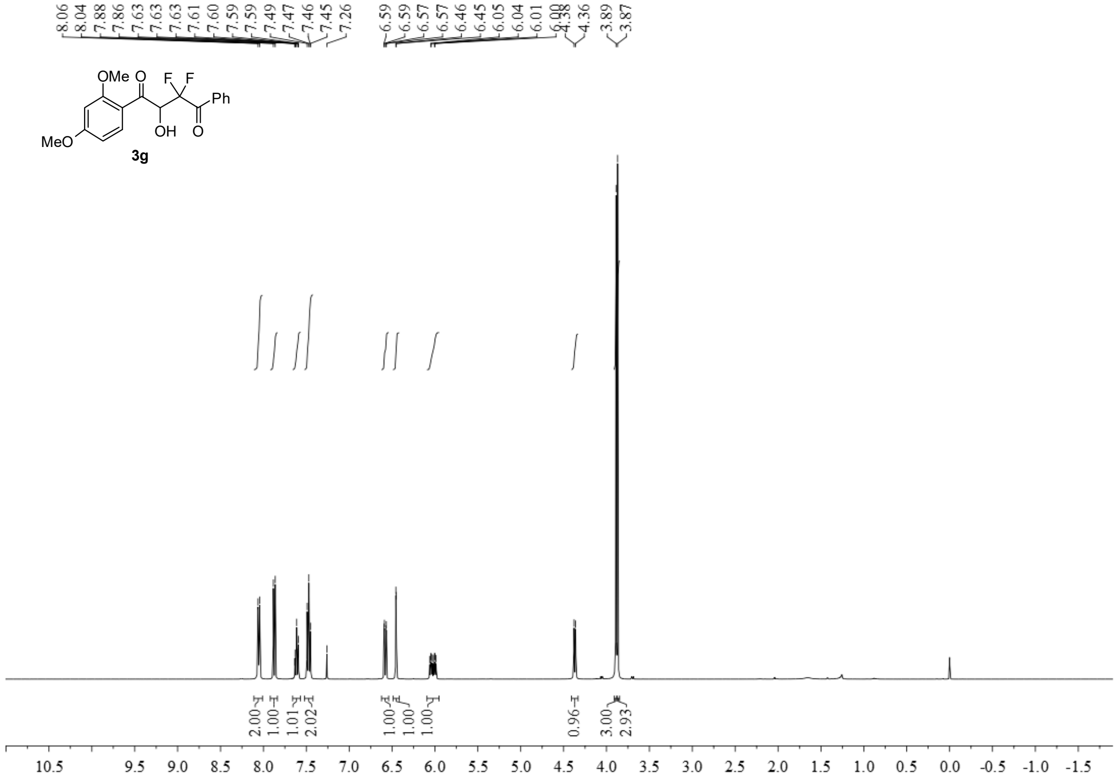

^{19}F NMR (376 MHz, $CDCl_3$) of **3g**

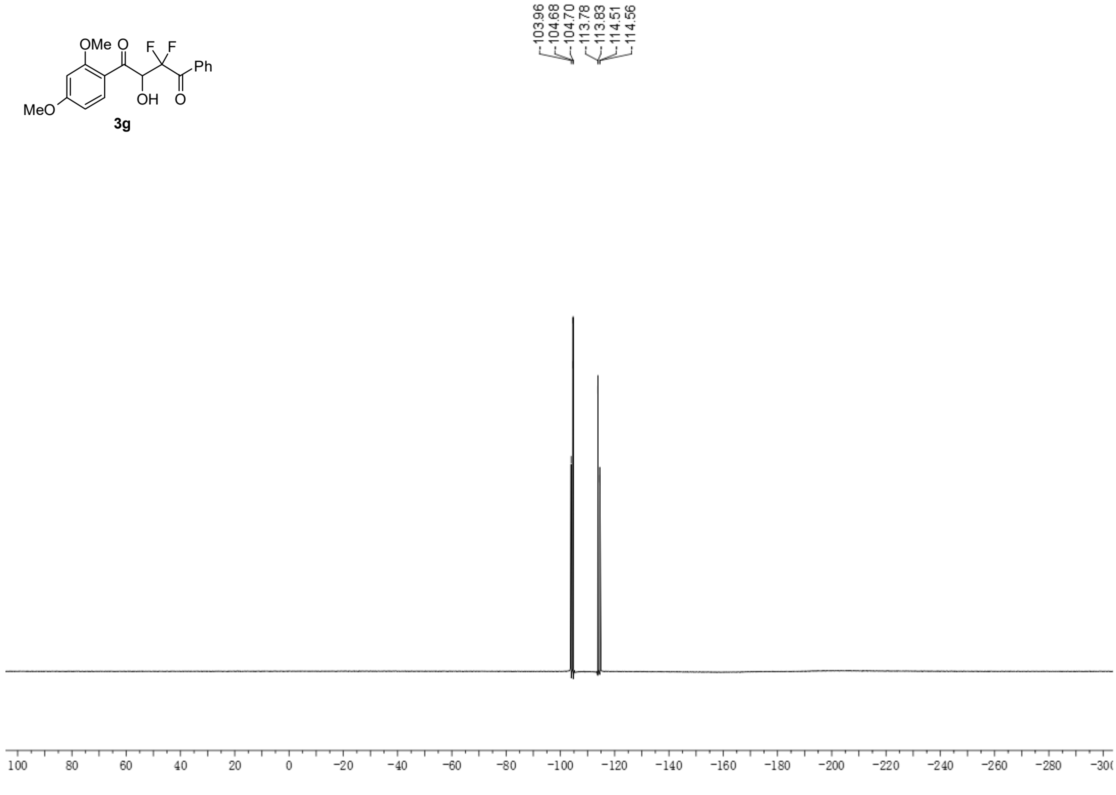

^{13}C NMR (100 MHz, CDCl$_3$) of **3g**

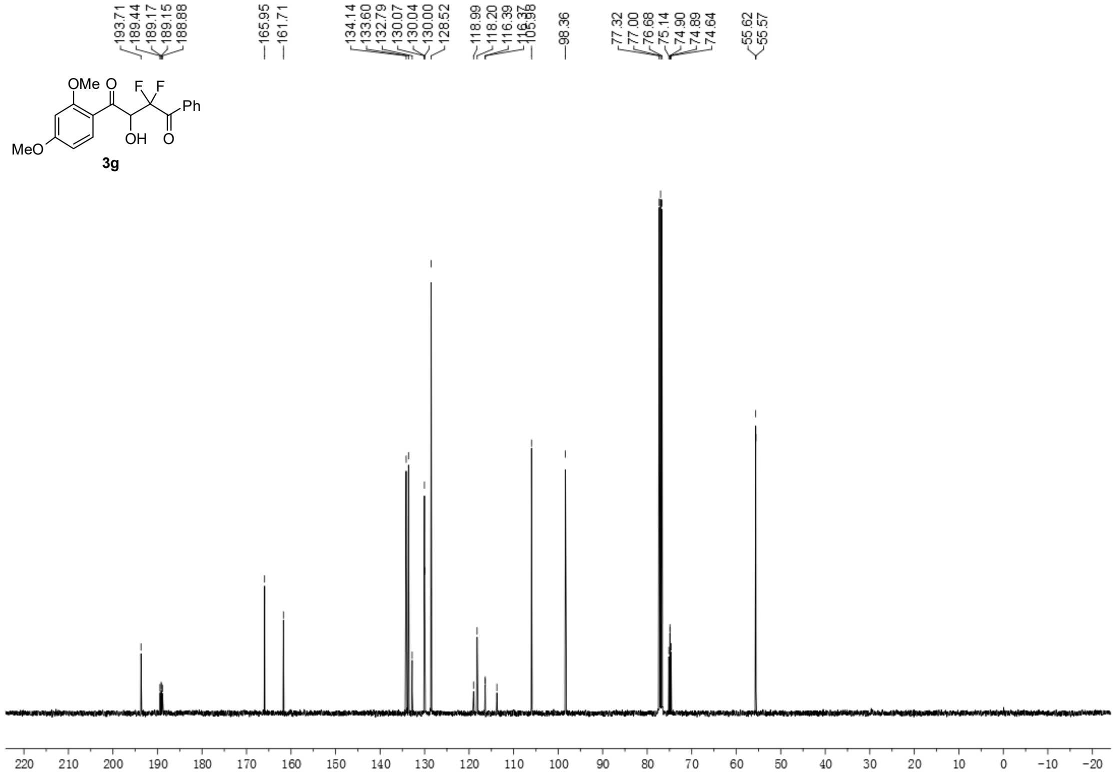

^{1}H NMR (400 MHz, CDCl$_3$) of **3h**

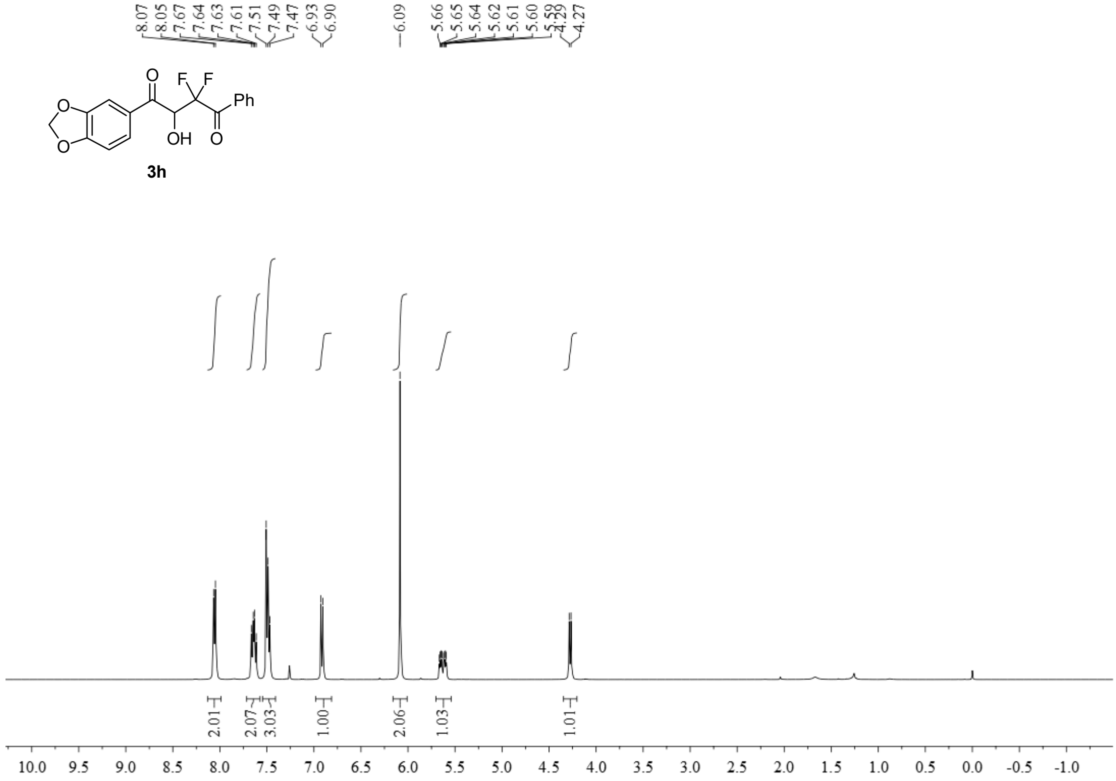

^{19}F NMR (376 MHz, $CDCl_3$) of **3h**

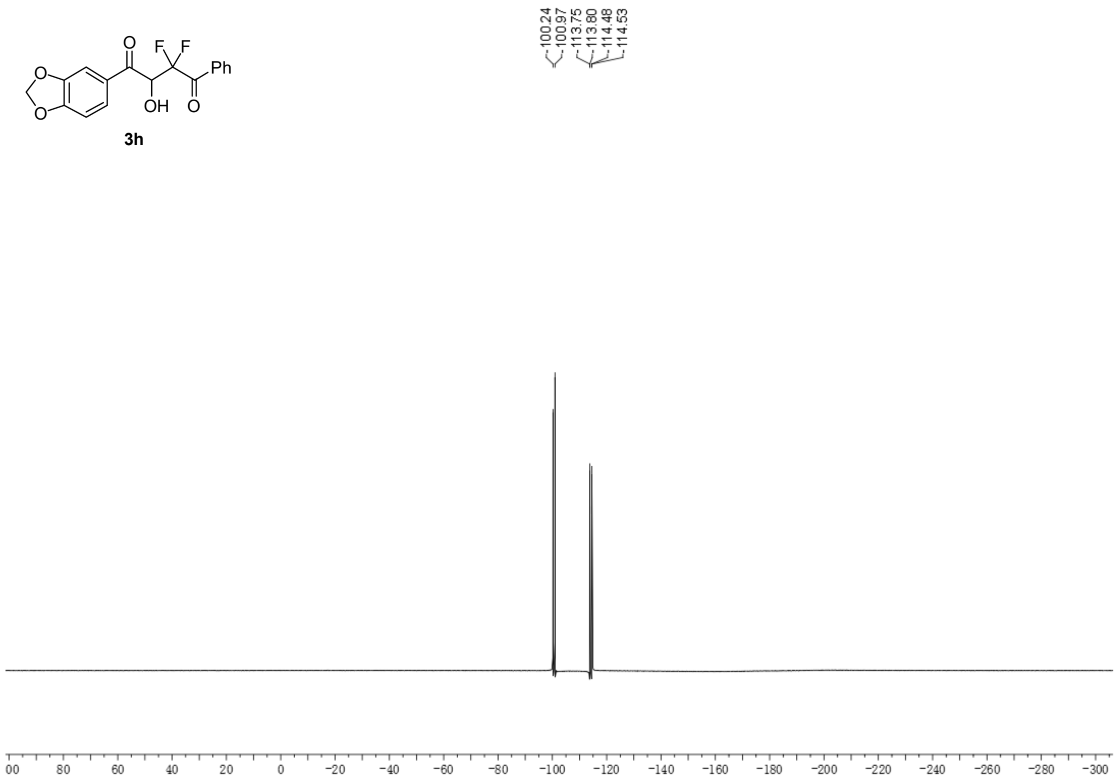

^{13}C NMR (100 MHz, $CDCl_3$) of **3h**

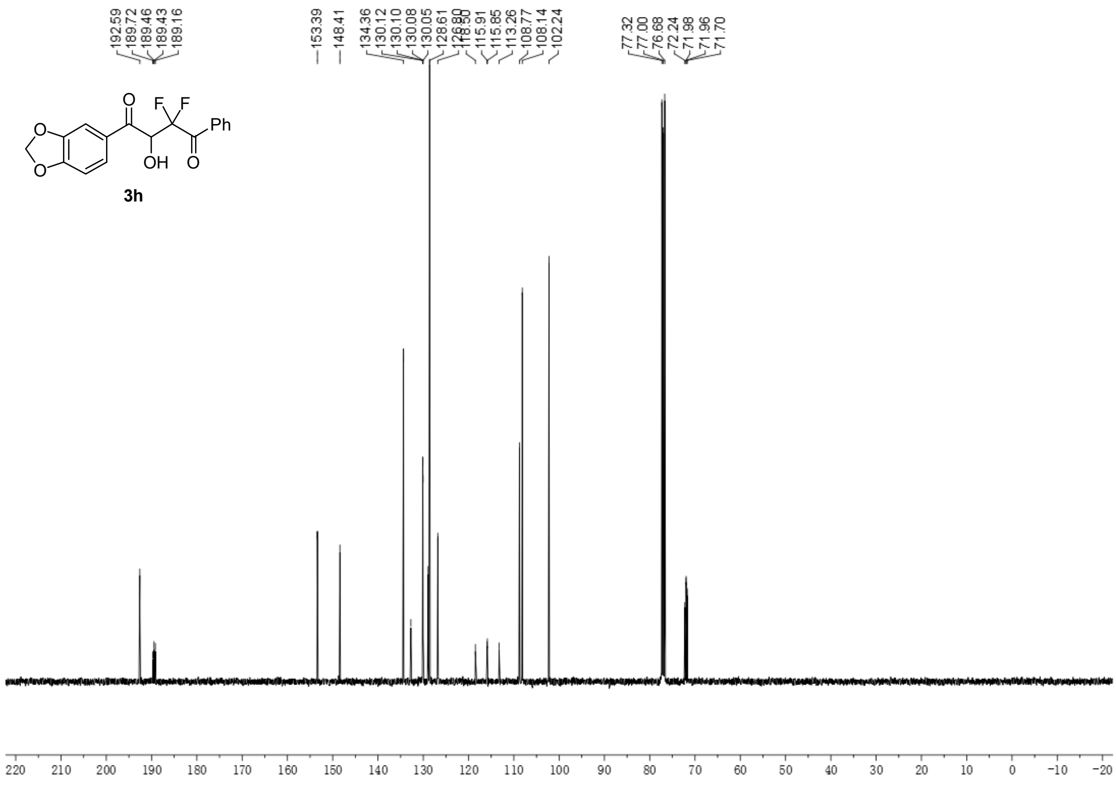

^{1}H NMR (400 MHz, $CDCl_3$) of **3i**

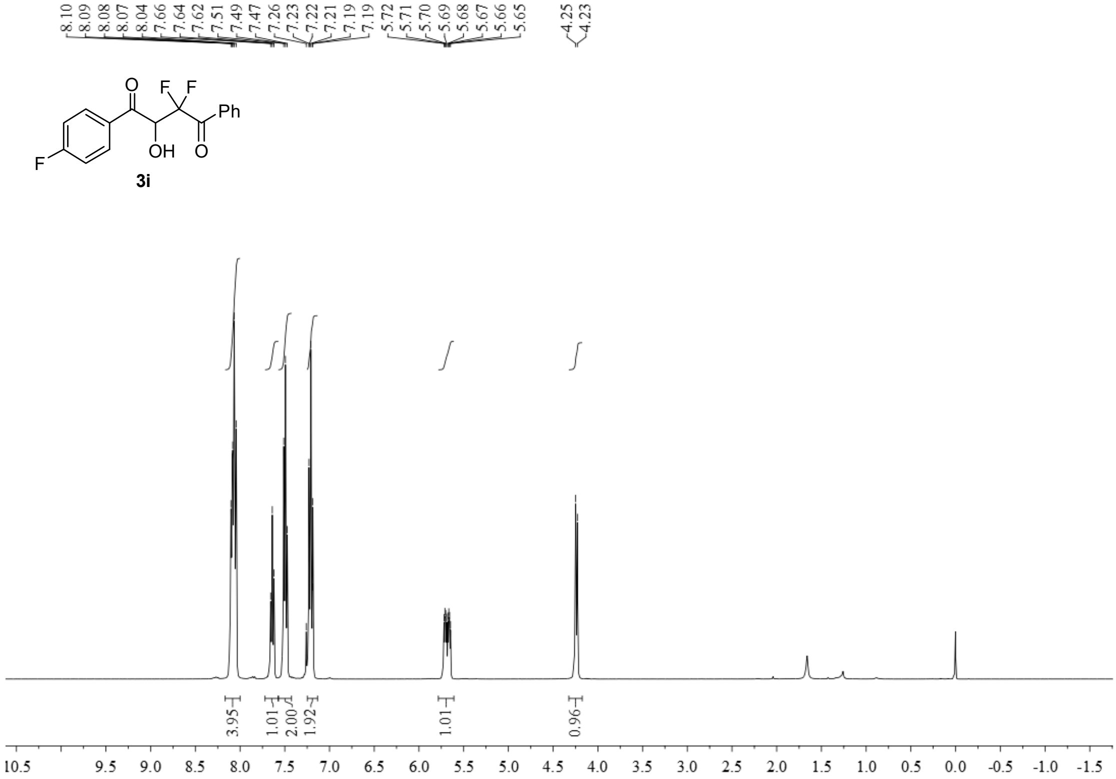

^{19}F NMR (376 MHz, $CDCl_3$) of **3i**

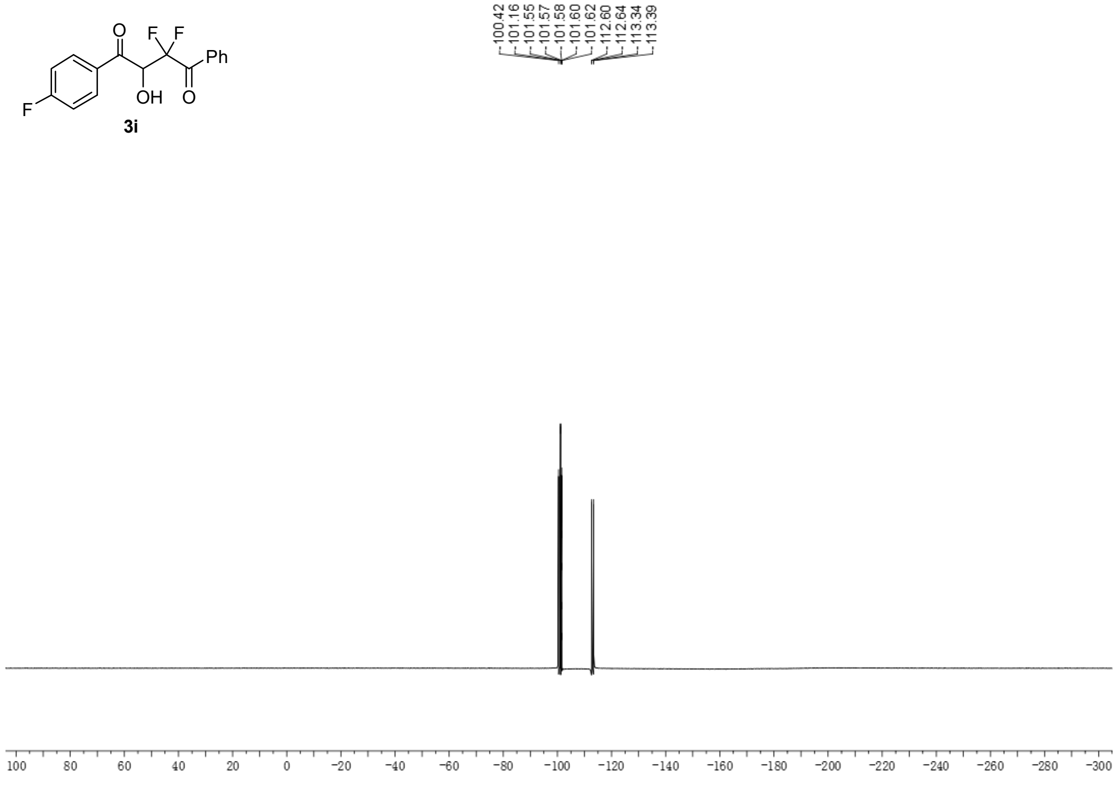

^{13}C NMR (100 MHz, $CDCl_3$) of **3i**

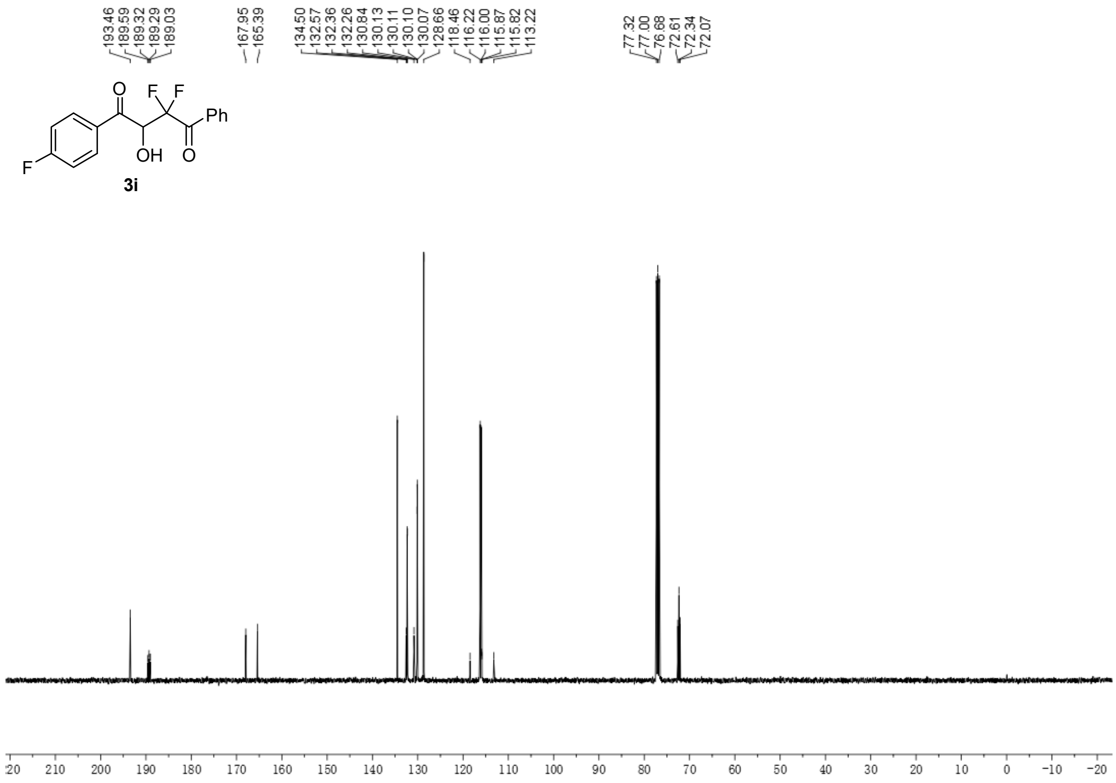

^{1}H NMR (400 MHz, $CDCl_3$) of **3j**

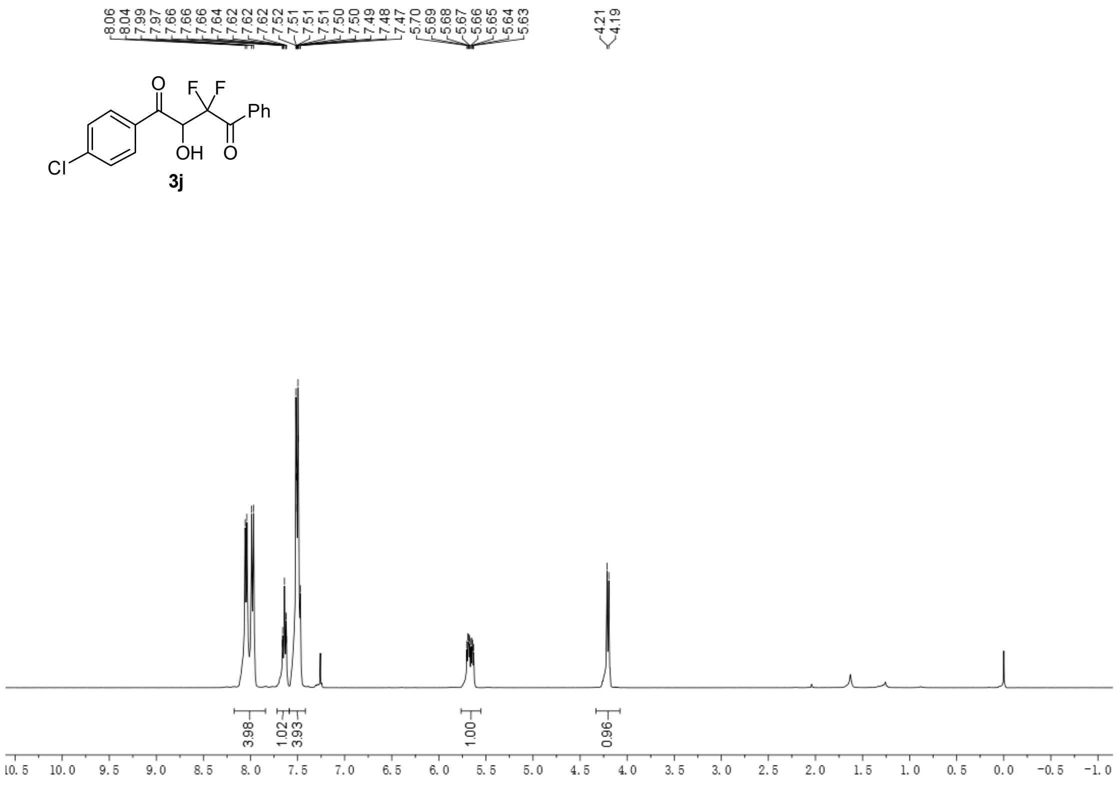

^{19}F NMR (376 MHz, $CDCl_3$) of **3j**

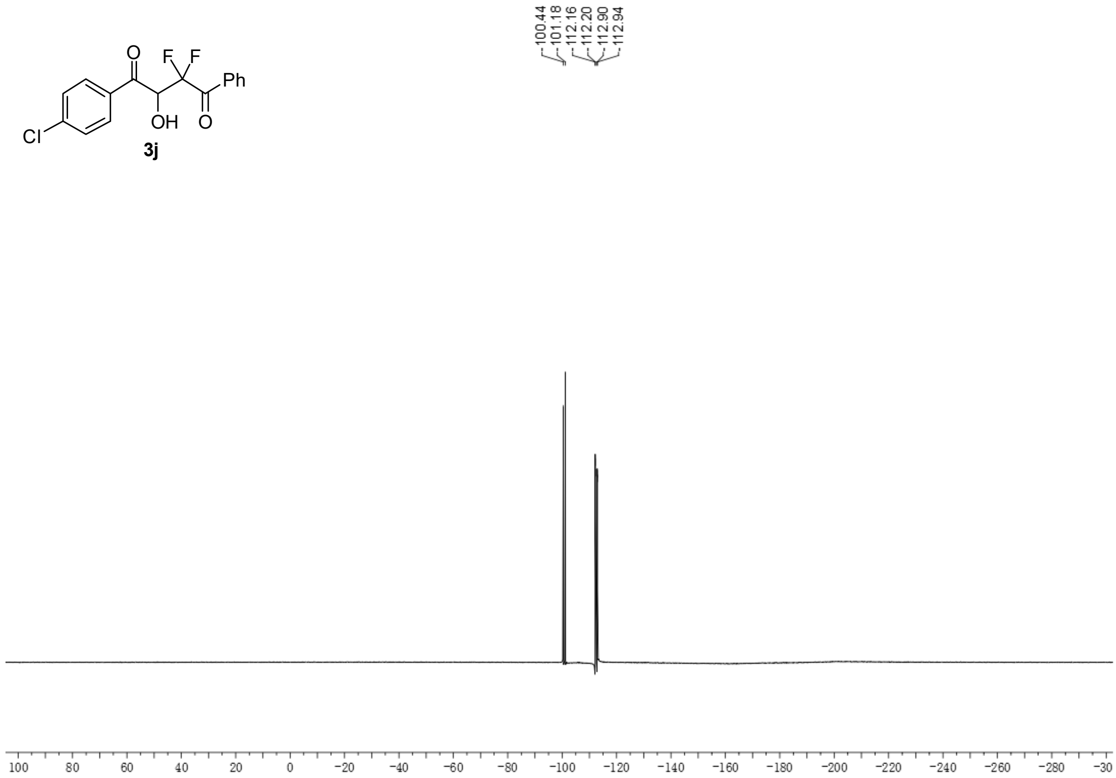

^{13}C NMR (100 MHz, $CDCl_3$) of **3j**

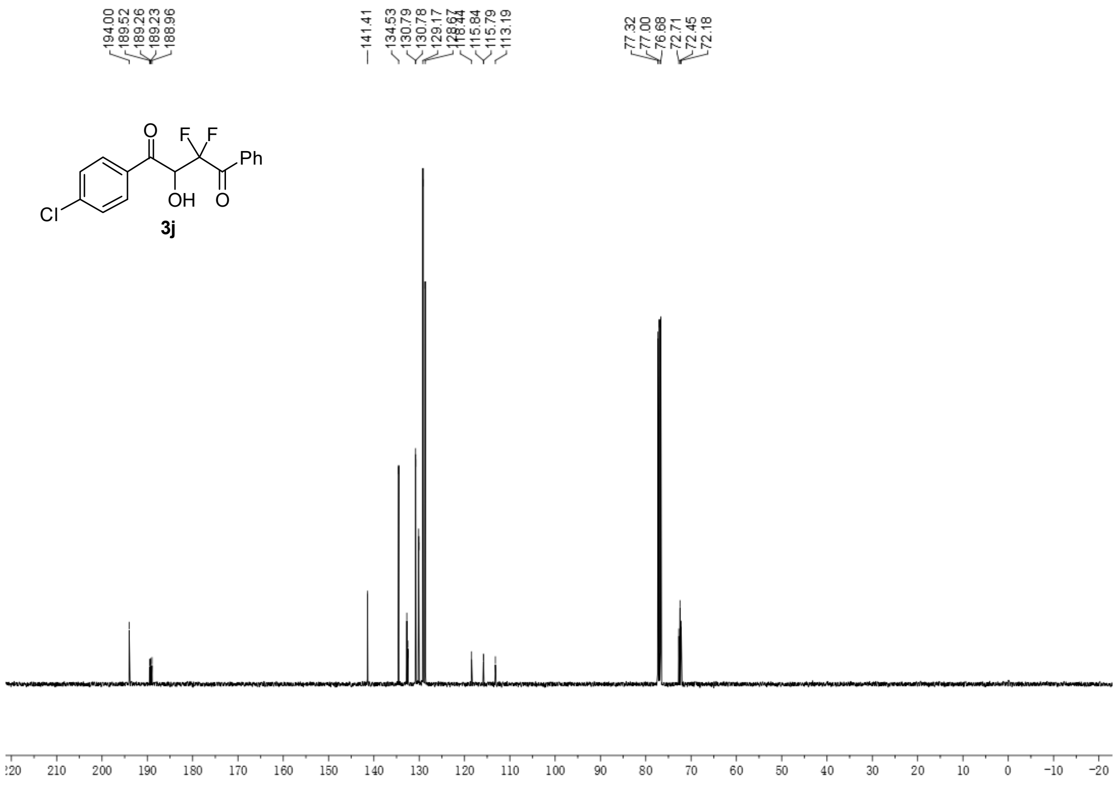

^{1}H NMR (400 MHz, $CDCl_3$) of **3k**

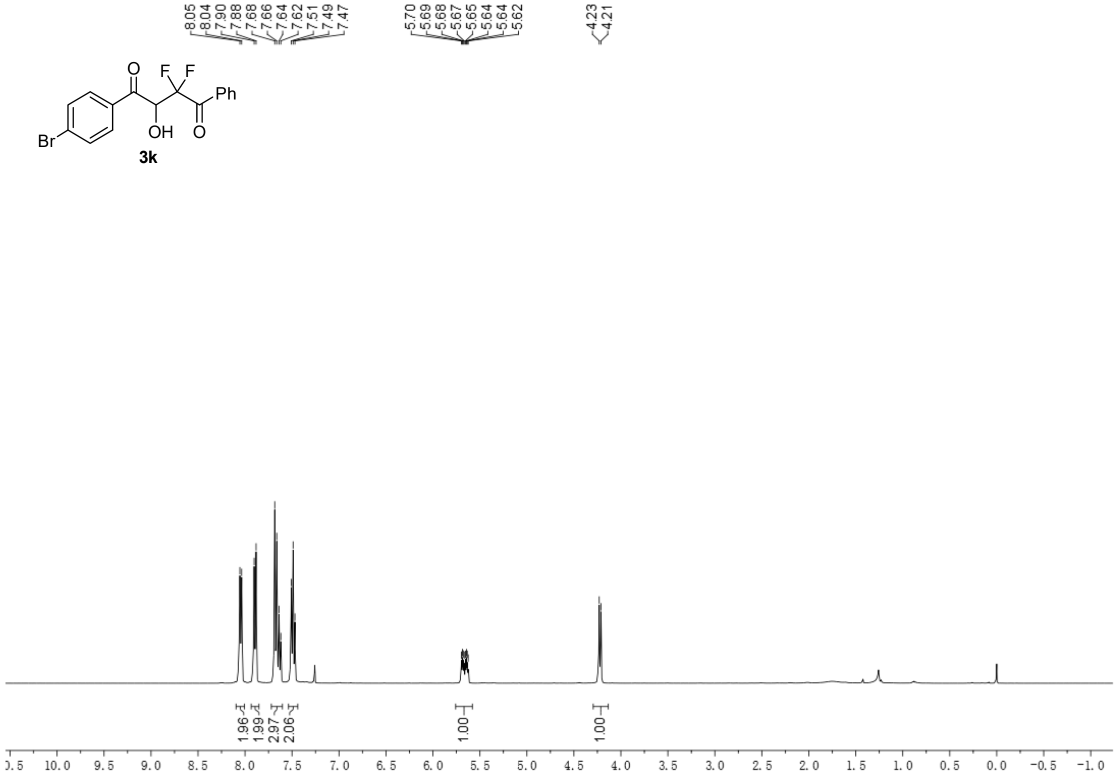

^{19}F NMR (376 MHz, $CDCl_3$) of **3k**

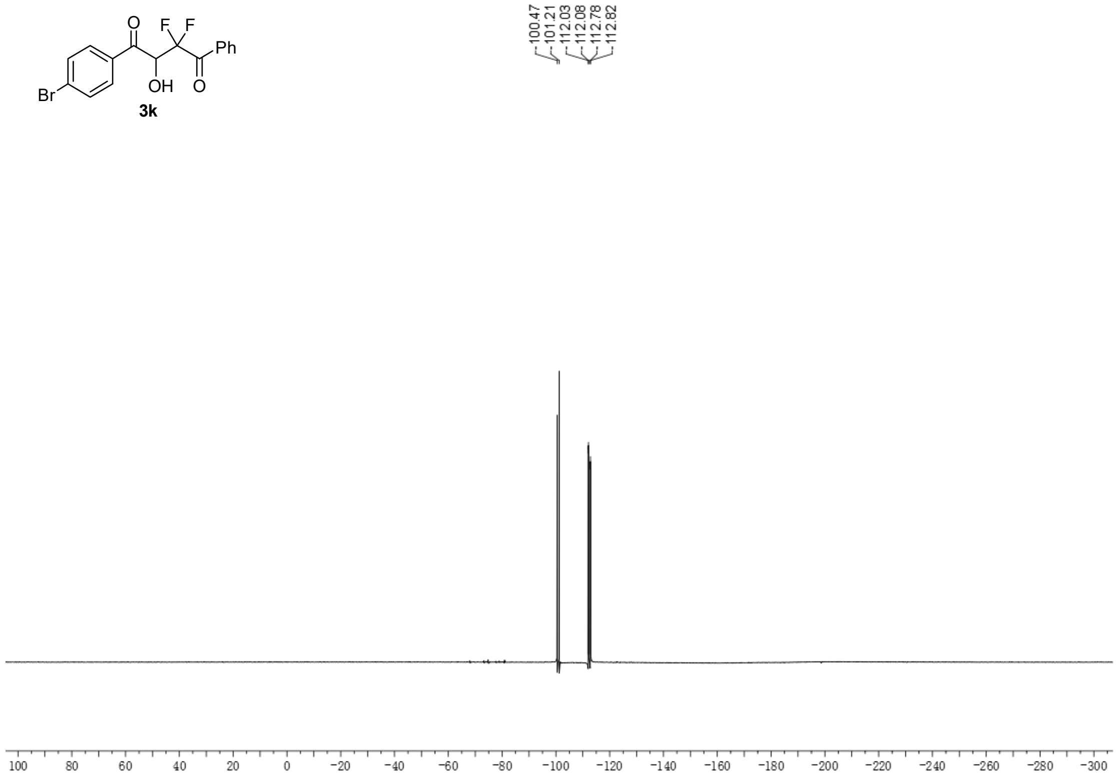

^{13}C NMR (100 MHz, $CDCl_3$) of **3k**

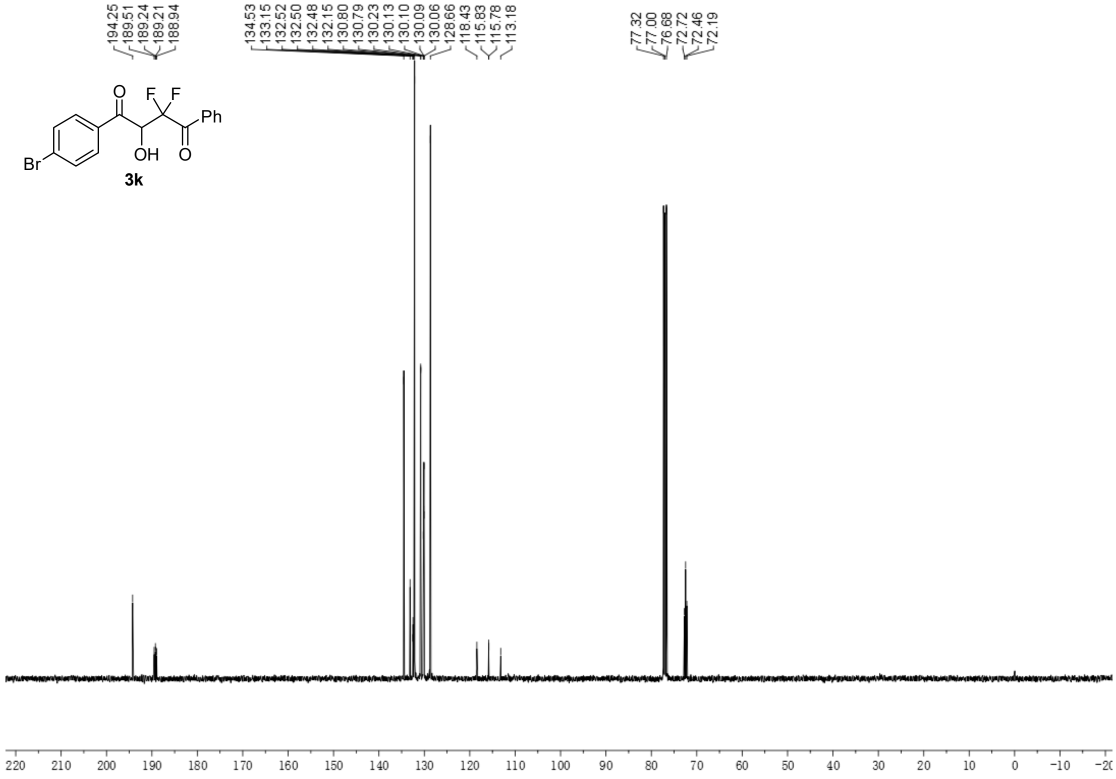

^{1}H NMR (400 MHz, $CDCl_3$) of **3l**

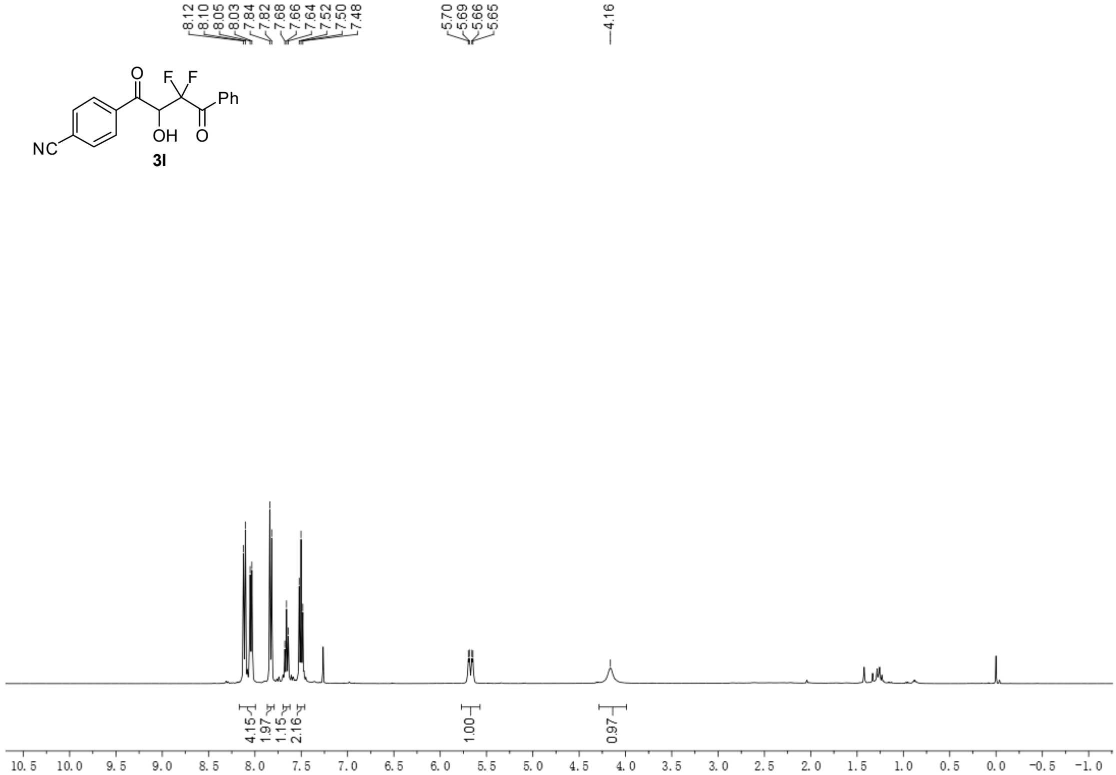

^{19}F NMR (376 MHz, $CDCl_3$) of **3l**

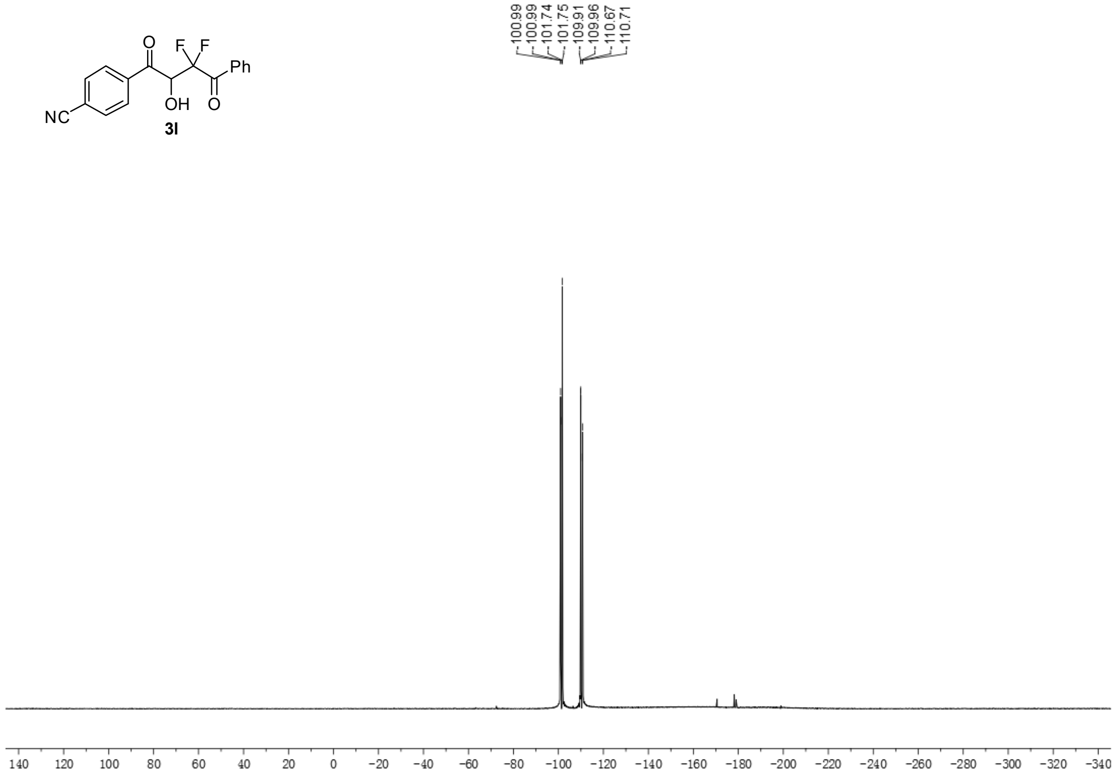

^{13}C NMR (100 MHz, $CDCl_3$) of **3l**

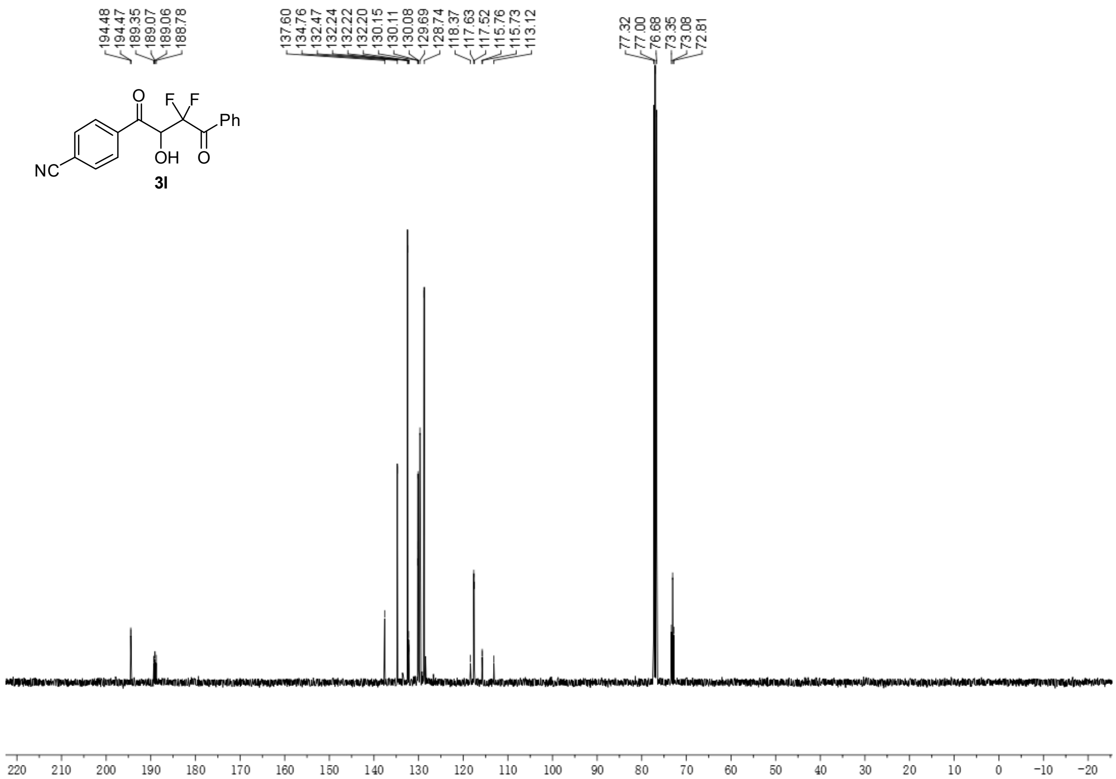

^{1}H NMR (400 MHz, $CDCl_3$) of **3m**

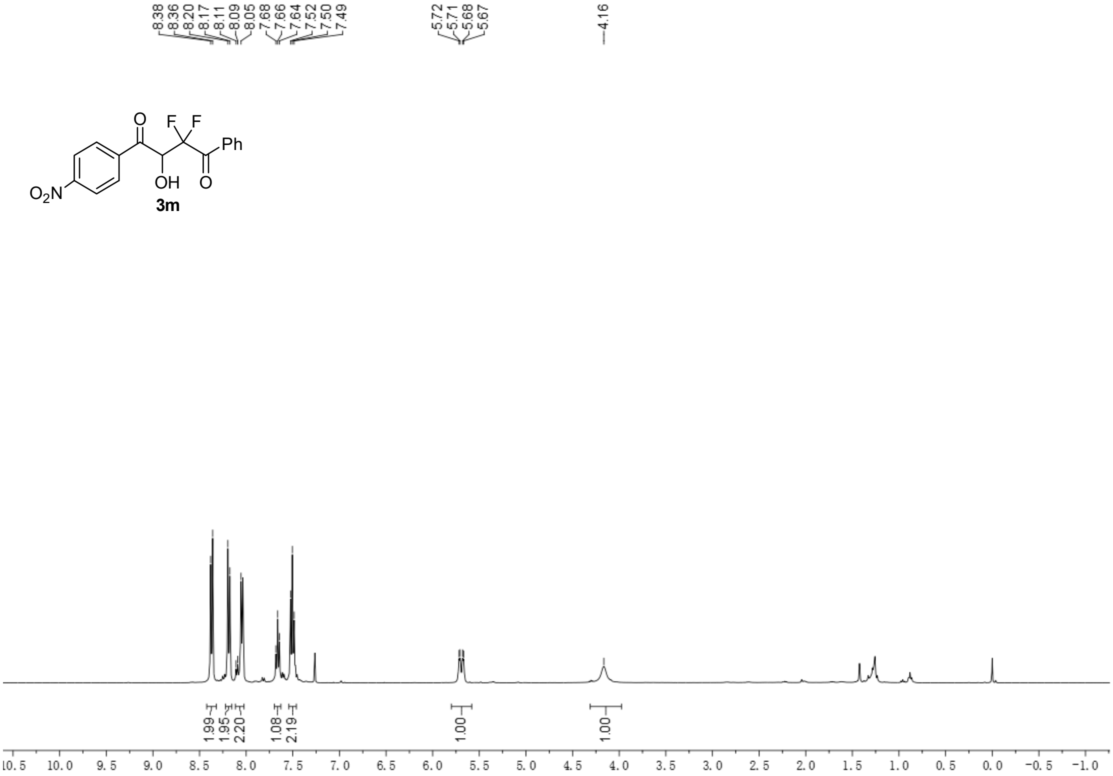

^{19}F NMR (376 MHz, $CDCl_3$) of **3m**

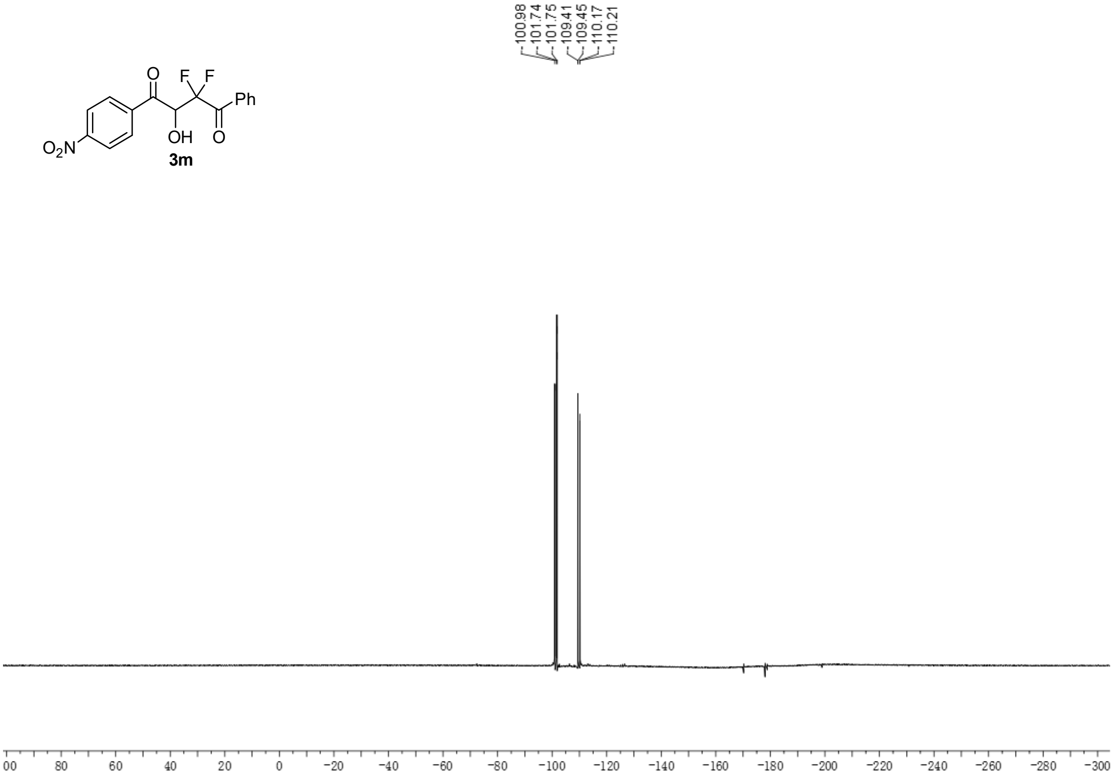

^{13}C NMR (100 MHz, $CDCl_3$) of **3m**

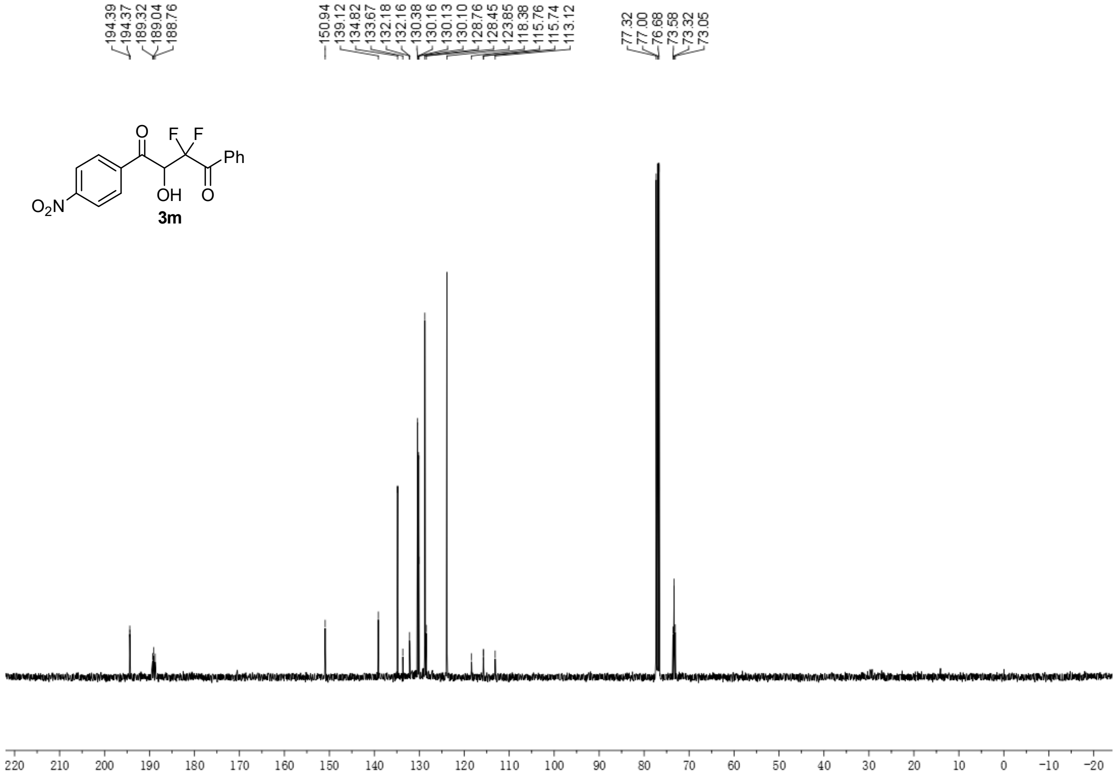

^{1}H NMR (400 MHz, $CDCl_3$) of **3n**

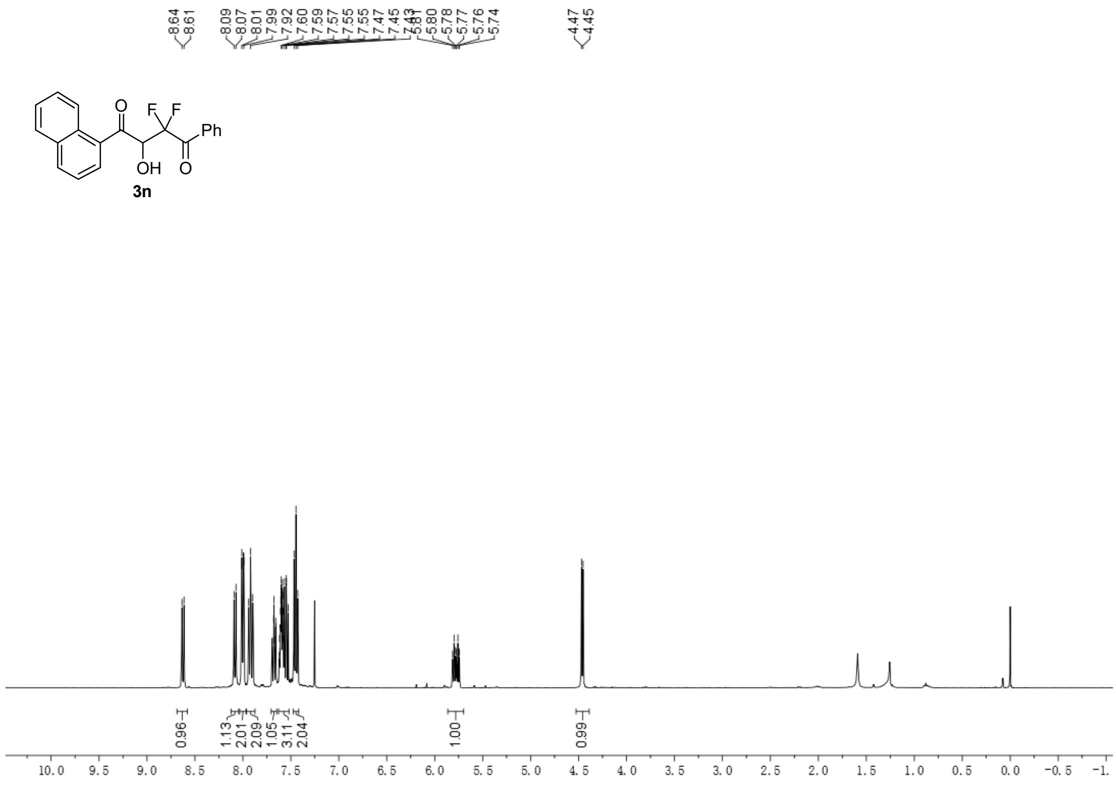

^{19}F NMR (376 MHz, $CDCl_3$) of **3n**

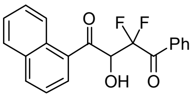

3n

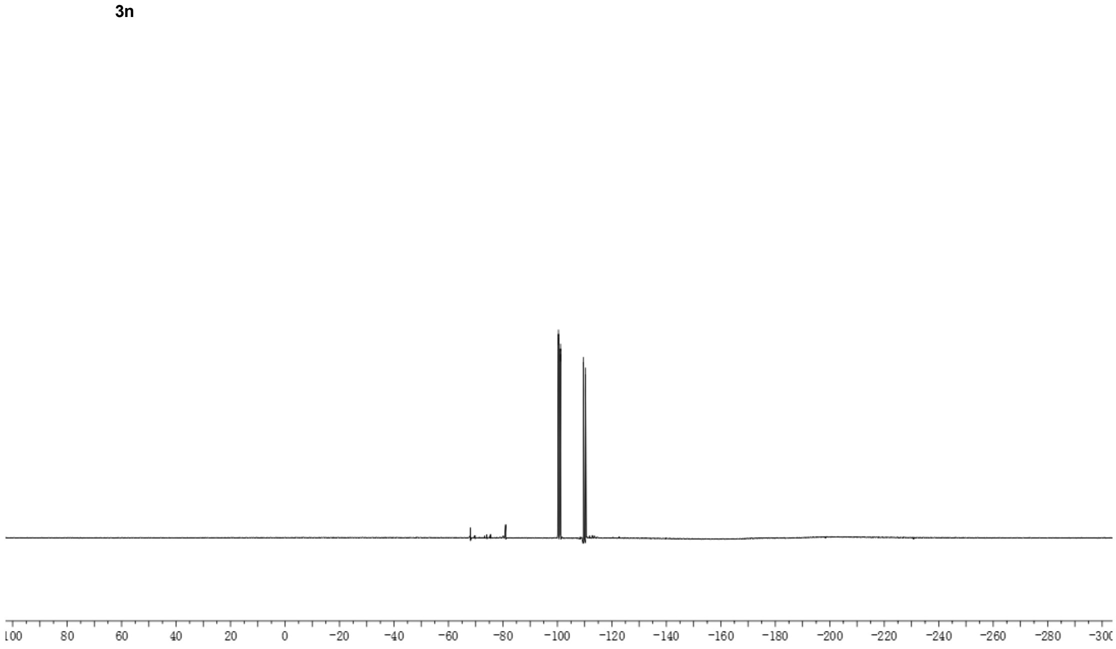

^{13}C NMR (100 MHz, $CDCl_3$) of **3n**

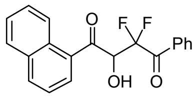

3n

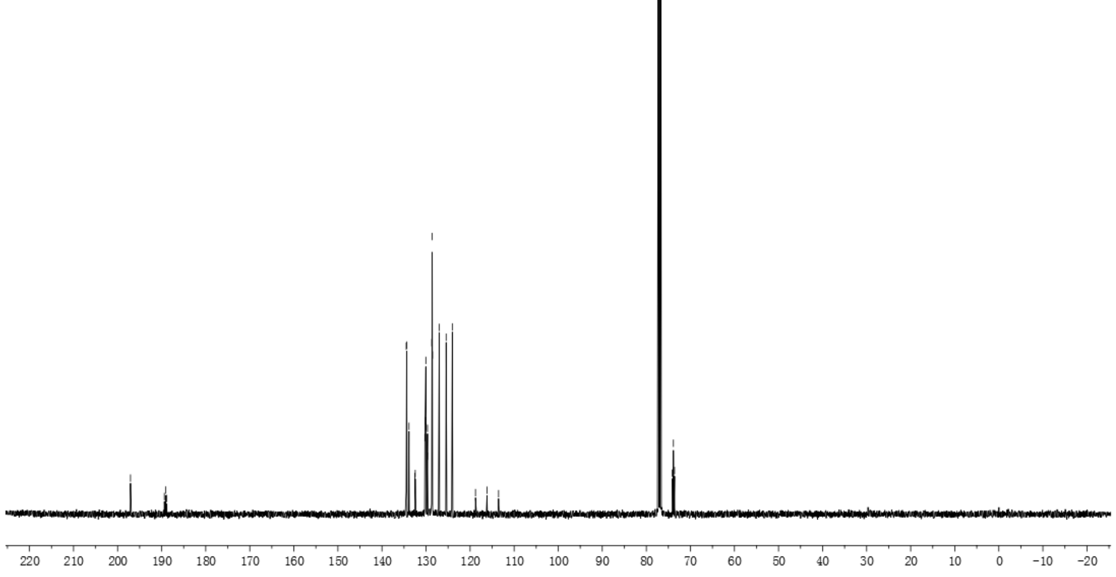

^{1}H NMR (400 MHz, $CDCl_3$) of **3o**

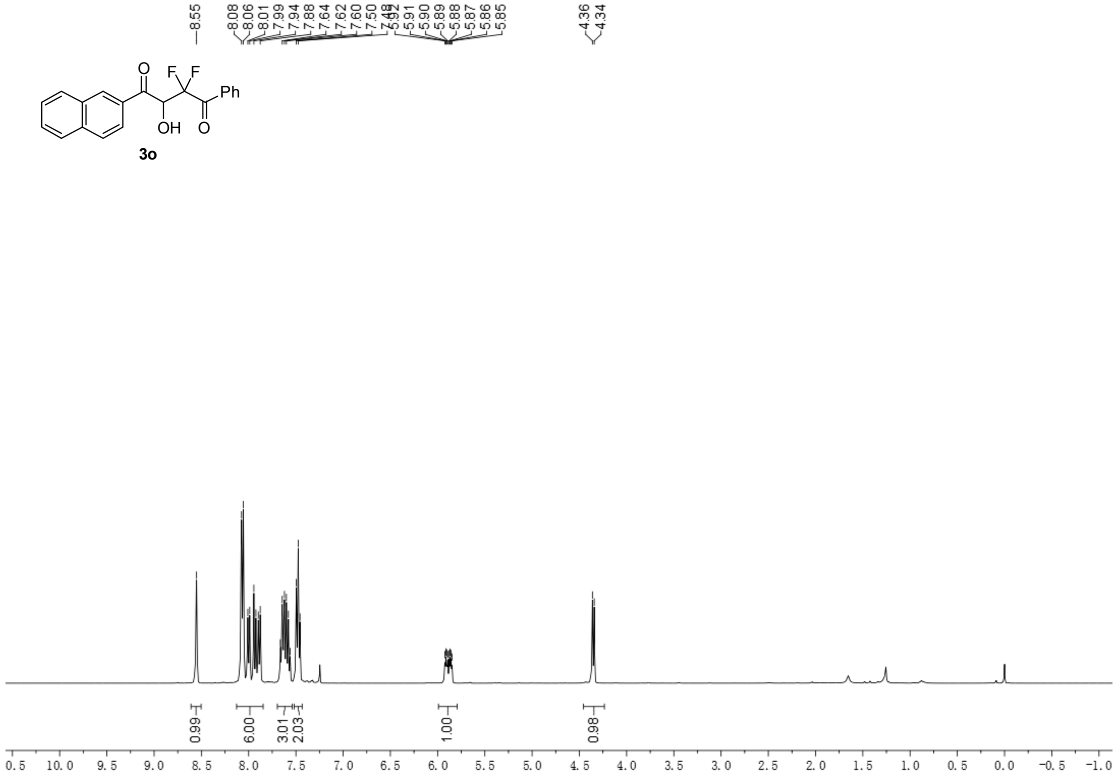

^{19}F NMR (376 MHz, $CDCl_3$) of **3o**

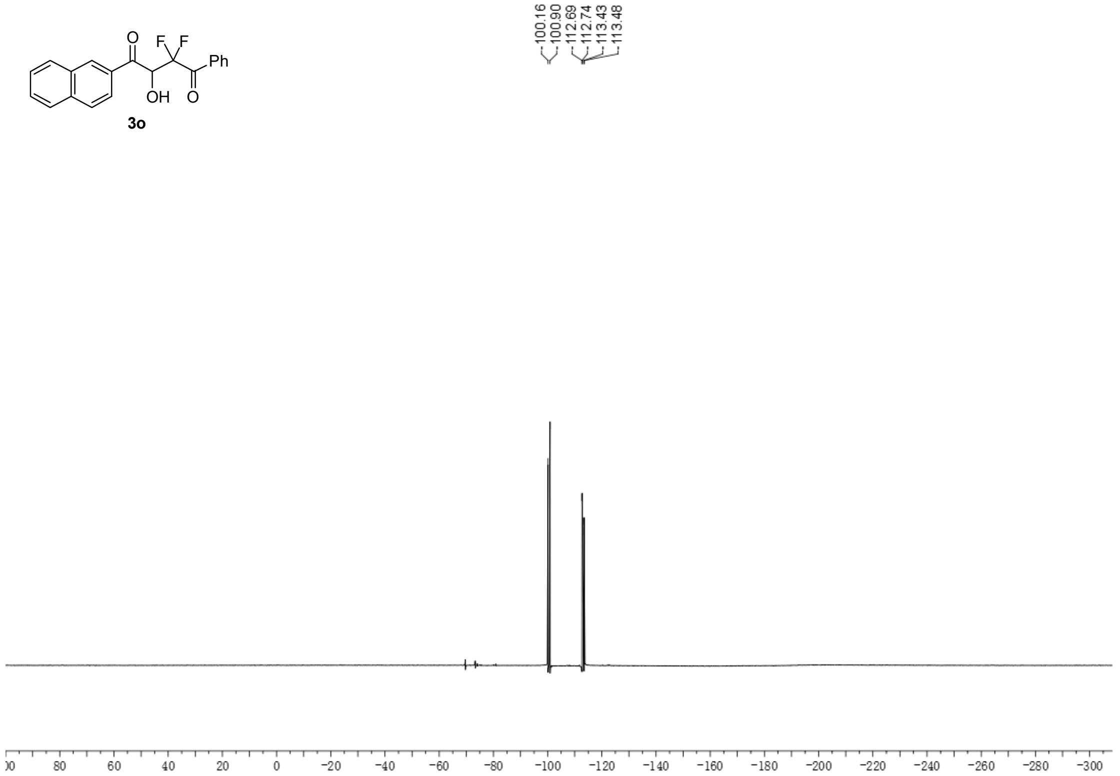

^{13}C NMR (100 MHz, $CDCl_3$) of **3o**

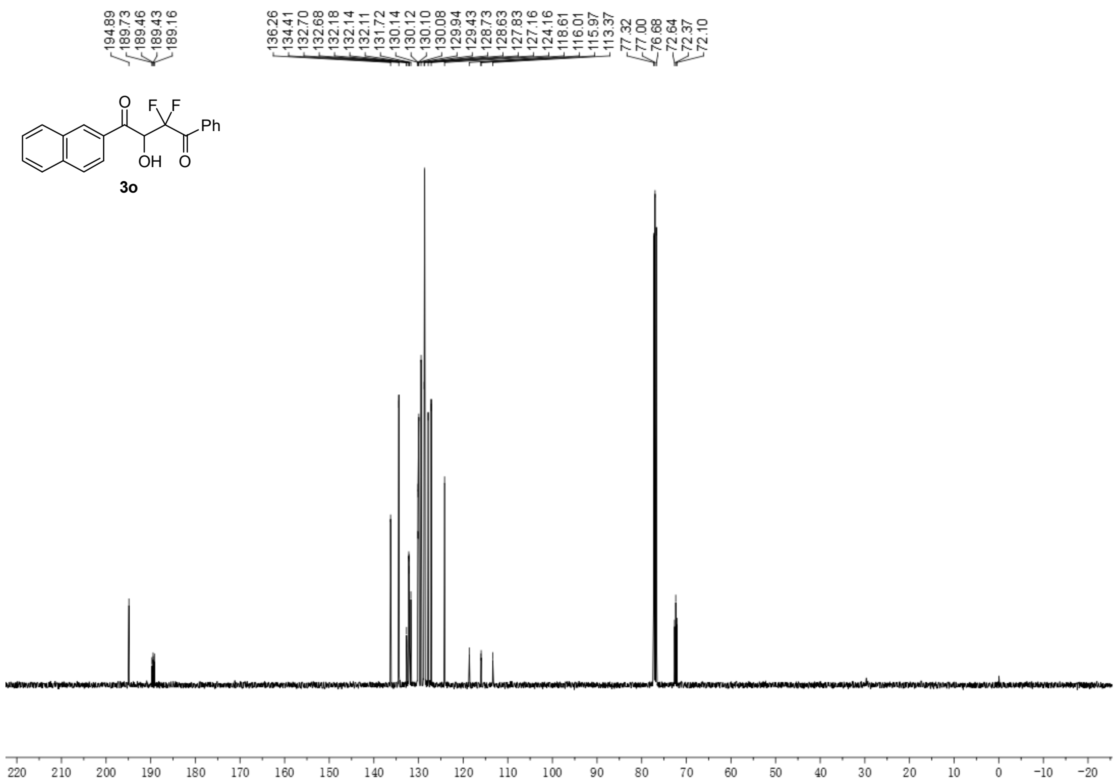

^{1}H NMR (400 MHz, $CDCl_3$) of **3p**

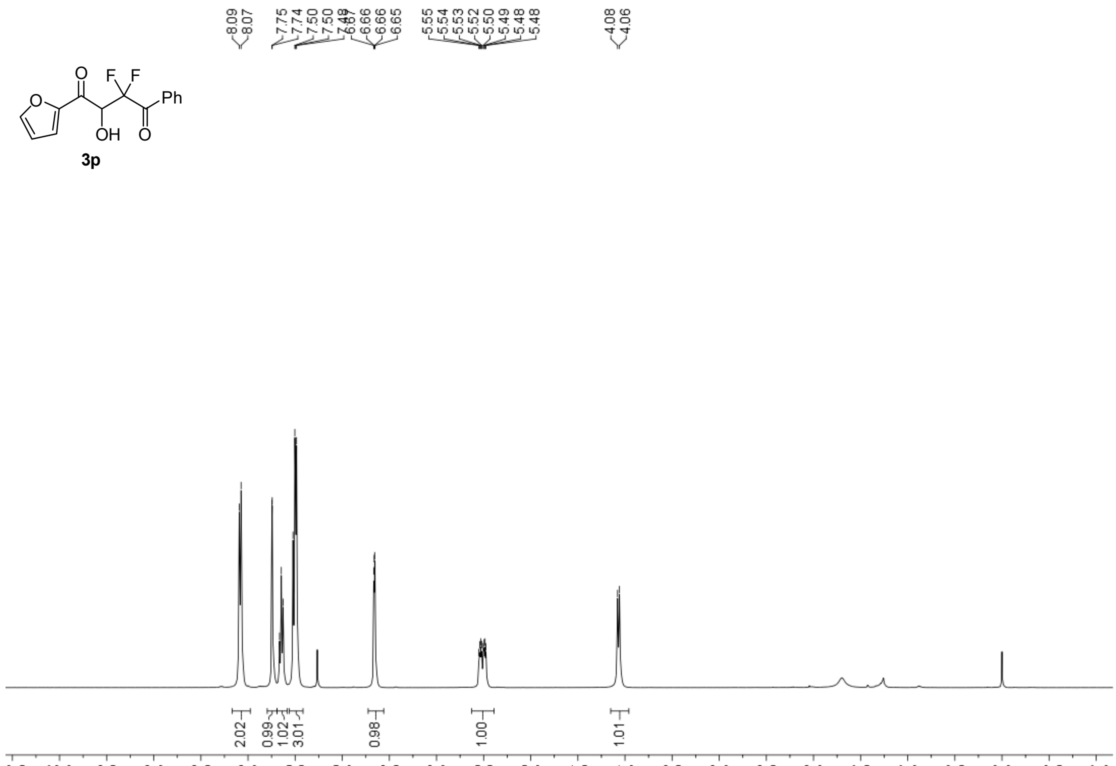

^{19}F NMR (376 MHz, CDCl$_3$) of **3p**

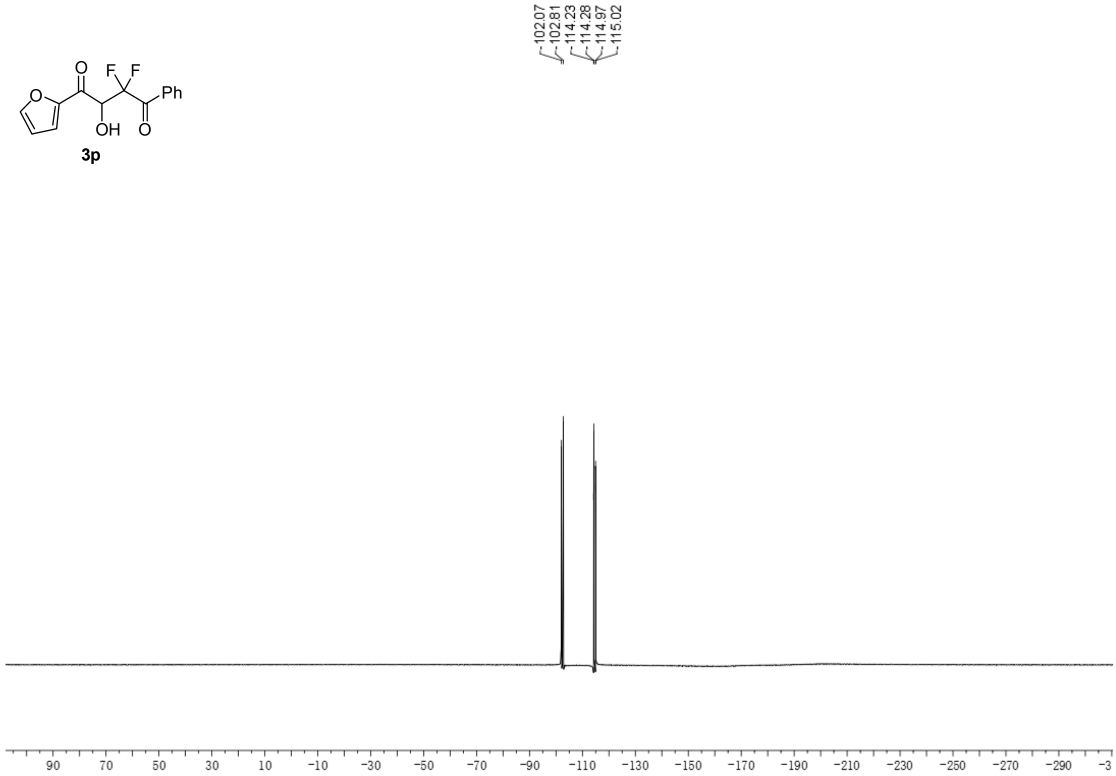

^{13}C NMR (100 MHz, CDCl$_3$) of **3p**

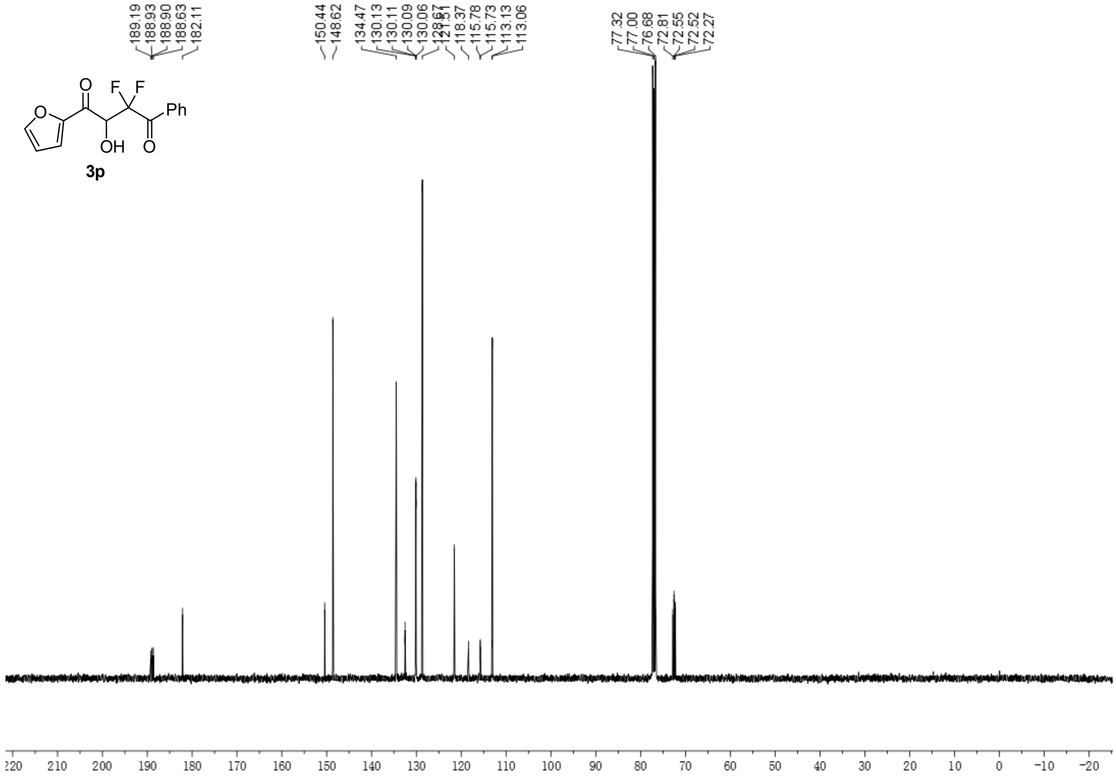

^{1}H NMR (400 MHz, $CDCl_3$) of **3q**

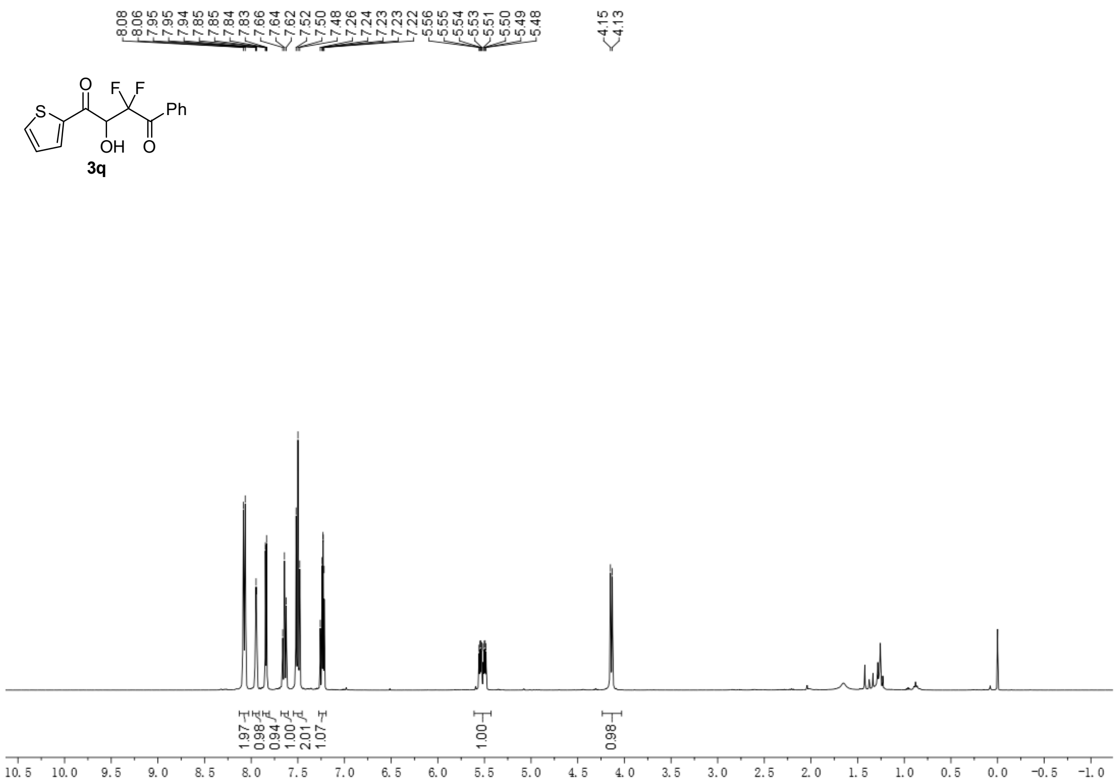

^{19}F NMR (376 MHz, $CDCl_3$) of **3q**

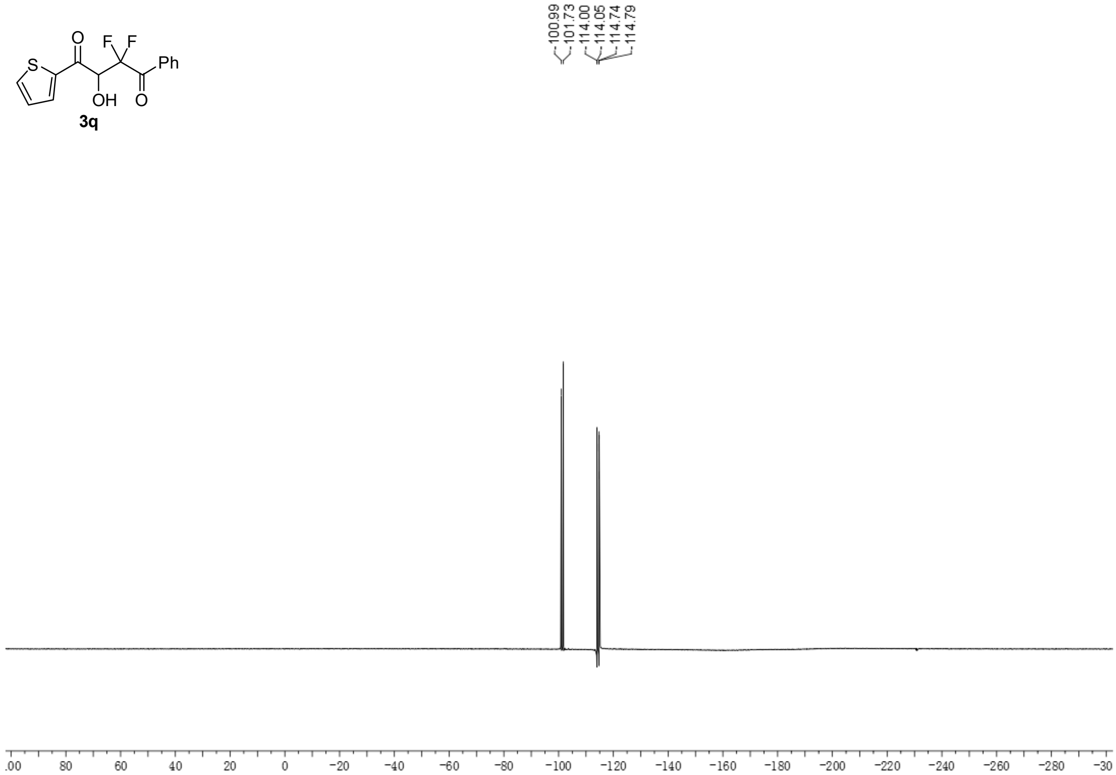

^{13}C NMR (100 MHz, $CDCl_3$) of **3q**

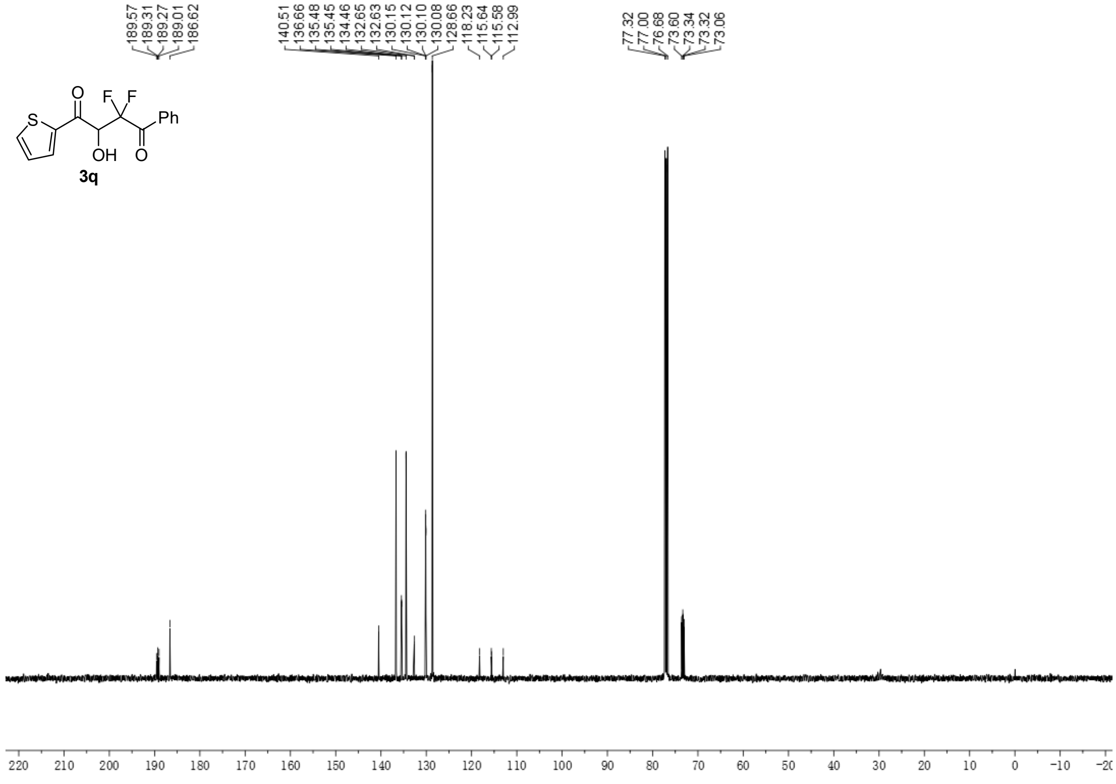

^{1}H NMR (400 MHz, $CDCl_3$) of **3r**

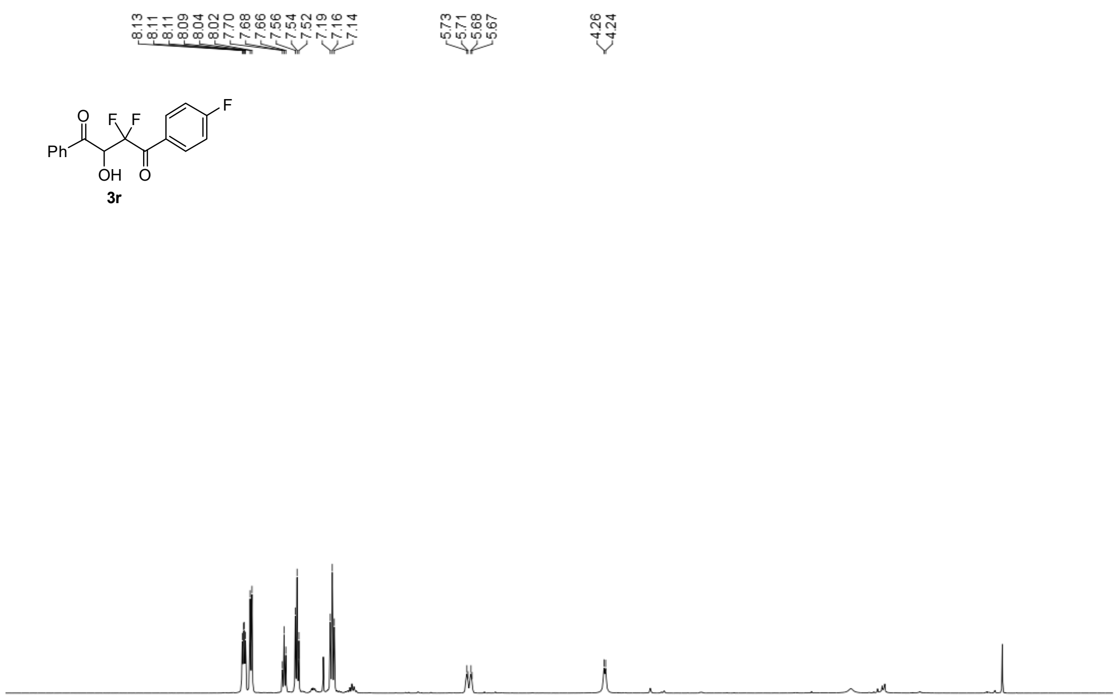

^{19}F NMR (376 MHz, $CDCl_3$) of **3r**

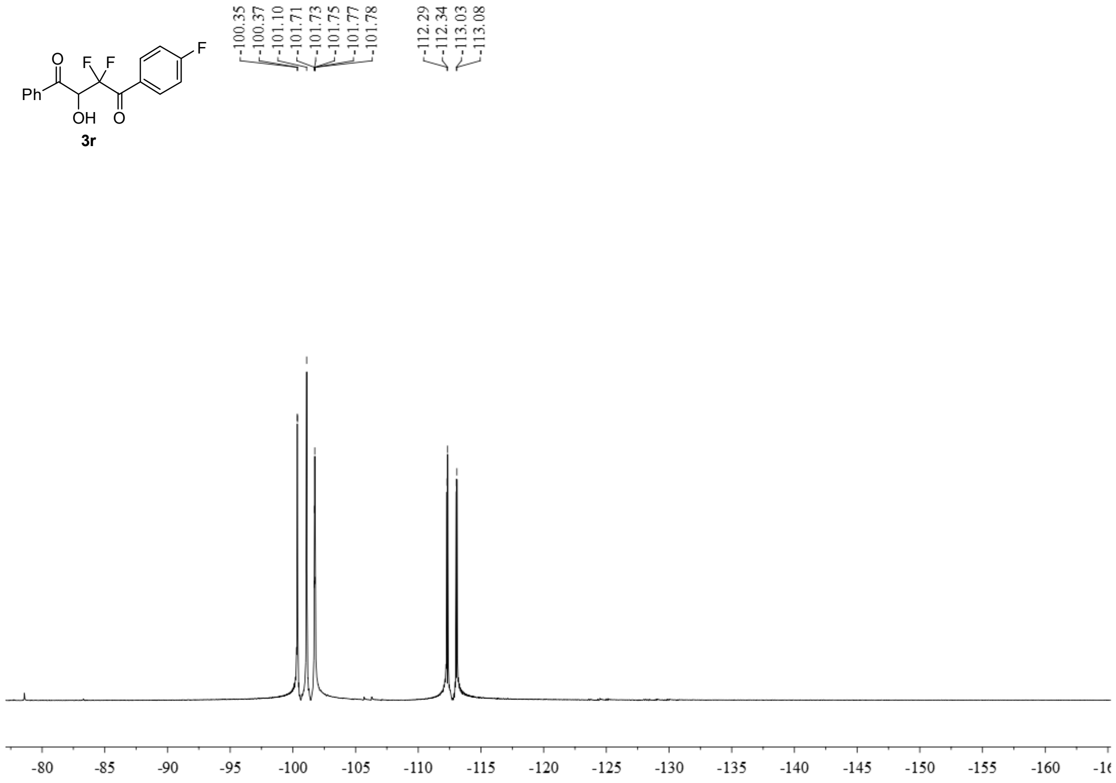

^{13}C NMR (100 MHz, $CDCl_3$) of **3r**

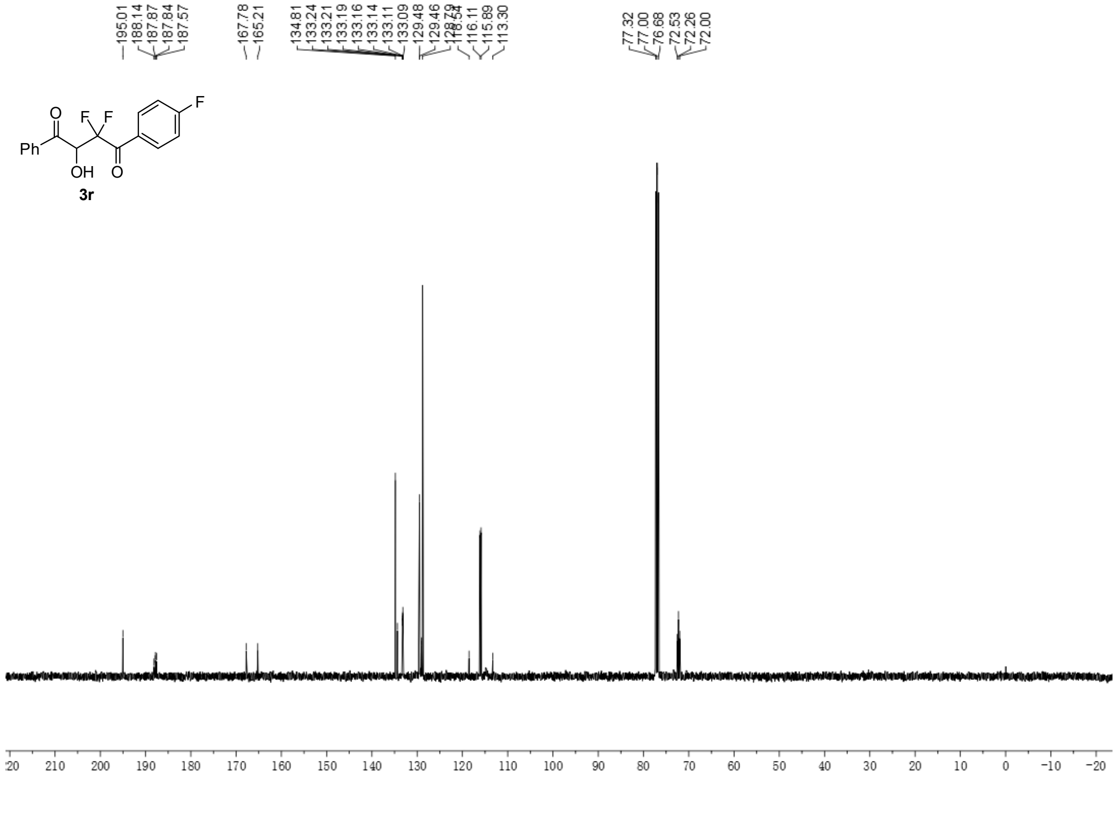

^{1}H NMR (400 MHz, $CDCl_3$) of **3s**

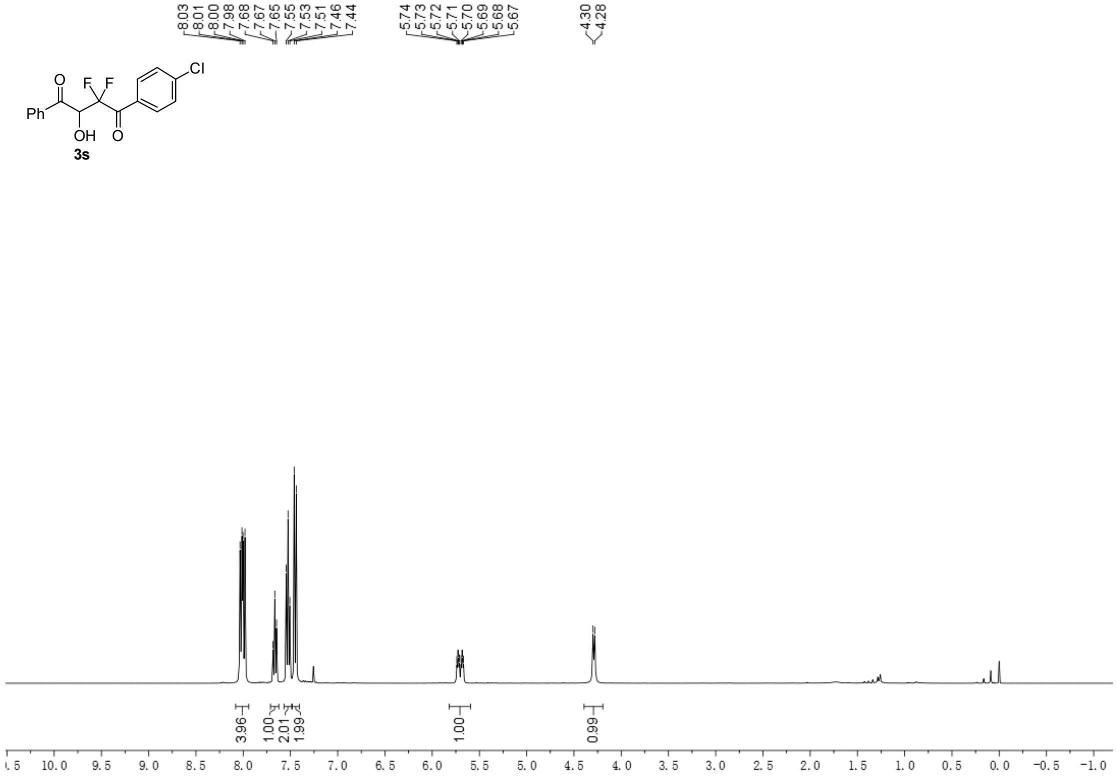

^{19}F NMR (376 MHz, $CDCl_3$) of **3s**

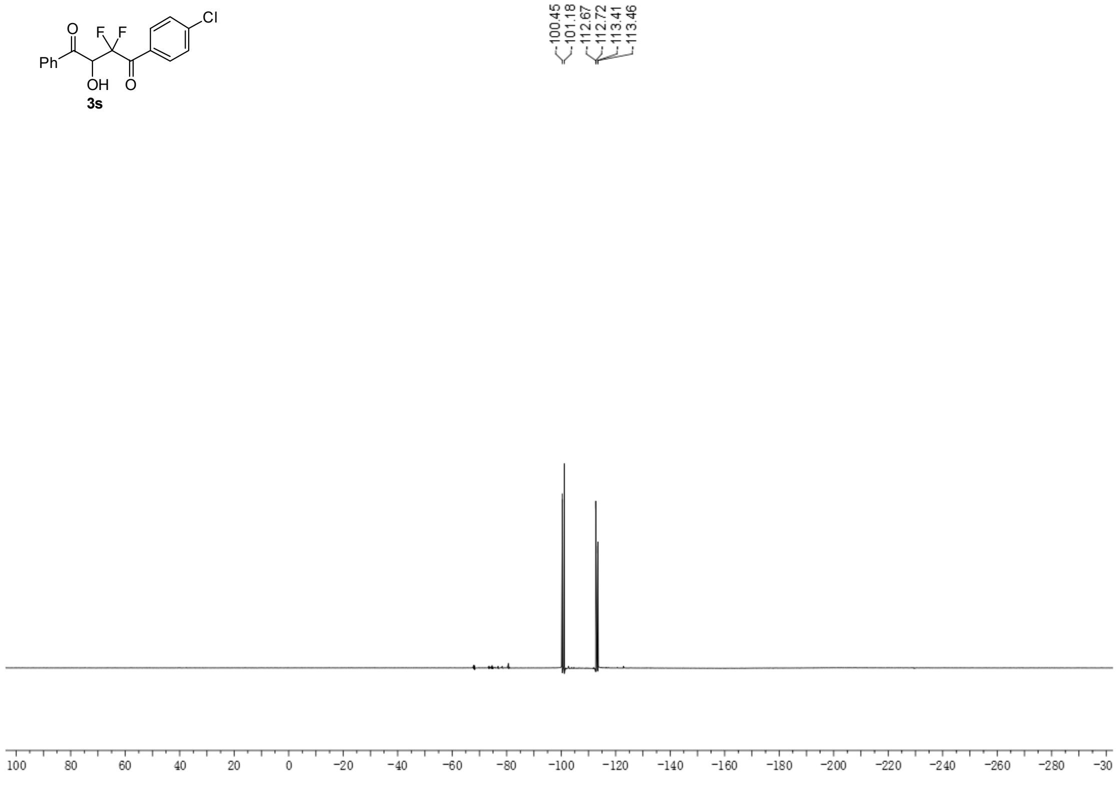

^{13}C NMR (100 MHz, $CDCl_3$) of **3s**

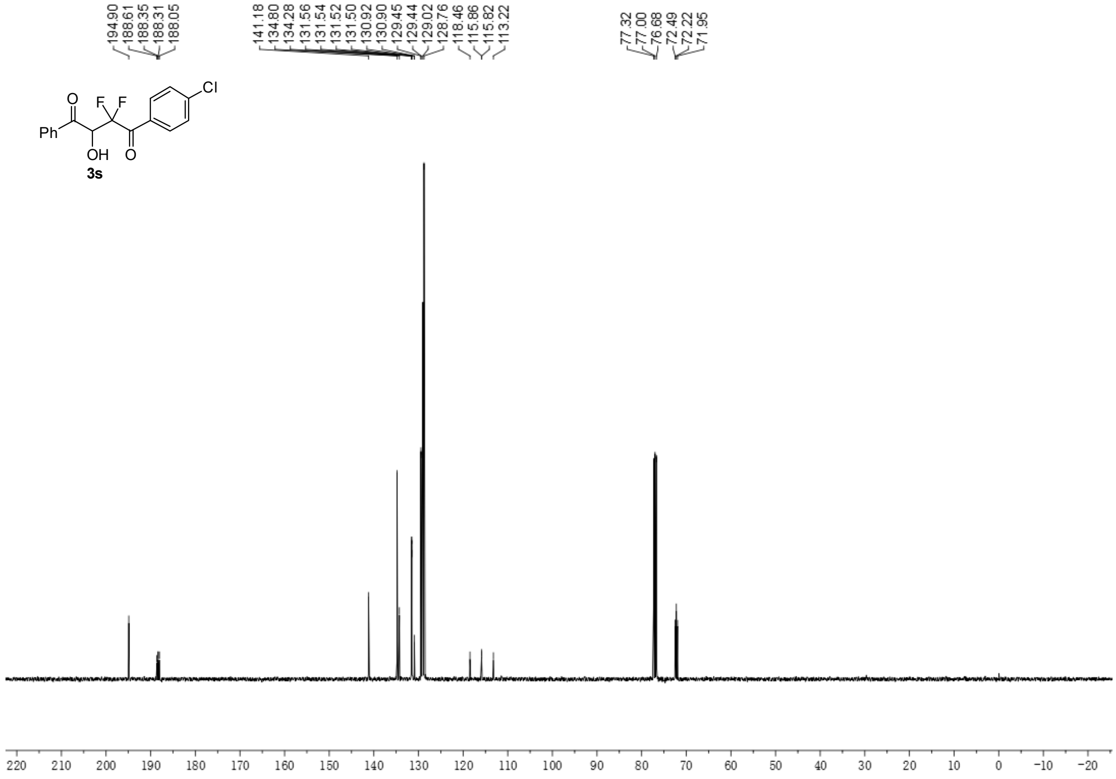

^{1}H NMR (400 MHz, $CDCl_3$) of **3t**

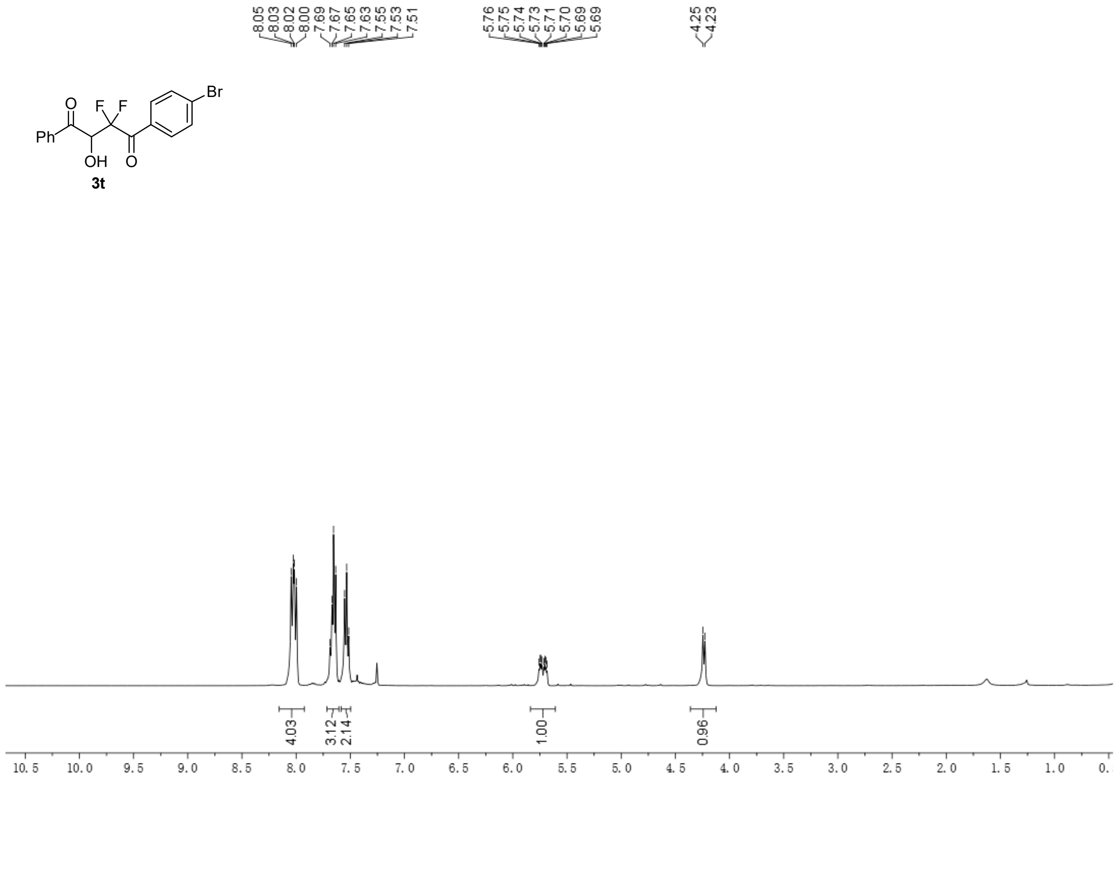

^{19}F NMR (376 MHz, $CDCl_3$) of **3t**

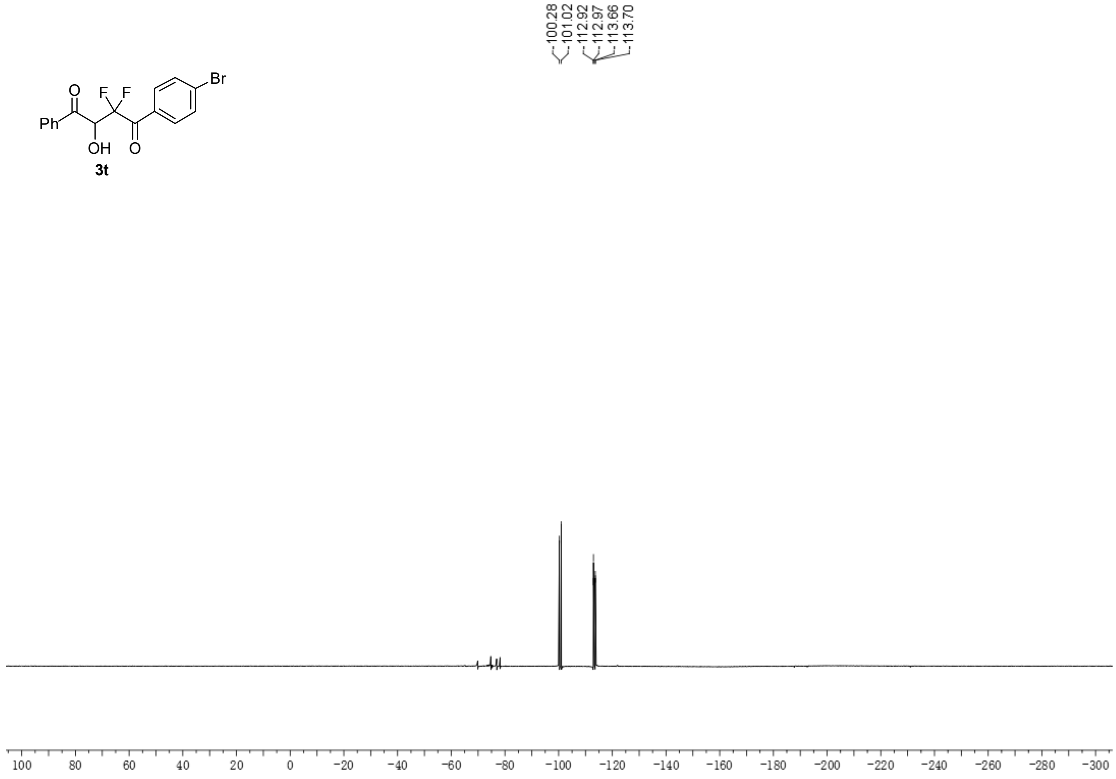

^{13}C NMR (100 MHz, $CDCl_3$) of **3t**

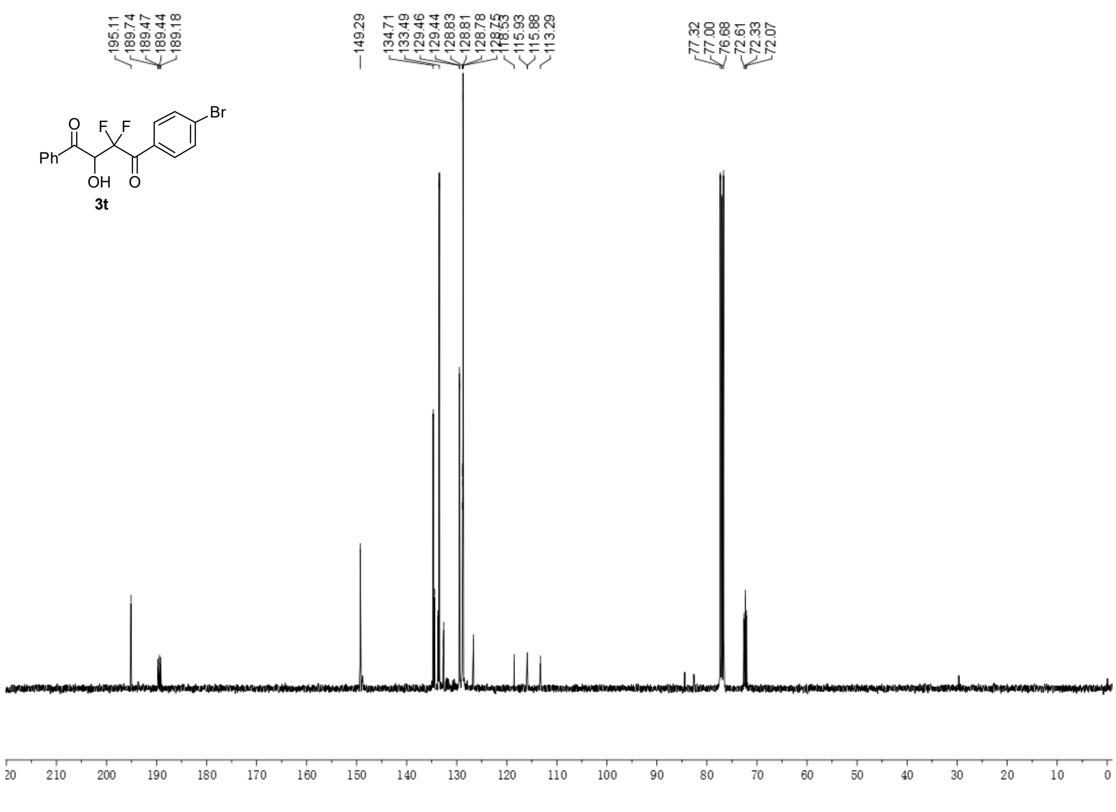

^{1}H NMR (400 MHz, $CDCl_3$) of **3u**

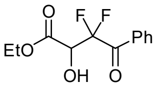

3u

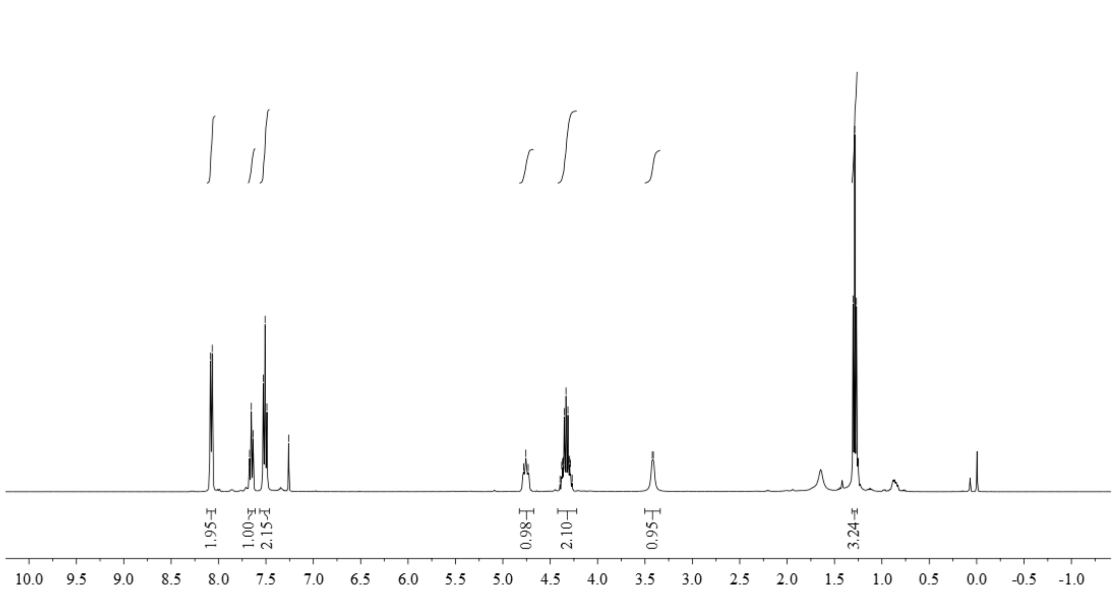

^{19}F NMR (376 MHz, $CDCl_3$) of **3u**

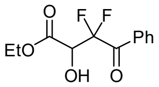

3u

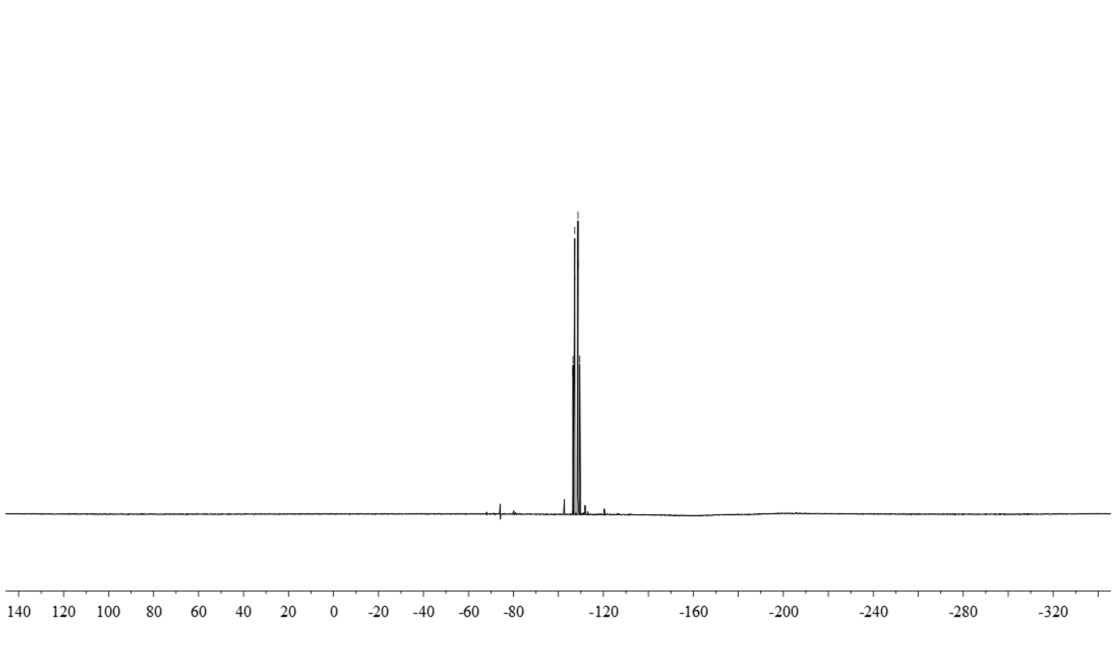

^{13}C NMR (100 MHz, $CDCl_3$) of **3u**

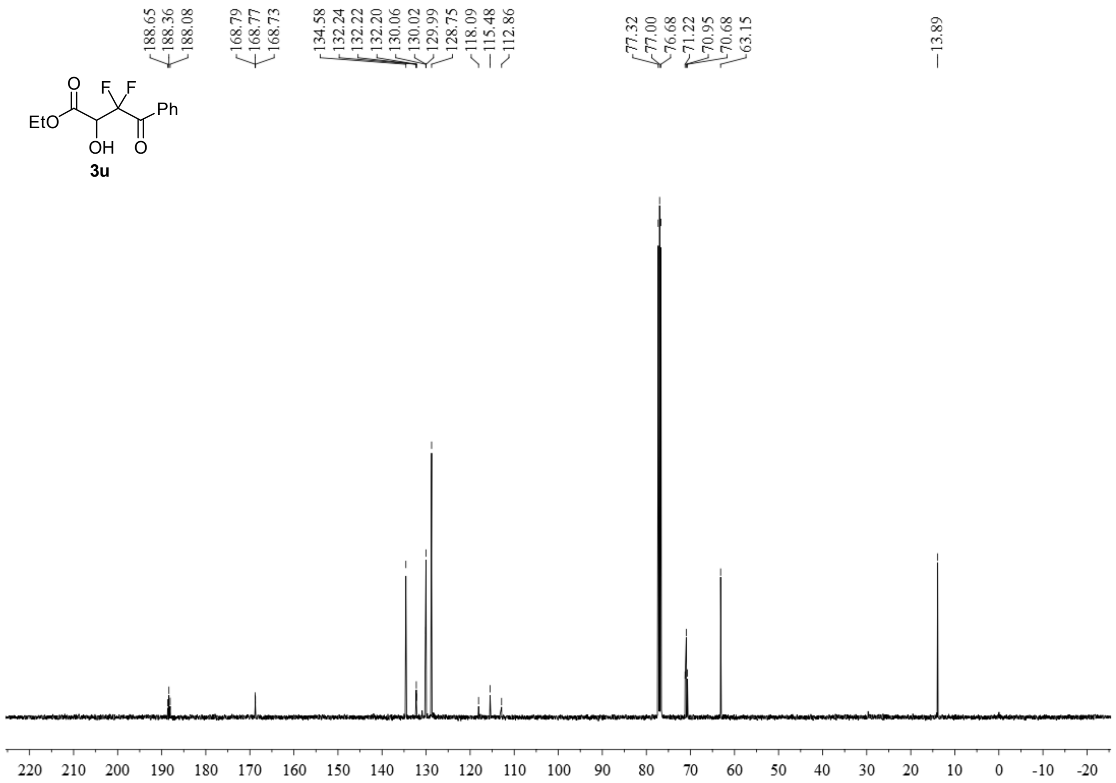

^{1}H NMR (400 MHz, $CDCl_3$) of **6a**

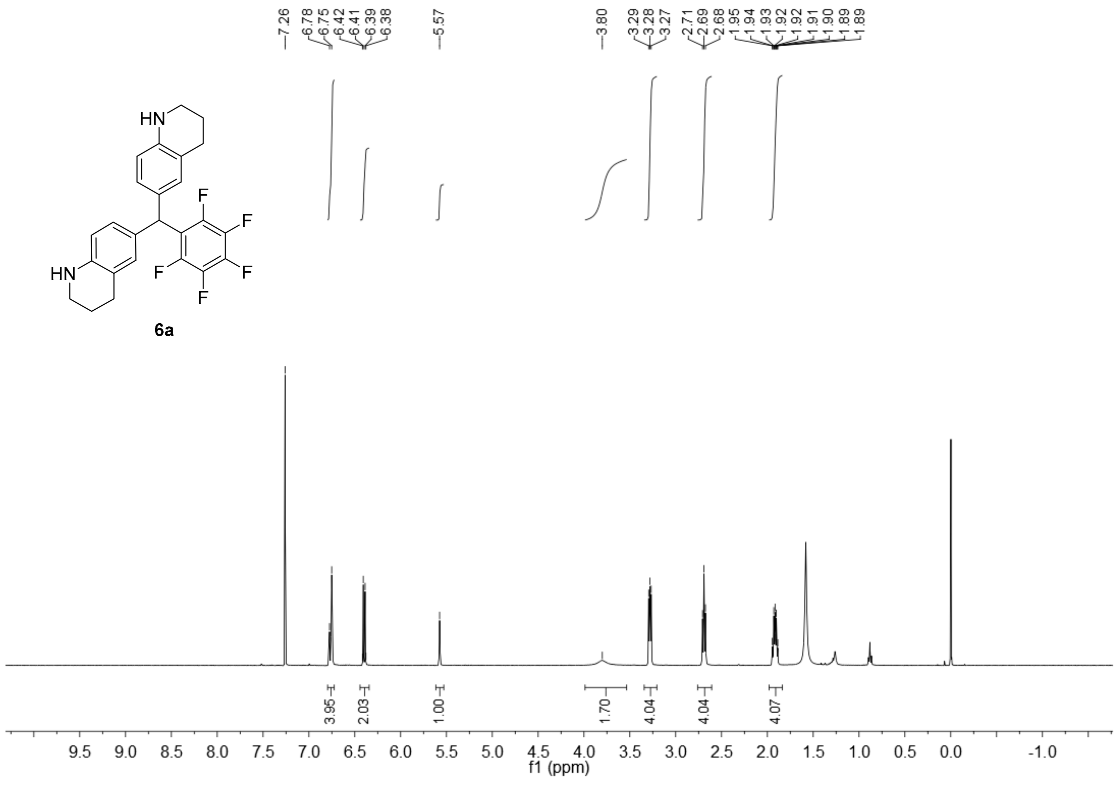

^{19}F NMR (376 MHz, CDCl$_3$) of **6a**

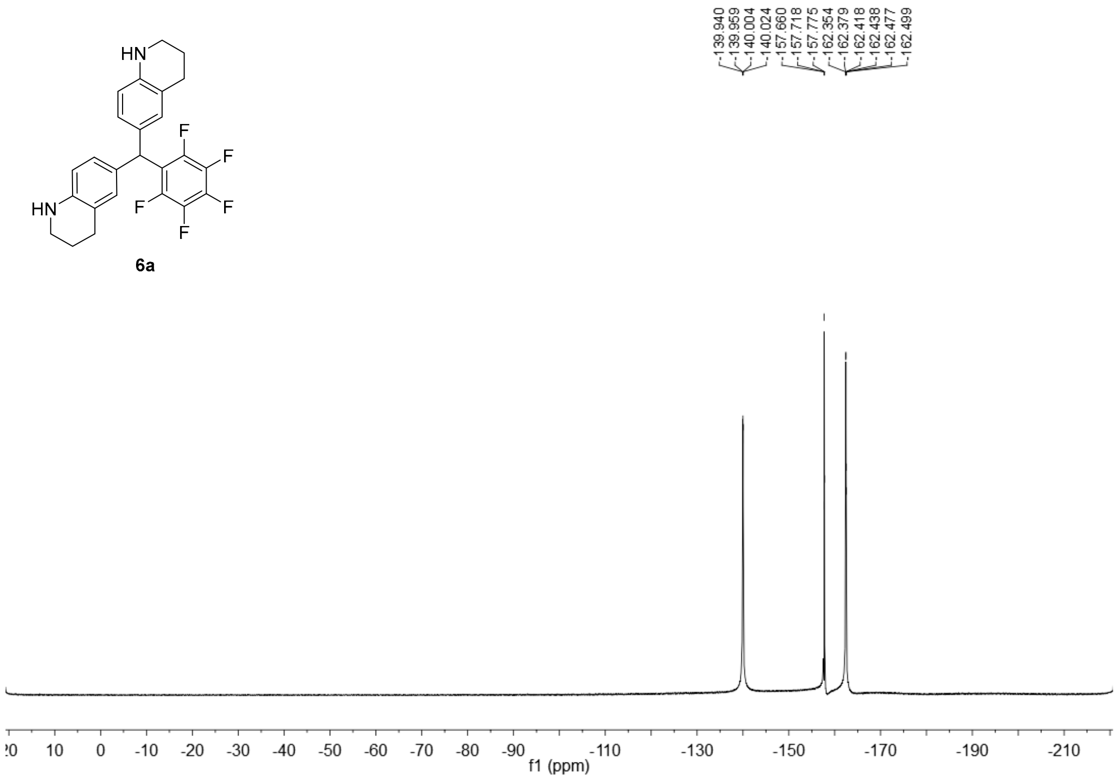

^{13}C NMR (100 MHz, CDCl$_3$) of **6a**

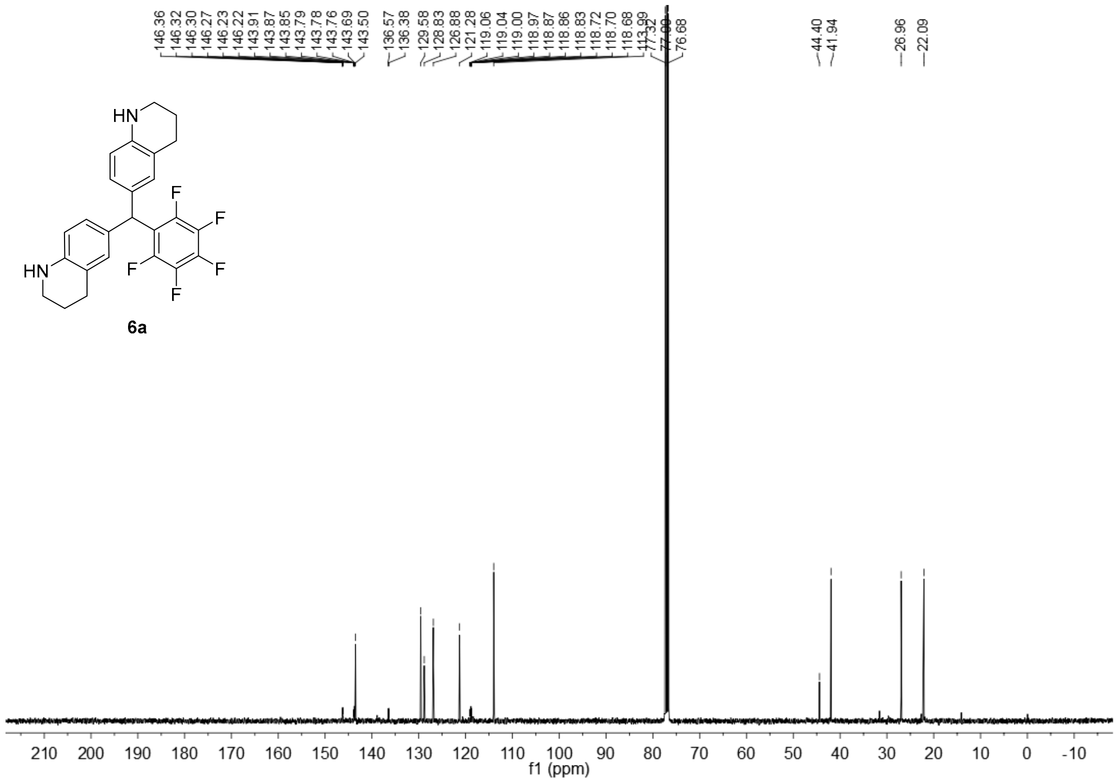

^{1}H NMR (400 MHz, $CDCl_3$) of **6b**

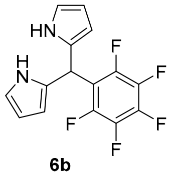

6b

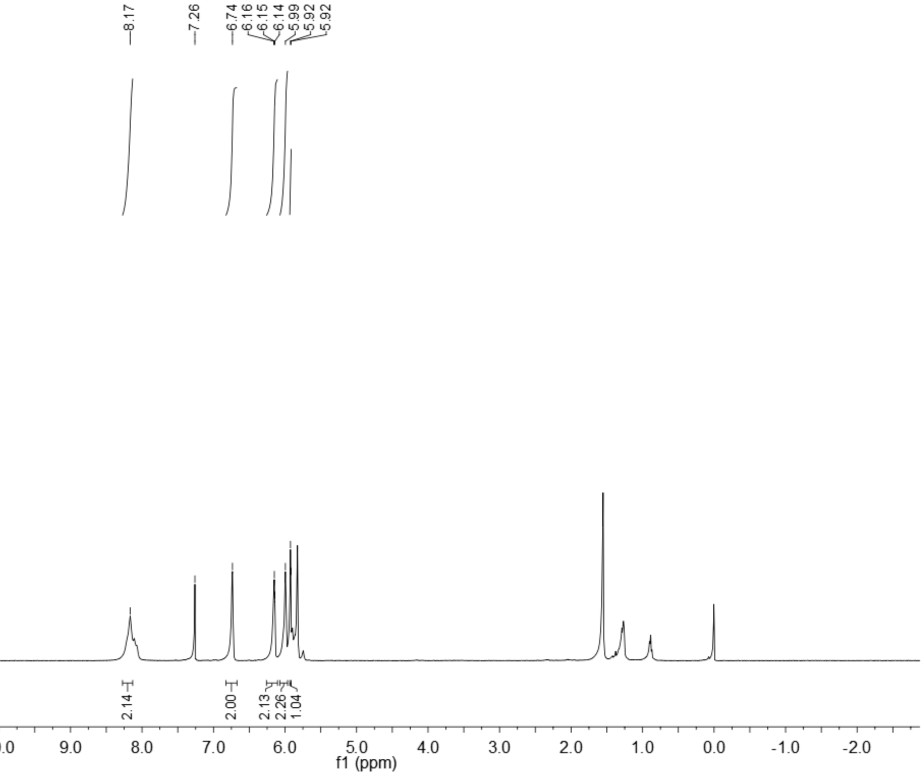

^{19}F NMR (376 MHz, $CDCl_3$) of **6b**

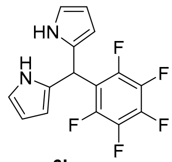

6b

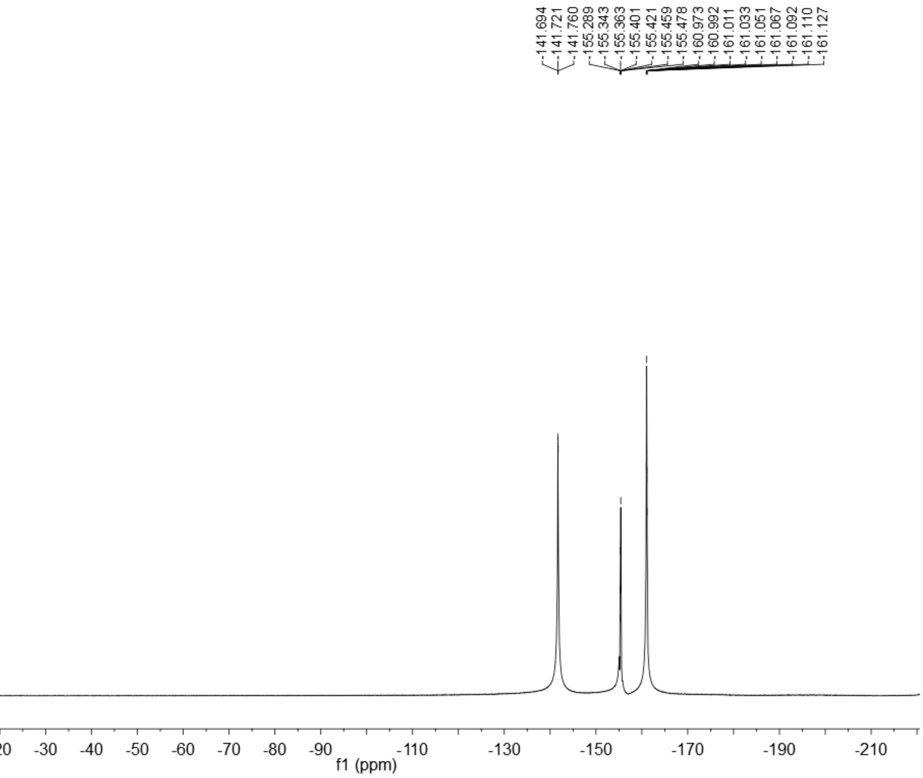

^{13}C NMR (100 MHz, $CDCl_3$) of **6b**

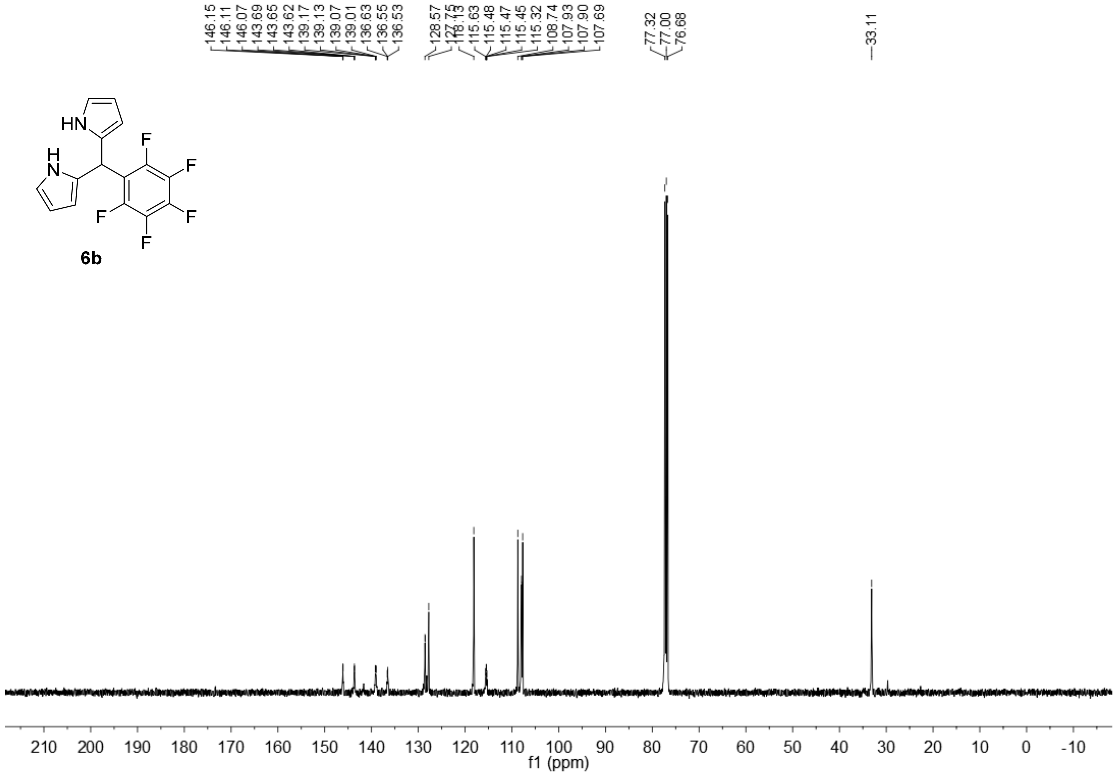

^{1}H NMR (400 MHz, $CDCl_3$) of **6c**

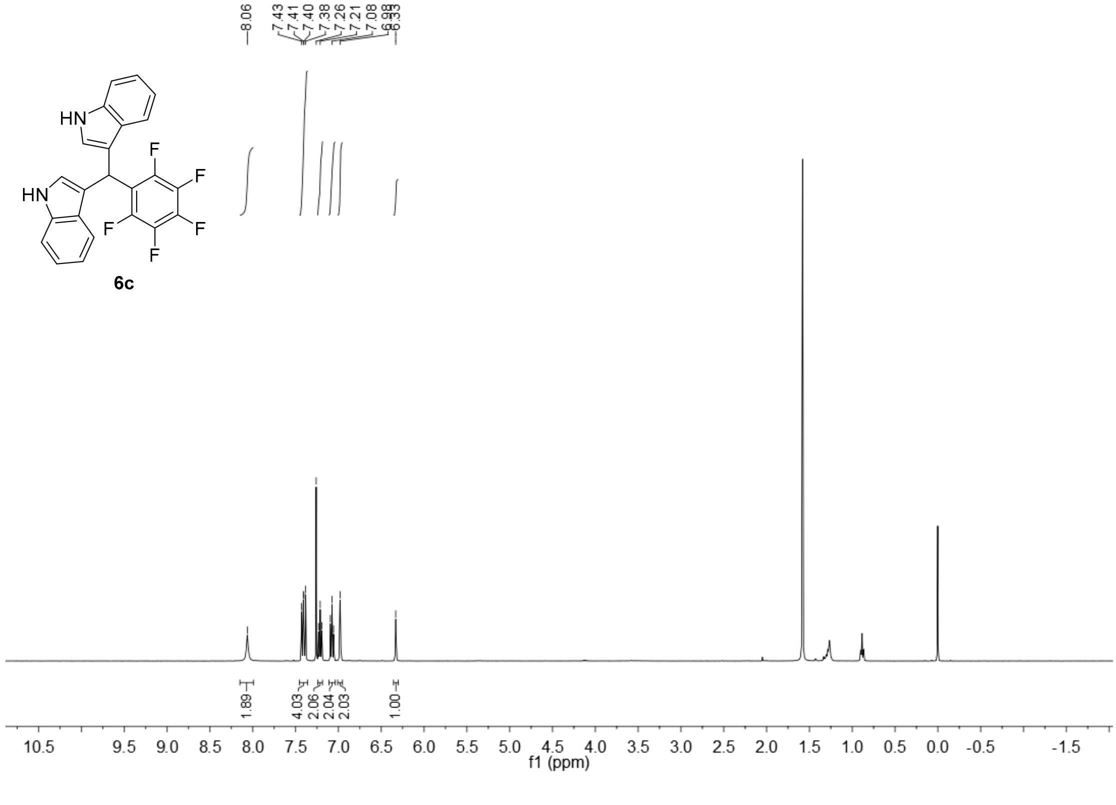

^{19}F NMR (376 MHz, $CDCl_3$) of **6c**

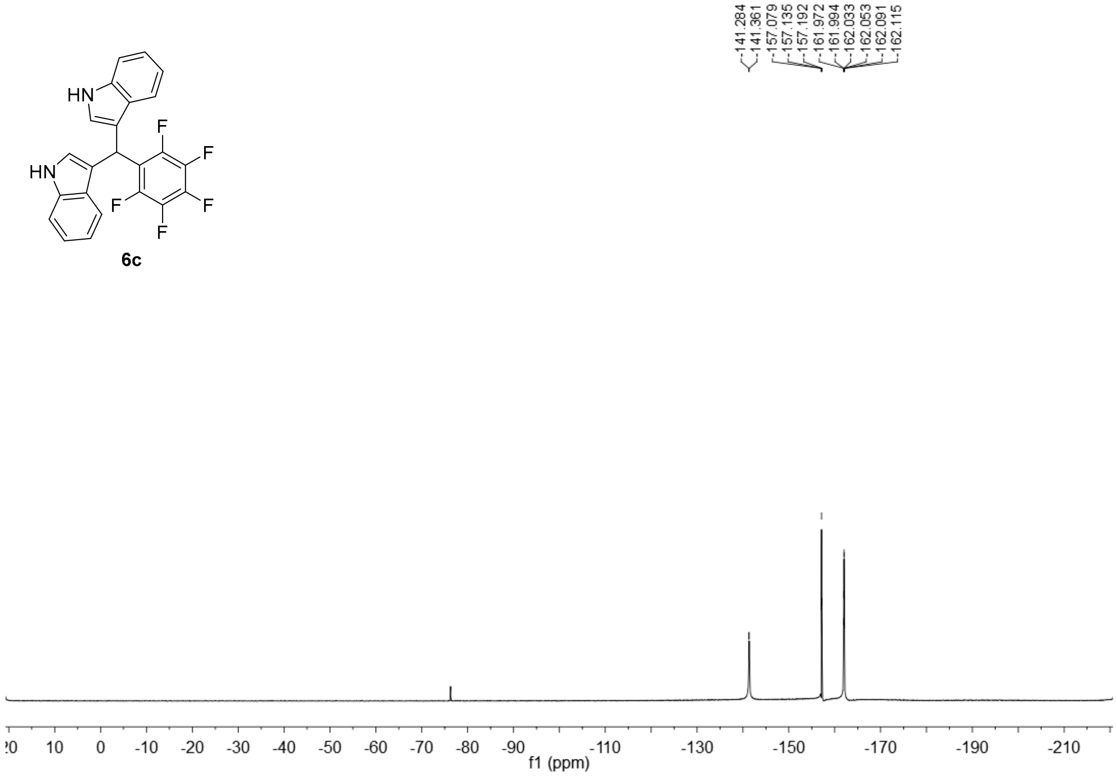

^{13}C NMR (100 MHz, $CDCl_3$) of **6c**

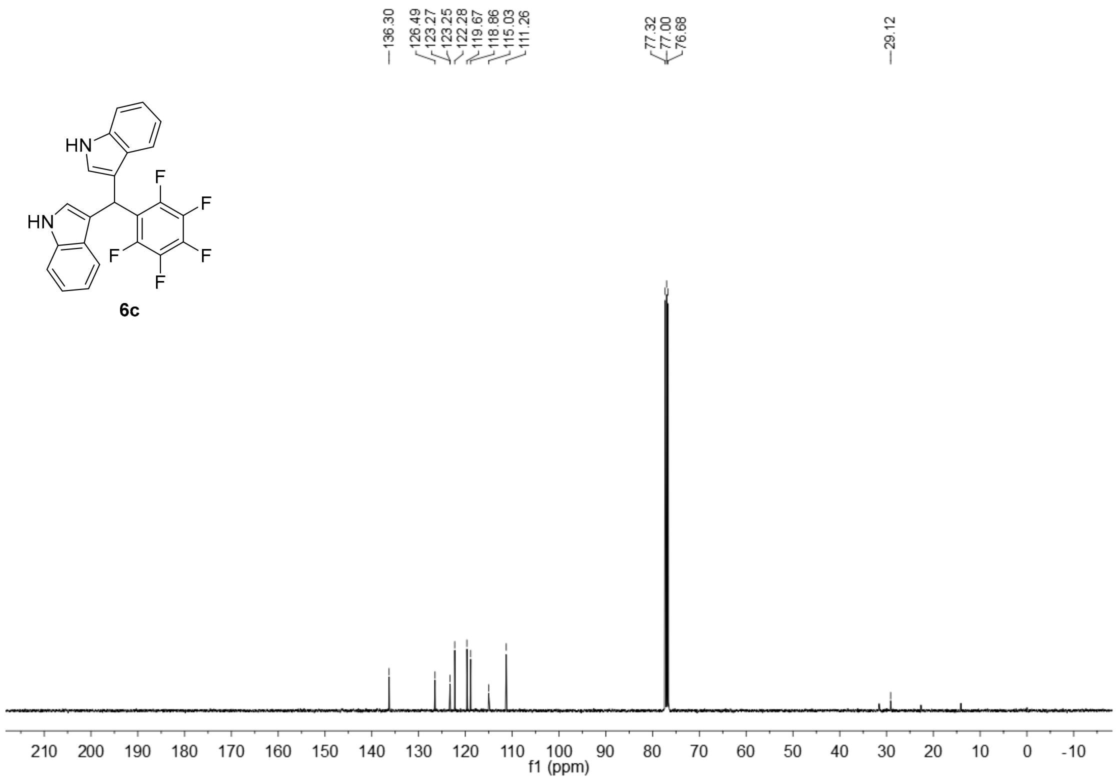

^{1}H NMR (400 MHz, Acetone-*d6*) of **6d**

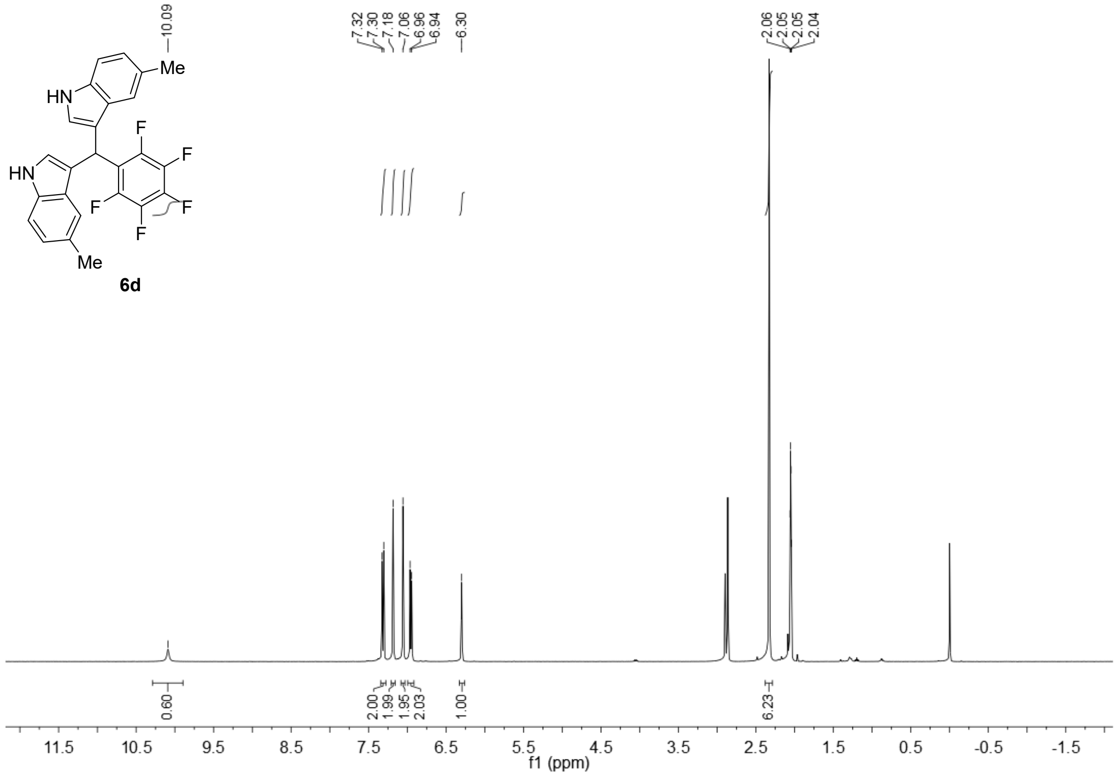

^{19}F NMR (376 MHz, Acetone-*d6*) of **6d**

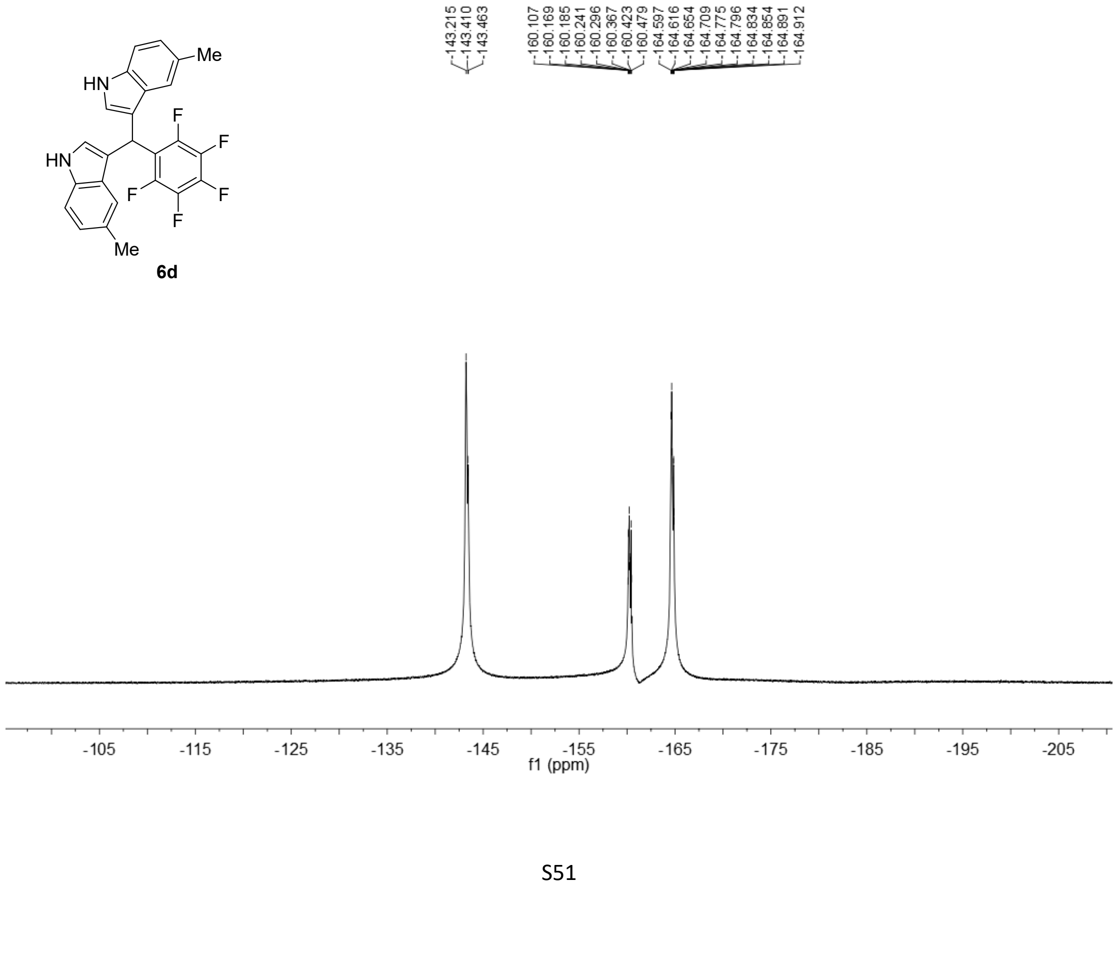

^{13}C NMR (100 MHz, Acetone-*d*6) of **6d**

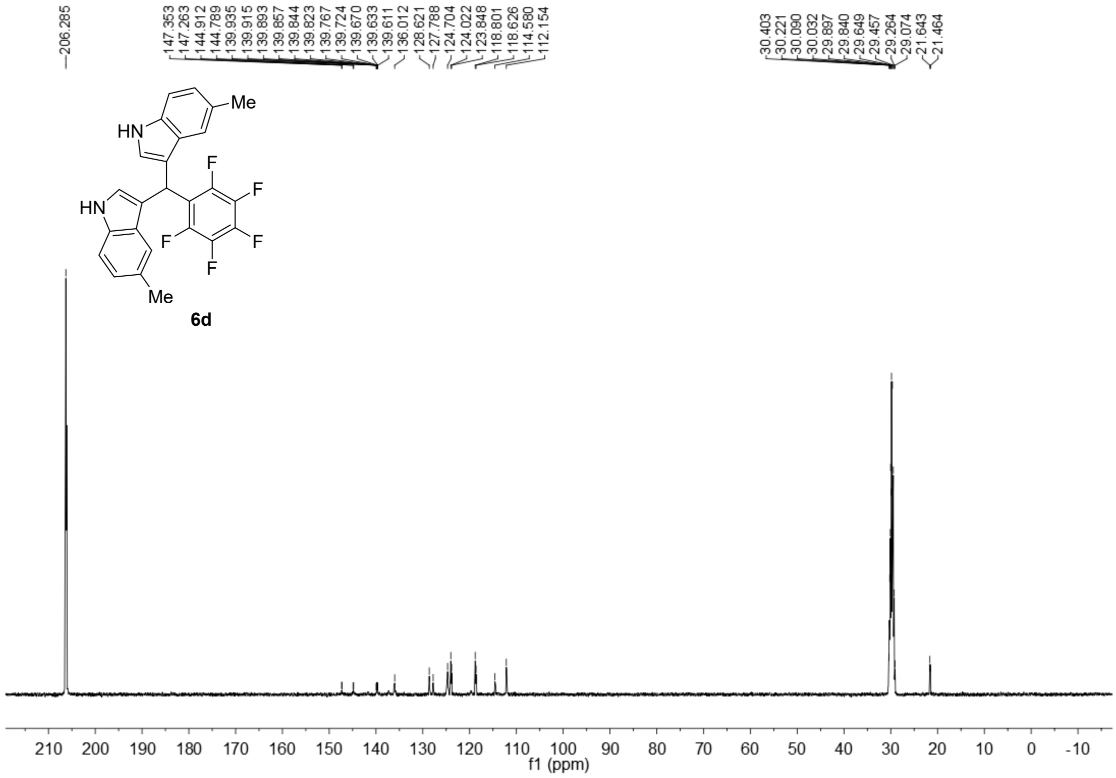

^{1}H NMR (400 MHz, Acetone-*d*6) of **6e**

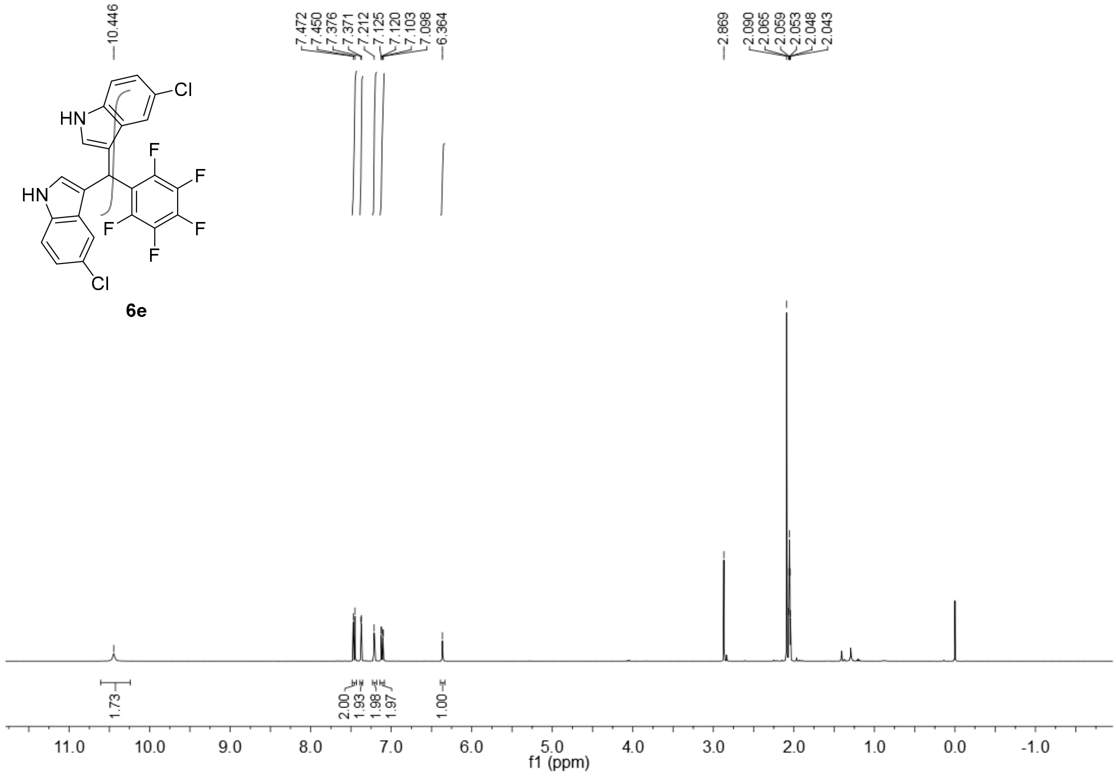

^{19}F NMR (376 MHz, Acetone-*d*6) of **6e**

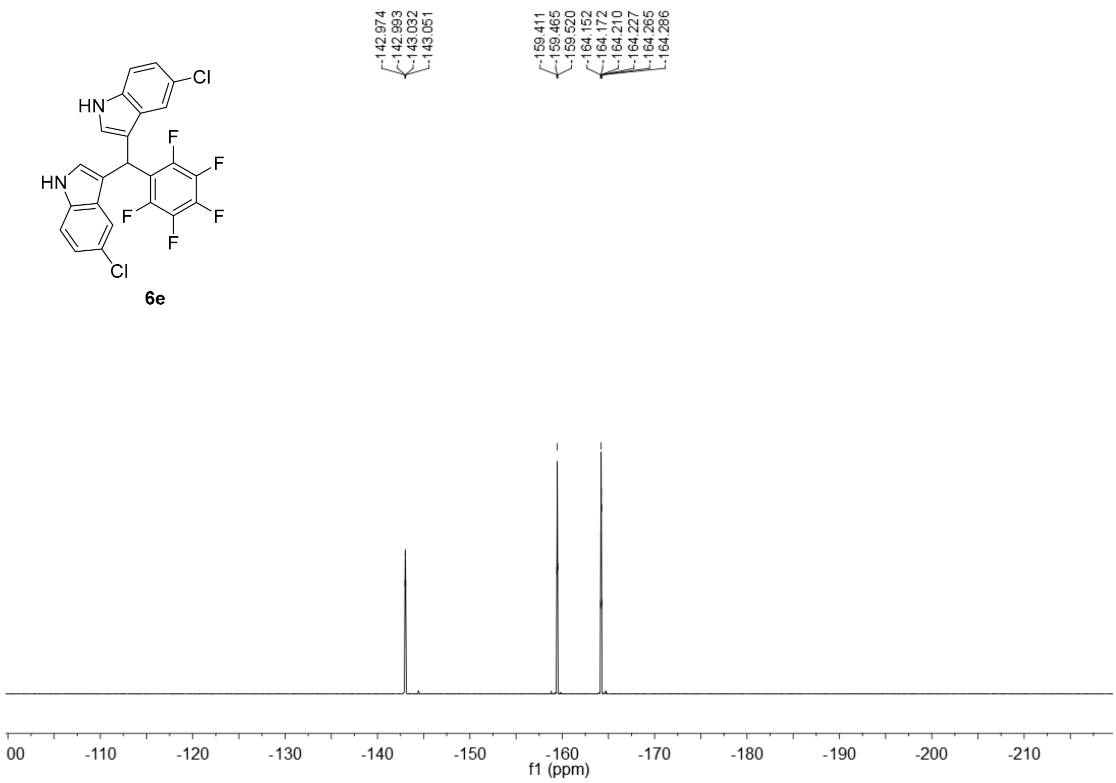

^{13}C NMR (100 MHz, Acetone-*d*6) of **6e**

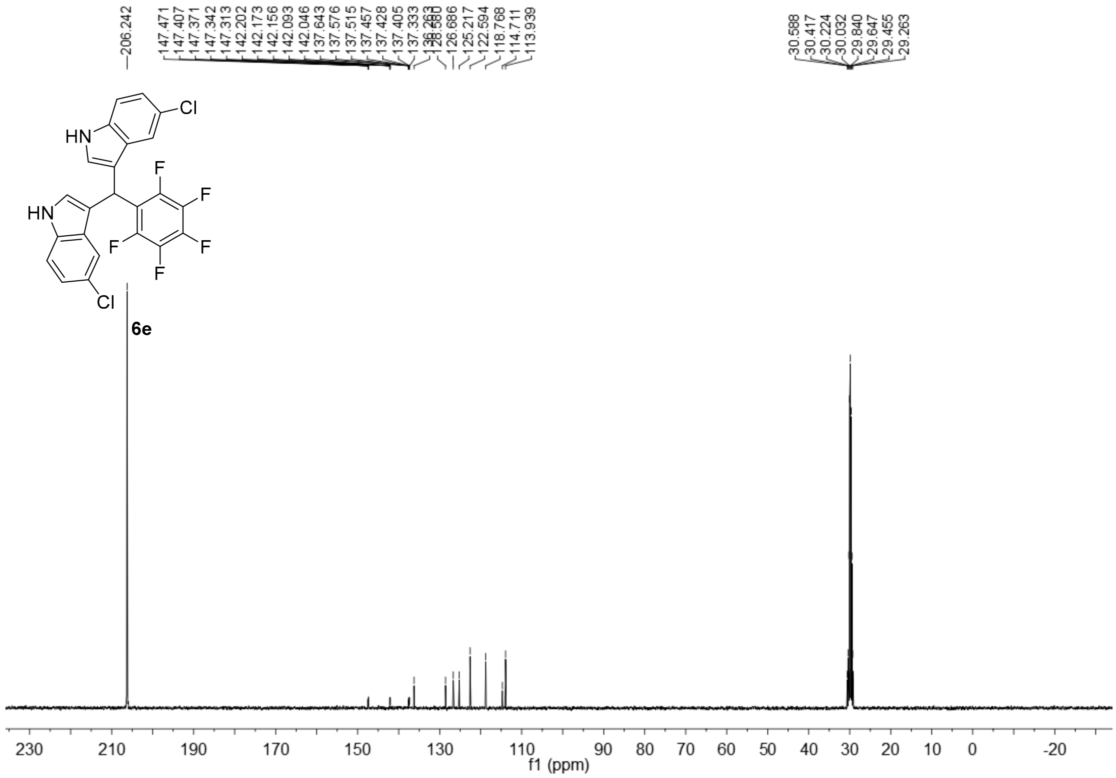

^{1}H NMR (400 MHz, Acetone-*d6*) of **6f**

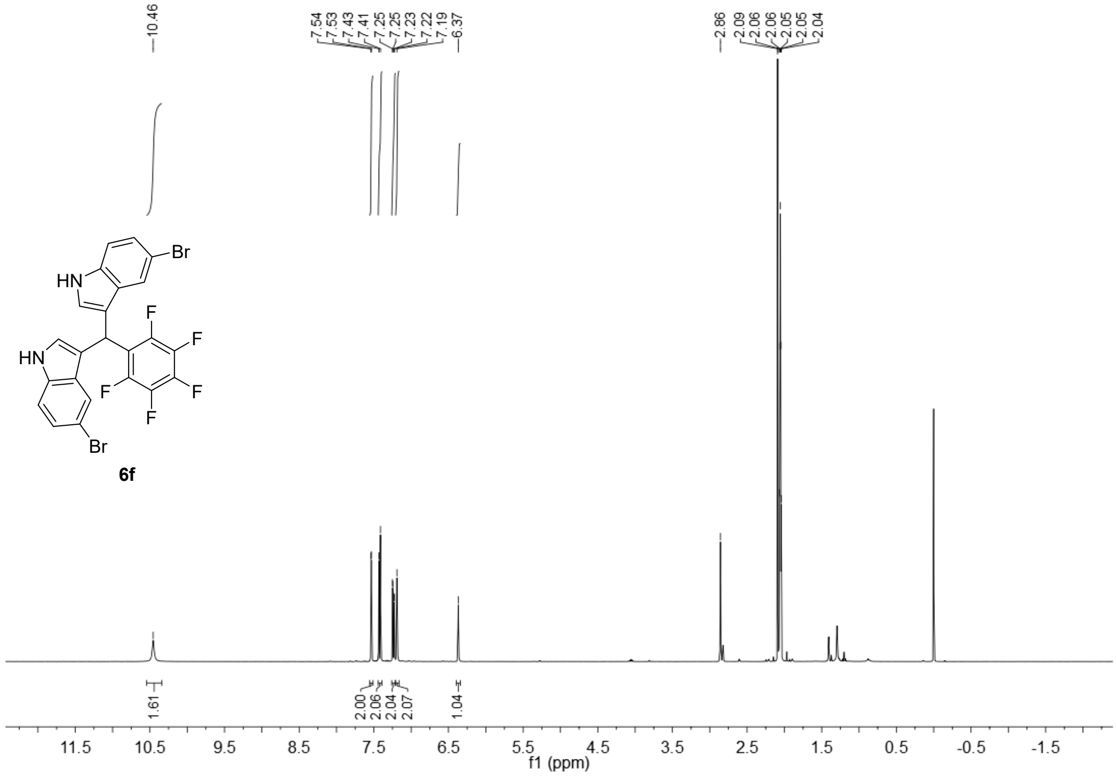

^{19}F NMR (376 MHz, Acetone-*d6*) of **6f**

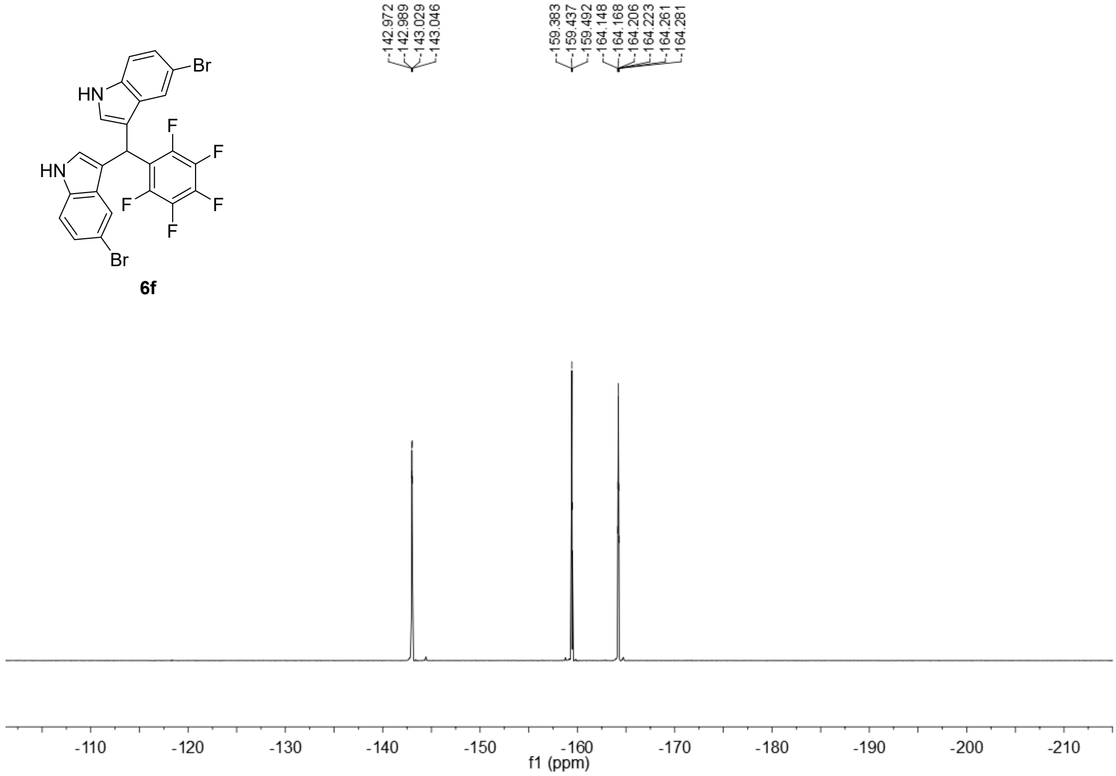

^{13}C NMR (100 MHz, Acetone-*d*6) of **6f**

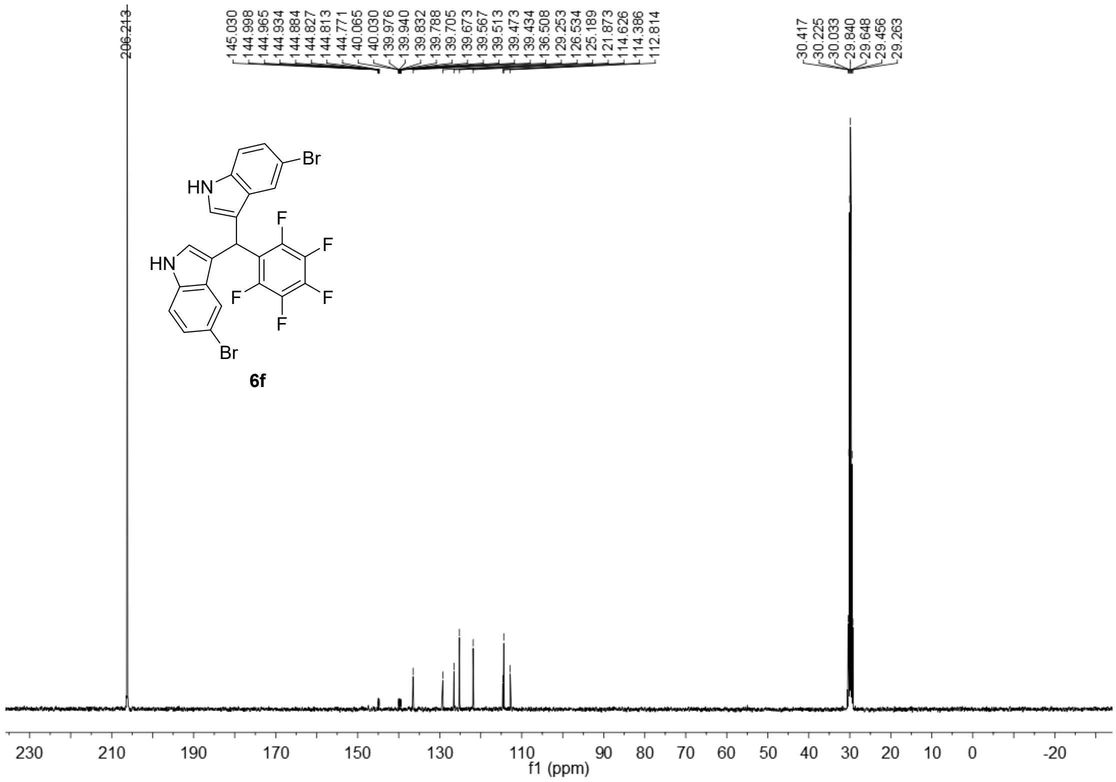

^{1}H NMR (400 MHz, $CDCl_3$) of **6g**

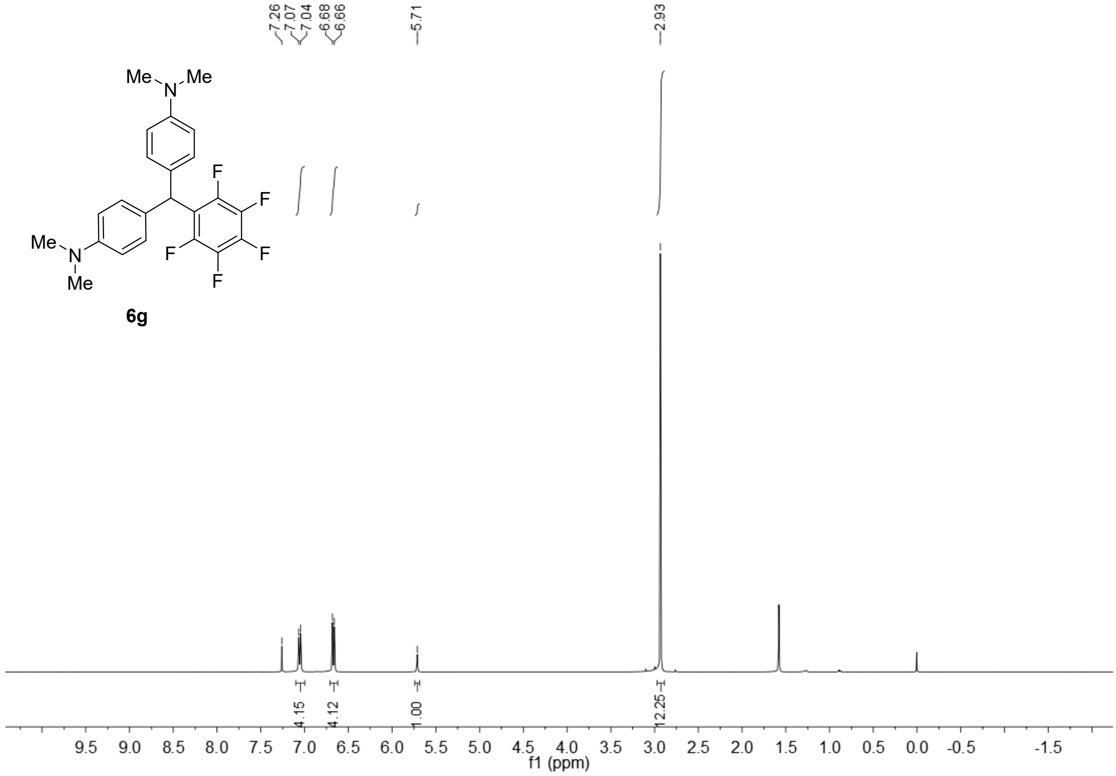

^{19}F NMR (376 MHz, $CDCl_3$) of **6g**

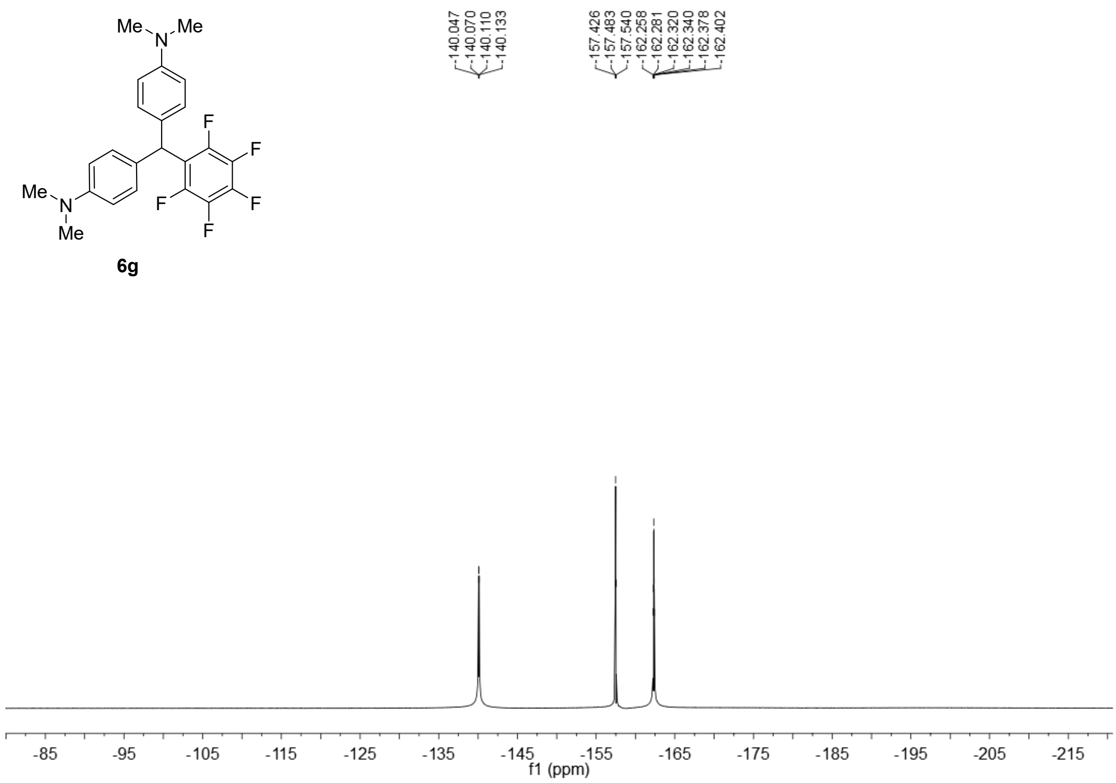

^{13}C NMR (100 MHz, $CDCl_3$) of **6g**

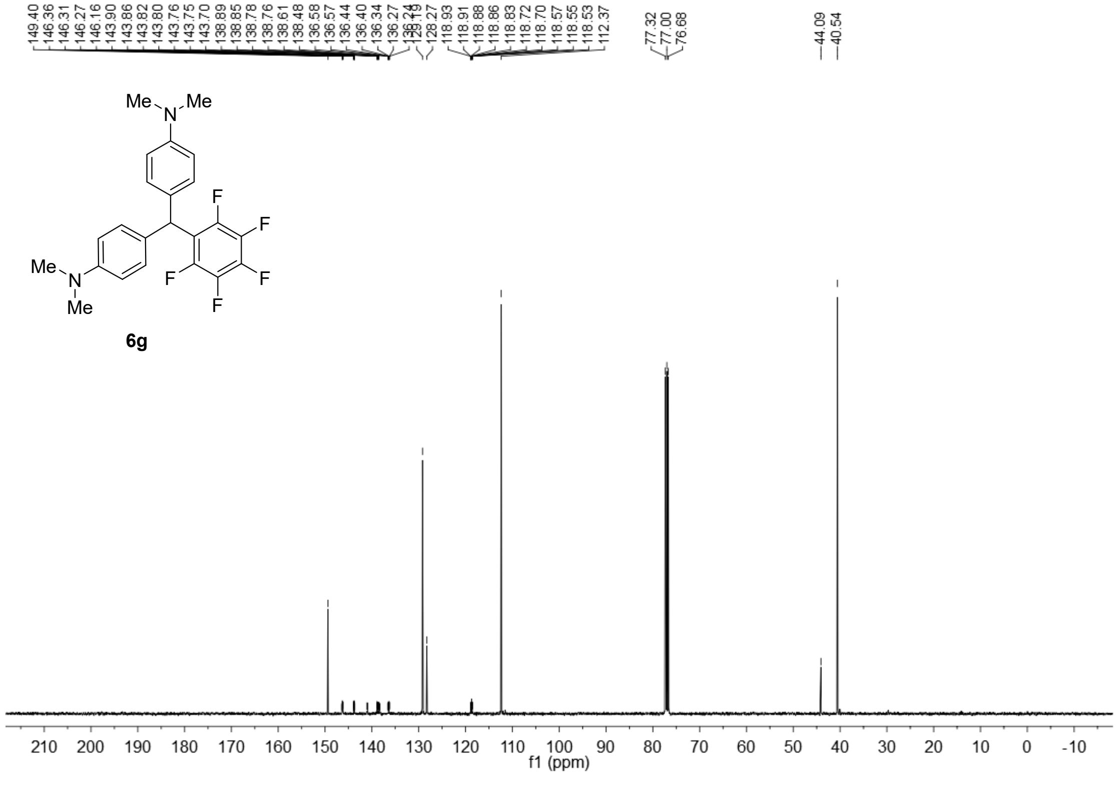

^{1}H NMR (400 MHz, $CDCl_3$) of **6g′**

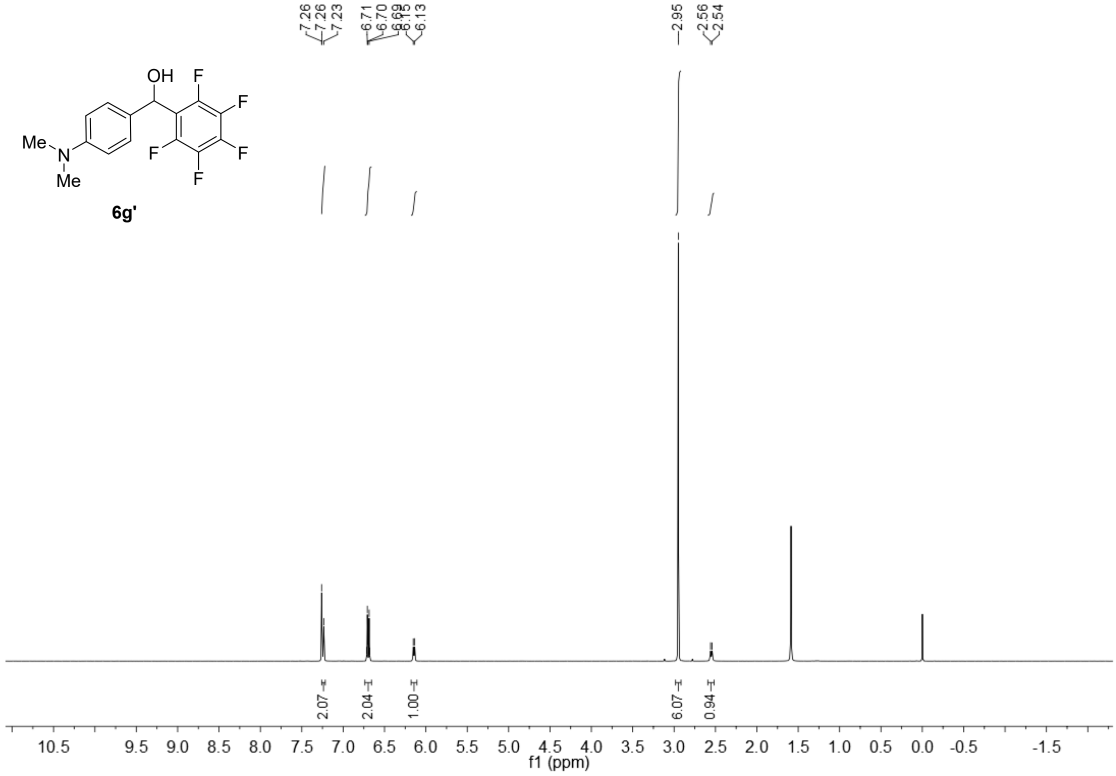

^{19}F NMR (376 MHz, $CDCl_3$) of **6g′**

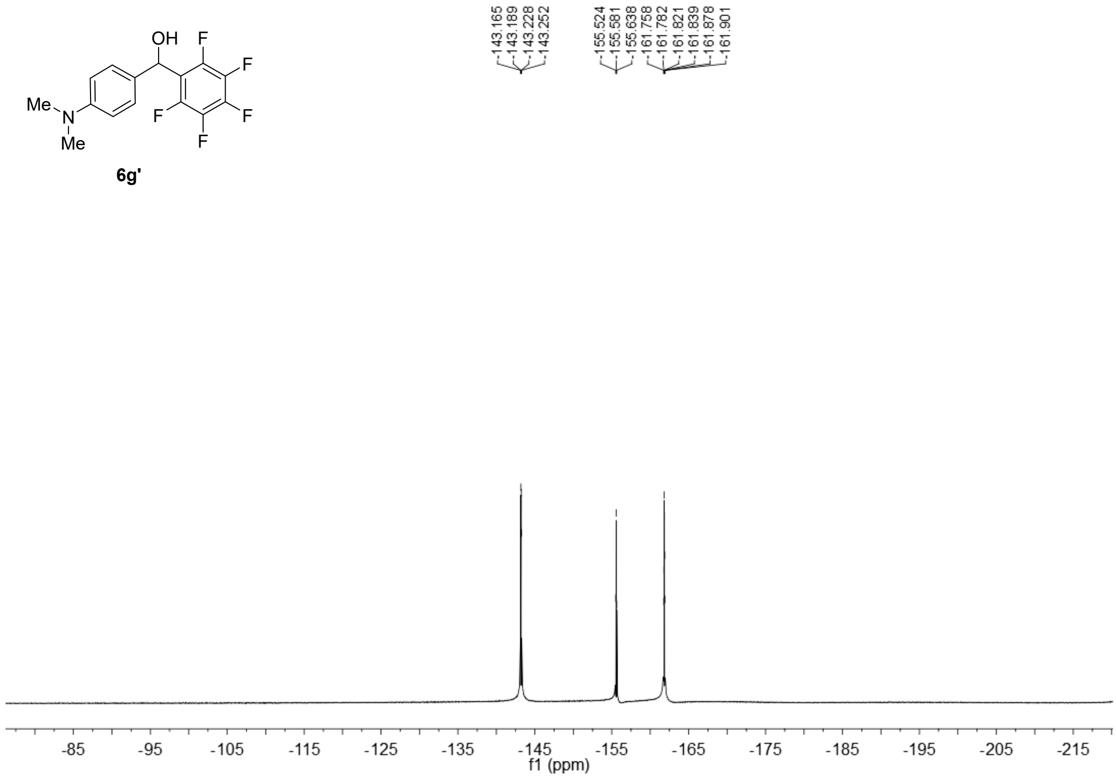

^{13}C NMR (100 MHz, CDCl$_3$) of **6g′**

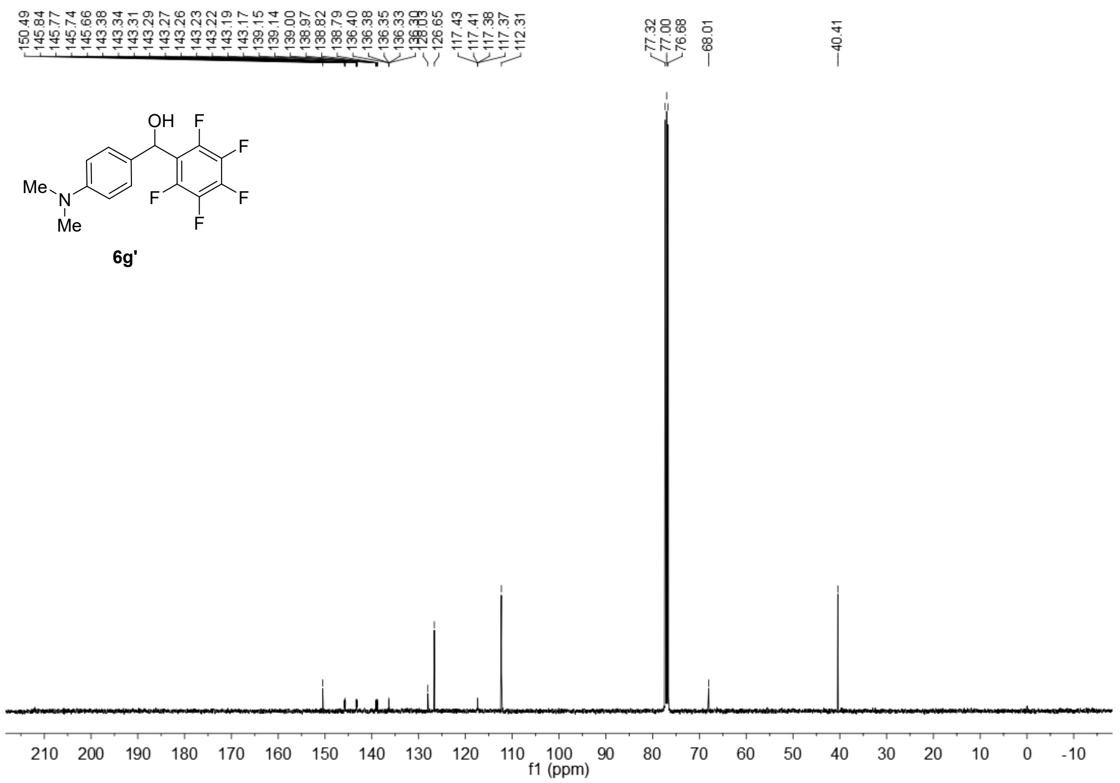

^{1}H NMR (400 MHz, CDCl$_3$) of **6h**

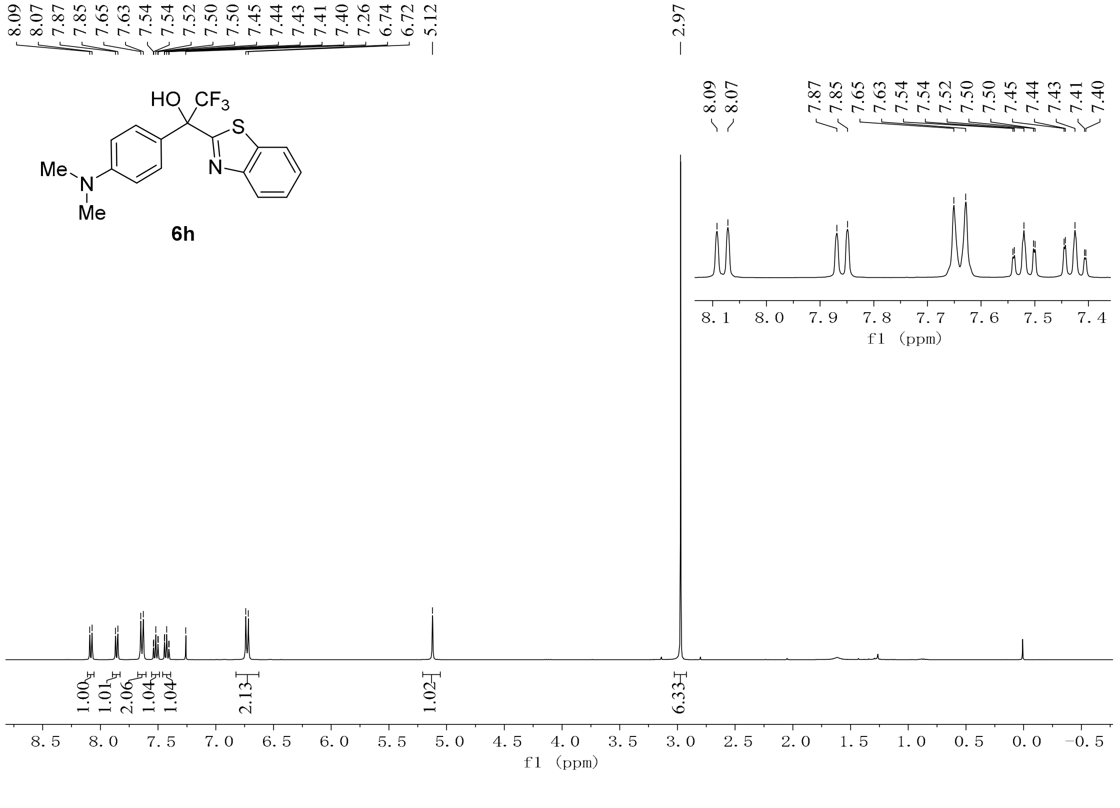

¹⁹F NMR (376 MHz, $CDCl_3$) of **6h**

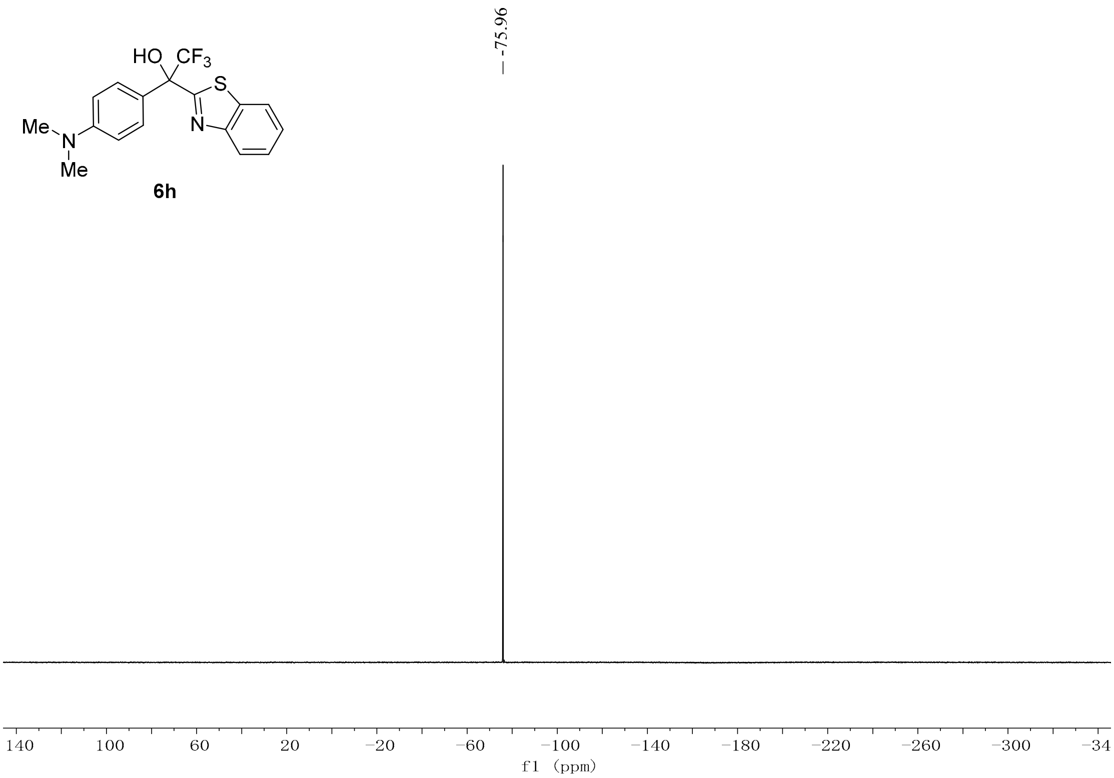

¹³C NMR (100 MHz, $CDCl_3$) of **6h**

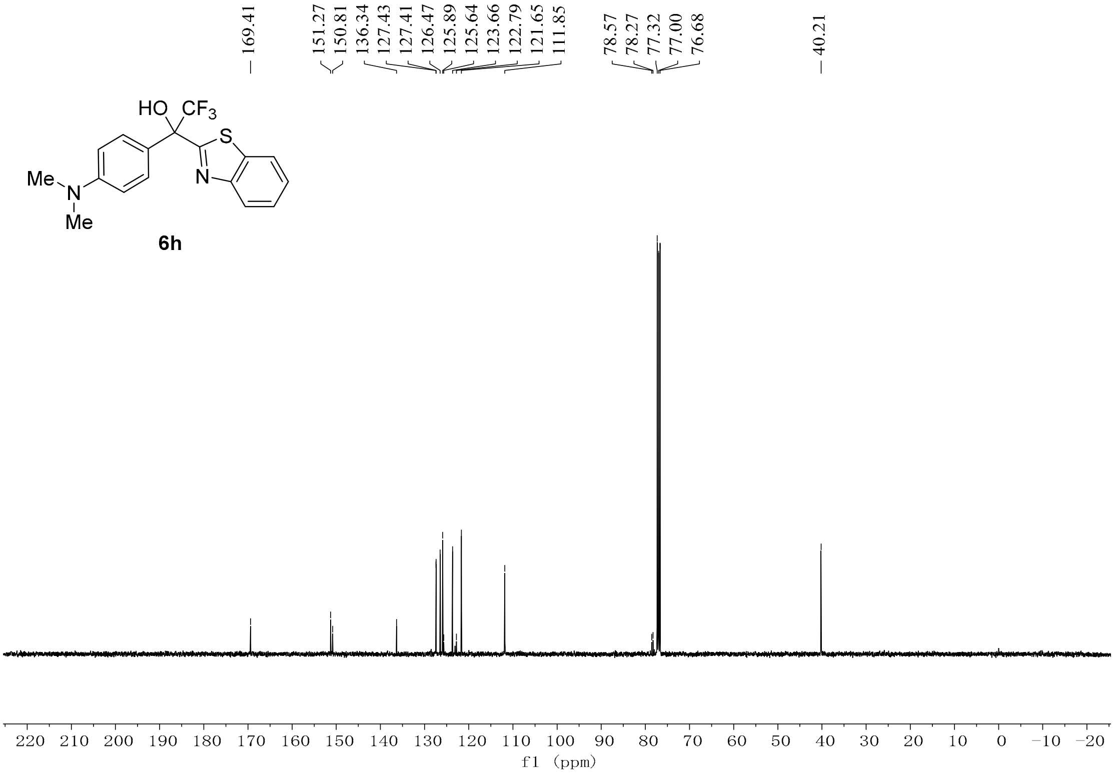

Supplement: Supplementary file 1 [file molecules-29-00697-s001.zip › molecules-2832743-supplementary.pdf]
